# Systematic identification of non-canonical transcription factor motifs

**DOI:** 10.1186/s12860-021-00382-6

**Published:** 2021-08-31

**Authors:** Luis Chumpitaz-Diaz, Md. Abul Hassan Samee, Katherine S. Pollard

**Affiliations:** 1 Biophysics Program, Stanford University, Stanford, CA USA; 2 Department of Molecular Physiology and Biophysics, Baylor College of Medicine, Houston,, TX USA; 3 Gladstone Institute of Data Science and Biotechnology, San Francisco, CA USA; 4 Department of Epidemiology & Biostatistics, Institute for Human Genetics, Quantitative Biology Institute, and Institute for Computational Health Sciences, University of California, San Francisco, CA USA; 5 Chan-Zuckerberg Biohub, San Francisco, CA USA

## Abstract

Sequence-specific transcription factors (TFs) recognize motifs of related nucleotide sequences at their DNA binding sites. Upon binding at these sites, TFs regulate critical molecular processes such as gene expression. It is widely assumed that a TF recognizes a single “canonical” motif, although recent studies have identified additional “non-canonical” motifs for some TFs. A comprehensive approach to identify non-canonical DNA binding motifs and the functional importance of those motifs’ matches in the human genome is necessary for fully understanding the mechanisms of TF-regulated molecular processes in human cells. To address this need, we developed a statistical pipeline for in vitro HT-SELEX data that identifies and characterizes the distributions of non-canonical TF motifs in a stringent manner. Analyzing ~170 human TFs’ HT-SELEX data, we found non-canonical motifs for 19 TFs (11%). These non-canonical motifs occur independently of the TFs’ canonical motifs. Non-canonical motif occurrences in the human genome show similar evolutionary conservation to canonical motif occurrences, explain TF binding in locations without canonical motifs, and occur within gene promoters and epigenetically marked regulatory sequences in human cell lines and tissues. Our approach and collection of non-canonical motifs expand current understanding of functionally relevant DNA binding sites for human TFs.

**Supplementary Information:**

The online version contains supplementary material available at 10.1186/s12860-021-00382-6.

## Introduction

Sequence-specific regulatory proteins, also known as transcription factors (TFs), are generally assumed to recognize a single motif of related nucleotide sequences at their DNA binding sites. Recent studies [18, 28], however, have shown that some TFs recognize motifs that are different from their single “canonical” motifs. This phenomenon of “non-canonical” motifs was first described in PBM (protein-binding microarray) data [3, 19], but later HT-SELEX (high-throughput systematic evolution of ligands by exponential enrichment) datasets suggested that motifs found in addition to the canonical motifs are not too distinct -- most often, those are due to a TF’s ability to dimerize [14] or due to minor sequence variations flanking the canonical motif [22]. Thus, questions remain open on whether non-canonical motifs are actually rare and whether their cognate sites in the human genome have any potential function.

In this work, we explore these questions through re-analysis of HT-SELEX data. We define a TF’s non-canonical motifs as sequence motifs that are significantly different from its canonical motifs, are at least as enriched as the canonical motifs in its in vitro DNA binding data, and can explain the TF’s binding in sequences where canonical motifs are absent. To investigate the existence of non-canonical motifs, we analyzed a recent high-quality in vitro HT-SELEX dataset of 169 human TFs [35]. We developed a statistical pipeline that applies a set of conservative criteria on these datasets and comprehensively tests for the existence of non-canonical motifs. For 19 out of 169 TFs (11%), we identify high-confidence non-canonical motifs that computationally validate in another recent HT-SELEX dataset [36]. By utilizing in vivo TF-DNA binding data, evolutionary conservation, and epigenetically marked regulatory sequences, we find that the non-canonical motif matches in the human genome have potential functional roles. Our analyses suggest that the existence, extent, and functional importance of non-canonical motifs are likely underestimated. Our approach is broadly applicable for identifying non-canonical motifs and assessing their functional relevance.

## Results

### Non-canonical motifs are not rare and occur independently of canonical motifs while showing similar enrichments

To systematically investigate the existence of non-canonical motifs in in vitro HT-SELEX data, we developed a pipeline combining the standard practices of HT-SELEX data modeling [29] with an additional set of conservative filtering criteria (Methods). Briefly, following Slattery et al.’s approach, we first compute the most likely length *L* for a TF’s motif (*effective length*). Next we rank the *L*-mers based on their enrichment in the TF’s HT-SELEX data. We then identify the *canonical L*-mers (the *L*-mers matching the TF’s CIS-BP motifs [33]) and the *non-canonical L*-mers (the *L*-mers that are as enriched as the canonical *L*-mers but do not match CIS-BP motifs). We then cluster the *non-canonical L*-mers into motifs and report the ones showing statistically significant differences from CIS-BP motifs. We impose additional criteria throughout the pipeline to ensure that any observed signal of non-canonical motifs is likely not an artifact of the HT-SELEX procedure [23]. Using this pipeline, we analyzed 169 high-quality HT-SELEX datasets that Yang et al. presented in their recent study [35]. Yang et al. resequenced these datasets from [14] at a significantly higher depth (on average ~10-fold increase in depth) and filtered the reads through a quality-control pipeline.

Our analysis revealed potential non-canonical motifs for 28 out of 169 TFs (Table 1, Supplementary Table 1). We validated these motifs against a second independent HT-SELEX dataset from Yin et al. [36], tested if they are enriched in in vivo ChIP-seq data [17, 37, 38], and examined them for extensions or dimers of CIS-BP motifs (Supplementary Text 1 and 2). Based on these analyses, we split the 28 motifs into three categories with different strengths of evidence for their being functional, non-canonical motifs. The top two categories include nineteen motifs, which we refer to as non-canonical motifs [17, 37, 38].

**Table 1:**
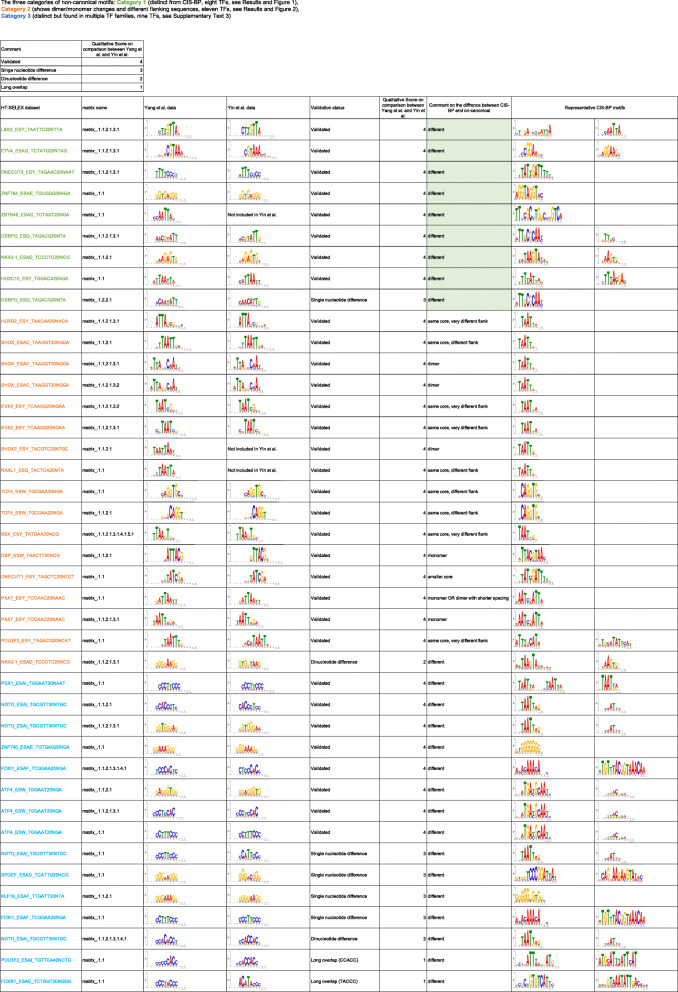
The three categories of non-canonical motifs. Shown are the non-canonical motifs discovered from Yang et al. data, the corresponding validated motifs from Yin et al. data and the CIS-BP motifs

**Fig. 1:**
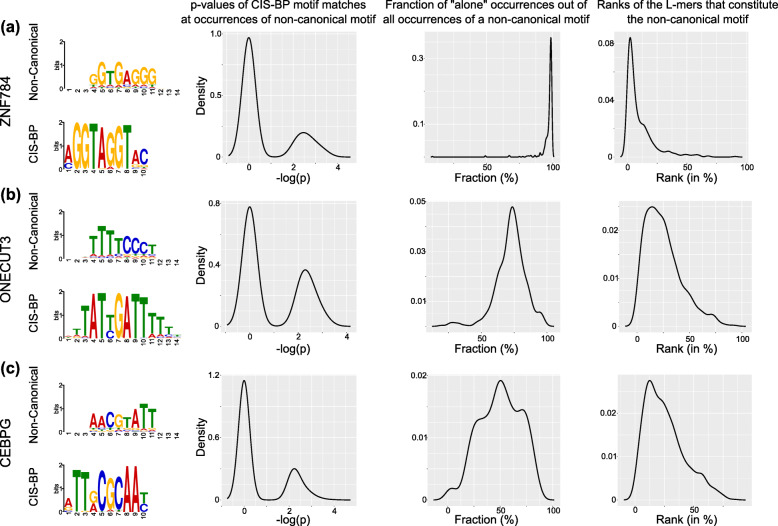
Examples of distinct non-canonical motifs and the corresponding CIS-BP motifs for the TFs ZNF784 (**a**), ONECUT3 (**b**), and CEBPG (**c**). In each panel, we also show three statistics related to the corresponding non-canonical motif (left to right): the distribution of *p*-values of CIS-BP motif matches that overlap the constituent *L*-mers of the non-canonical motif, the distribution of the fraction of oligos where the constituent *L*-mers occur alone, and the distribution of *L*-mer ranks according to enrichment. Motifs drawn with the program ceqlogo (MEME suite [32])

The top category includes eight non-canonical motifs that are truly distinct from their CIS-BP motifs. Unlike the examples mentioned in previous studies [14, 22], these non-canonical motifs could not be marked as dimers or as flanking sequence variations of the CIS-BP motifs. As examples, we show the non-canonical motifs of ZNF784 (Zinc Finger Protein 784), ONECUT3 (One Cut Homeobox 3), and CEBPG (CCAAT/enhancer-binding protein gamma) in Fig. 1. The ZNF784 non-canonical motif shown in Fig. 1a explains 9% of oligos, i.e., these oligos do not contain any significant matches to the CIS-BP motifs of ZNF784 (as identified by FIMO [10] at a *p*-value threshold of 10^−4^, see Methods). We repeated our motif occurrence analysis using FIMO at a very flexible *p*-value threshold of 10^−2^ and asked if any CIS-BP motif occurrences at this threshold overlaps with the *L*-mers that constitute the non-canonical motif of ZNF784. For those cases, we assign to the *L*-mer the *p*-value of the overlapping motif occurrence. For the other *L*-mers, where ZNF784’s CIS-BP motif did not overlap even at a *p*-value threshold of 10^−2^, we assigned them a *p*-value of 1. The density of motif match *p*-values at the *L*-mers that constitute the non-canonical motif of ZNF784 shows that the *L*-mers do not match any CIS-BP motif, and in about 20% cases, the matches to the CIS-BP motifs are very weak (*p*-value around 10^−2^) (Fig. 1a, left density plot). We also found that in almost all oligos where this non-canonical motif of ZNF784 occurs, it occurs alone as opposed to co-occurring with CIS-BP motifs (Fig. 1a, middle density plot). When ranked according to enrichment, these *L*-mers fall in the top 5% among all canonical and non-canonical *L*-mers (Fig. 1a, right density plot).

As a second example, we show the ONECUT3 non-canonical motif (Fig. 1b) that explains 6% of oligos in the analyzed round of its HT-SELEX data. The density plots in Fig. 1b show that, similar to the example of ZNF784 non-canonical motif, the *L*-mers constituting ONECUT3 non-canonical motif either do not match CIS-BP motifs or show very weak match (*p*-value of around 10^−2^). We also quantified that in ~75% of oligos where this non-canonical motif occurs, it occurs alone as opposed to co-occurring with CIS-BP motifs. In terms of enrichment, these non-canonical *L*-mers are ranked within the top 10% of all *L*-mers.

Finally, we show a non-canonical motif of CEBPG (Fig. 1c) that explains 5% of oligos in the analyzed round of its HT-SELEX data. The density plots in Fig. 1c show that, similar to the other two examples, the *L*-mers constituting this non-canonical motif generally do not match CIS-BP motifs or show very weak match (*p*-value of around 10^−2^). Also, these *L*-mers occur mostly alone as opposed to co-occurring with CIS-BP motifs, and they rank within the top 10% of all *L*-mers. Together, these examples and the other five motifs in the top category are strong evidence for the presence of non-canonical motifs for a small subset of well-studied TFs.

### Non-canonical motifs reveal new dimer-monomer patterns, spacer sequences, and long yet specific flanking sequences

The second category contains the remaining 11 non-canonical motifs. These are less distinct from CIS-BP motifs and could be marked as variations in dimerization or flanking sequences, consistent with current understanding of non-canonical motifs. Nine of them are enriched in ChIP-seq data. Despite being less unique from canonical motifs, these examples reveal novel binding mechanisms currently unknown from CIS-BP motifs. We discuss the specific cases below, all examples are shown in detail in Table 1 and Supplementary Table 1.

**Fig. 2:**
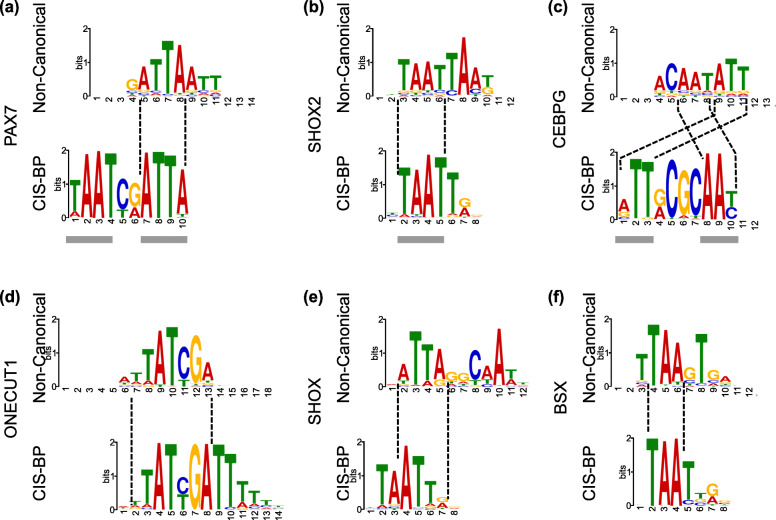
Examples of Non-Canonical motifs and the corresponding CIS-BP motifs for the TFs PAX7, SHOX2, CEBPG, ONECUT1, SHOX and BSX. Similar (alignable) regions of CIS-BP and non-canonical motifs are shown using dashed lines. For differences in monomers and dimers, the monomers and dimers in the CIS-BP motifs are shown with gray underlines. Motifs drawn using the program ceqlogo (MEME suite [32])

1. The non-canonical motifs of PAX7 (Paired Box 7) and DBP (D-Box Binding PAR BZIP Transcription Factor) suggest the TFs’ DNA-binding as monomers, but the CIS-BP motifs represent their binding as dimers (Fig. 2a). Also, the sequences flanking the core in the monomer motif are distinct from those in the CIS-BP motifs.
2. The case is opposite for SHOX2 (Short Stature Homeobox 2) (Fig. 2b) and HOXC10 (Homeobox protein C10), where the non-canonical motif represents the TF’s DNA-binding as a dimer, but the CIS-BP motifs represent its binding as a monomer.
3. The non-canonical motif of CEBPG (CCAAT/enhancer-binding protein gamma) represents a different dimerization pattern and a different spacer sequence than those in its CIS-BP motif (Fig. 2c). Note that these new dimerization patterns indicate a flip in the 5′-to-3′ placement of the monomers; they are not simply the reverse complements of the CIS-BP motifs.
4. For ONECUT1 (One Cut Homeobox 1), the non-canonical motif defines a shorter core motif than the CIS-BP motifs, suggesting that some flanking sequences in the CIS-BP motifs could be dispensable for its DNA binding in some contexts (Fig. 2d).
5. Finally, some non-canonical motifs share a TF-family specific core sequence with the canonical motifs, but show major differences in the flanking sequences. For example, the non-canonical motif of SHOX (Short Stature Homeobox) shares the HOX family specific “ATTA” sequence [25] with its CIS-BP motif, but clearly differs in six of the 10 flanking positions (Fig. 2e). The non-canonical motif of BSX (Brain Specific Homeobox) presents a similar case (Fig. 2f). This is important to note that the core sequences in our non-canonical motifs are often not as long as the cases shown in a previous study [22]. In the previous study, the core sequence was ~8 nucleotides long and the non-canonical motif differed in ~4 flanking nucleotides. In our examples, however, we found that the core sequences are shorter (~4 nucleotides) and the flanking sequences are often longer.

### Motifs enriched for multiple TFs

The third category includes nine motifs that are enriched in the HT-SELEX data for two or more TF families (Supplementary Text 3). This repeated occurrence is suggestive of a technical artifact in HT-SELEX. However, all nine potential non-canonical motifs are enriched in ChIP-seq data and pass our filtering criteria, which are similar or more stringent that criteria used to avoid HT-SELEX artifacts in previous studies [1, 35]. Nonetheless, we refer to these as *potential* non-canonical motifs and treat them separately from the nineteen non-canonical motifs (categories one and two) in the following genomic analyses.

### Non-canonical motifs explain in vivo ChIP-seq peaks where canonical motifs are absent

**Fig. 3:**
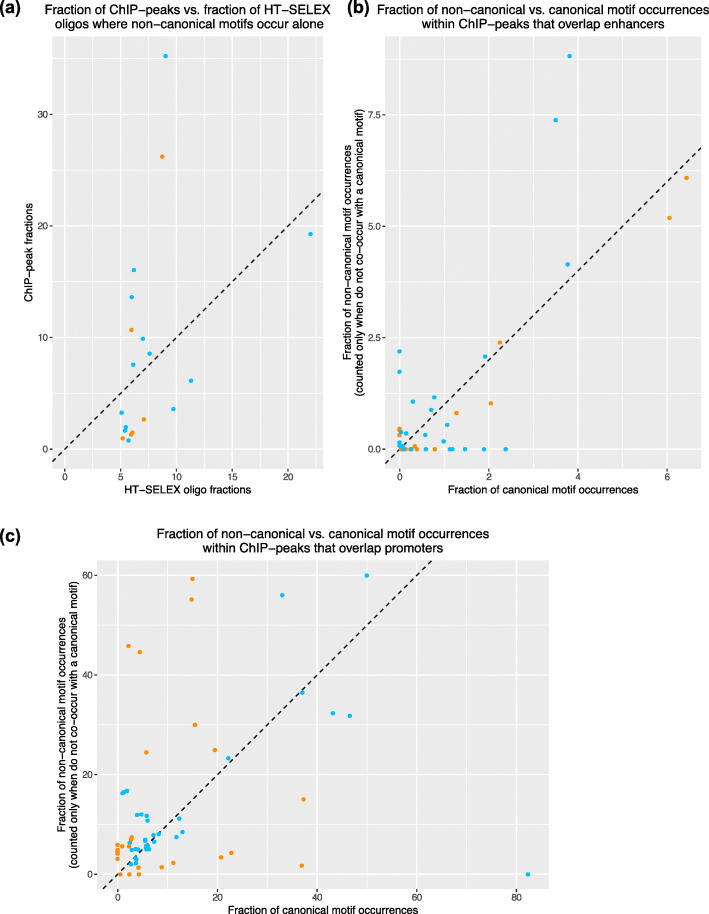
**a** Scatterplot showing the fractions of ChIP-seq peaks and the fractions of HT-SELEX oligo where a non-canonical motif occurs alone. The fractions were averaged over all ChIP-seq datasets of the corresponding TF. Each data point corresponds to a non-canonical motif. Orange data points correspond to potential non-canonical motifs with consensus sequences that are not family-specific (Supplementary Text 3). **b** Scatterplot showing the fractions of non-canonical and canonical motif occurrences (within ChIP-peaks) that are regulatory. Each data point corresponds to a 2-tuple consisting of a TF and a Cistrome ChIP-seq dataset. Orange data points correspond to potential non-canonical motifs. **c** Same information as shown in panel (**b**), but from promoter regions

We asked whether non-canonical motifs occur within in vivo TF-occupied regions, particularly in the regions where canonical motifs are absent but the data suggests TF-occupancy. For each TF, we computed the fraction of its ChIP-seq (Chromatin immunoprecipitation with massively parallel DNA sequencing) peaks where its non-canonical motifs occur alone, i.e., do not co-occur with its canonical motifs. To this end, we collected human ChIP-seq datasets from the Cistrome Data Browser [17, 38]. Fourteen TFs with motifs identified by our pipeline have ChIP-seq data in Cistrome. This includes seven TFs with non-canonical motifs (categories 1 and 2) and six TFs with potential non-canonical motifs (category 3). For each motif of a TF, we computed the mean fraction of ChIP-seq peaks (across all datasets; each TF has several ChIP-seq datasets in Cistrome) where the non-canonical motif occurs alone. As expected, the fractions of ChIP-peaks with only non-canonical or potential non-canonical motif occurrences are small compared to the peaks with canonical motif occurrence. However, these fractions are positively correlated with the fraction of HT-SELEX oligos where our motifs occur alone (Pearson correlation coefficient = 0.41; Fig. 3a). For 7/14 TFs (TCF4, ZNF784, KLF16, ZBTB49, ZNF740, POU3F2, NKX3-1), our motifs are more abundant in ChIP-peaks than they are in HT-SELEX oligos. For the other TFs (HOXB2, ATF4, DBP, ETV4, SPDEF, PDX1, and CEBPG), our motifs are more abundant in HT-SELEX oligos than in ChIP-peaks. Overall, this analysis suggests that non-canonical motifs are common in vivo and they often explain TF-occupied regions where the canonical motif of the TF is absent.

### Non-canonical motif occurrences mark binding sites with potential regulatory function

We next asked if occurrences of our motifs within ChIP-seq peaks could play any regulatory role. We include all three categories of our motifs, with potential non-canonical motifs analyzed separately from the other two categories. For each of our motifs, we consider only those occurrences that do not co-occur with a canonical motif in the same ChIP-peak. We call a motif occurrence *regulatory* if it overlaps with an epigenetically marked regulatory sequence. In particular, for each Cistrome ChIP-seq dataset, we collected the regulatory sequence annotations in the corresponding tissue- or cell-type from Cao et al.’s recent study based on the ENCODE and Roadmap Epigenomics data [5, 6, 8, 27]. We then computed the fractions of occurrences of our motifs that are regulatory in each dataset and compared this to the corresponding fraction for canonical motifs of the same TF (Methods, Fig. 3b). The fractions were generally similar, but in about half of the datasets, the regulatory fractions of both types of motifs are very low with one of them being zero. This is not a characteristic of any particular TF or a type of motif. For example, all occurrences of ATF4 non-canonical and canonical motifs are regulatory in epithelial cell-lines, but not in mesenchymal stem cells. On the other hand, for TCF4, both types of motifs are regulatory in dendritic and embryonic stem cells. However, in colon cancer cells, only its canonical motif occurrences are regulatory. These results highlight the cell type specificity of TF activity, which we detect with both canonical and non-canonical motifs.

We next performed the same analysis on promoter regions of the human genome (Fig. 3c). Interestingly, for occurences of both our motifs and canonical motifs, the fractions are higher within promoter regions. As noted above, we have focused here only on the non-canonical motif occurrences within ChIP-peaks where we did not find an occurrence for the TF’s canonical motif. This does not preclude the two types of motifs to occur in the same promoter. Thus, we asked how often the non-canonical and the canonical motif occurrences are within the same promoter. The Jaccard statistic between promoters with non-canonical motif occurrences and those with canonical motif occurrences was only 0.009 on average (range: 0–﻿﻿0.05; we considered only those datasets where at least 10% of both types of motifs’ occurrences are in the promoters), implying that non-canonical motif occurrences are nearly exclusive of canonical motif occurrences in the human promoters. Overall, this analysis suggests that non-canonical motif occurrences in the human genome may play a functional role in transcriptional regulation.

### Non-canonical and canonical motif occurrences in the human genome are similarly conserved

We finally compared the evolutionary conservation of non-canonical and canonical motif occurrences in the human genome. We particularly focused on occurrences of our motifs in ChIP-peaks (Cistrome dataset [17, 38]) where canonical motifs are absent. We compared the 7-way (human and six vertebrates) phyloP scores [24] of these motif occurrences against occurrences of the TF’s canonical motifs (Methods). For the non-canonical motifs of eight of the 14 TFs mentioned above, the two groups of motif occurrences have similar phyloP scores in some or all ChIP-seq datasets (Supplementary Table 2). Examples of such TFs include DBP, ATF4, CEBPG, TCF4, NKX3-1, and ETV4. Overall, based on the evidence of evolutionary conservation, this analysis suggested that non-canonical motif occurrences of some TFs carry functional importance in the human genome.

## Discussion

Recent discoveries in TF-DNA binding specificity have highlighted that TFs integrate several types of information to identify their specific target sites [13, 30]. Some discoveries have also questioned the common assumption that a TF recognizes only a single sequence motif [28]. Indeed, early quantitative studies of TF-DNA binding specificity had indicated that some TFs recognize multiple distinct sequence motifs [3, 19], but later studies reported that such additional motifs are largely due a TF’s ability to dimerize or due to variations in the sequences flanking a core region of its binding sites [14, 22]. Here we revisit this question utilizing a recent high-quality HT-SELEX dataset of ~170 human TFs. Incorporating a set of conservative filtering criteria with the widely accepted strategies to model HT-SELEX data, we found that 11% of the analyzed TFs indeed recognize motifs that are significantly different from their currently known “canonical” motifs. We call these motifs the “non-canonical” motifs of these TFs. In the previous studies, this question was discussed using the terms “primary” and “secondary” motifs. We chose the terms canonical and non-canonical since when we find more than one secondary motif, there is no intuitive ranking for those motifs as secondary, tertiary, etc.

The three conservative criteria that we introduced in our pipeline (Methods) are meant to eliminate different artifacts of HT-SELEX data that previous studies have reported. For example, the filter on the minimum entropy of di-nucleotide frequencies took care of poly-A or poly-C sequences that can show spurious enrichment in HT-SELEX data [23]. The criteria of round-over-round enrichment ensures that our findings are based on sequences showing a consistent rise in enrichment. Finally, when counting the fraction of oligos explained by a non-canonical motif, we only consider those oligos where the non-canonical motif occurs alone. This eliminates the possibility that the non-canonical motifs have “piggy-backed” on the CIS-BP motifs by occuring in the same oligos.

Besides the three main criteria mentioned above, we also stipulated that the non-motif L-mers should be at least as enriched as the motif L-mers (Methods), whereas in principle, a non-canonical motif could be rarer in HT-SELEX than the TF’s canonical motifs. However, a sequence’s enrichment in HT-SELEX has been found to correlate well with its affinity [26, 35]. Since it is important that the non-canonical motifs are also plausible in terms of TF-DNA binding affinities, we wanted to avoid those binding sites that show weaker affinity than the currently validated motifs. As such, we chose to avoid rarer sequences. This is a conservative approach that likely will miss some non-canonical motifs. But we can be confident that the non-canonical motifs we report are bound and likely functional.

Of note, since the HT-SELEX experiments were performed on individual TFs, by design the experiments preclude the possibility of co-factor binding affecting the observed specificity signals. Nevertheless, it is useful to distinguish the current study from Slattery et al.’s study [29] showing cofactor binding influencing the canonical DNA binding specificity of Hox Proteins. We note that, Slattery et al. did not investigate the presence of non-canonical motifs as an inherent property of a TF (i.e., independent of the presence of a binding partner). Rather, their study established that the Hox proteins recognize variants of the canonical motif by utilizing co-binding with Exd (Extradenticle-Homothorax) to bind at different genomic loci. As such, Slattery et al. did not report motifs that are significantly different from canonical motifs. Also, Slattery et al. studied eight Hox proteins in Drosophila; their analysis was not as large-scale as the current study.

Yang et al.’s dataset [35] covers several TF families including homeodomain, C2H2, ETS, bZIP, bHLH, and Forkhead. However, the dataset mainly covers homeodomains (95/169 TFs). We found that 13 of the 19 TFs discussed above are homeodomains. These 19 TFs also include bHLH, bZIP, ETS, and C2H2 TFs, but the low presence of these other families could stem from this dataset’s non-uniform coverage. Forkhead TFs have been discussed by Bulyk and colleagues [20, 28] for their ability to recognize multiple motifs. We found only two of nine Forkhead TFs in this dataset have a non-canonical motif, and those too belong to the cases where the non-canonical motif appears to be similar across several different families (Supplementary Text 3, Table 1, Supplementary Table 1). It is also worth mentioning that we did not find a non-canonical motif for any of the 14 nuclear receptor factor TFs, suggesting that some TF families may have a characteristic lack of non-canonical motifs.

How can these non-canonical motifs be important if they are generally less abundant than canonical motifs? First, as we have shown, these motifs can explain many in vivo TF-occupied regions where the TF’s canonical motifs are absent. Secondly, in a tissue and cell-type specific manner, the occurrences of some of these non-canonical motifs show as strong an evolutionary conservation as the corresponding CIS-BP motifs. Finally, some non-canonical motifs also explain the TF’s occupancy in regulatory sequences. Altogether, we think non-canonical motifs can play functional roles in vivo, and hence, are important in order to gain a comprehensive understanding of a TF’s functional role. Non-canonical motifs are important also from the perspective of biochemical mechanisms. A recent study on HOXB13 and CDX2 [18] have shown that the two TFs recognize two similar sequences (“CAATAAA” and “TCGTAAA”), yet the recognition mechanisms are different in terms of thermodynamics. Such differences in recognition mechanisms may play a role in tissue and cell-type specificity of a TF. As such, it is important to consider the entire set of possible motifs for a TF.

## Methods

### Selection of HT-SELEX datasets and rounds

We analyzed the quality filtered datasets for 169 human TFs released with Yang et al.’s recent study [35]. Yang et al. resequenced these datasets from [14] at a significantly higher depth (on average ~10-fold increase in depth) and filtered through a quality-control pipeline [35]. For each dataset, we analyzed the same HT-SELEX round that Yang et al. selected based on a set of criteria that maximize the presence of both strong and weak sites for the corresponding TF.

### CIS-BP motifs and finding CIS-BP motif matches

As the motifs, we chose every CIS-BP motif [33] that was derived based on direct binding evidence. This returned a median number of four motifs per TF (range: 1−17). For detecting motif matches, we used the FIMO [10] program with the commonly adopted and relatively liberal significance threshold of 1e-4 [7, 12, 15, 16, 21, 31, 34].

### A pipeline to identify non-canonical motifs from HT-SELEX data

**Fig. 4:**
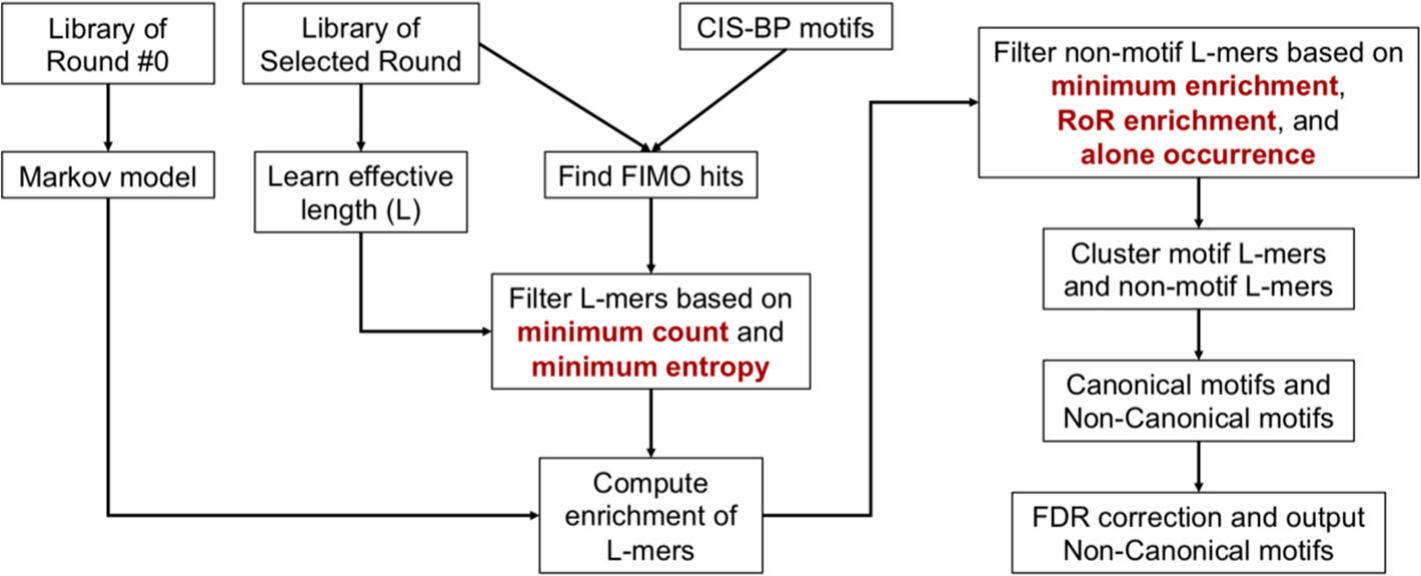
Flow diagram of the current analysis pipeline to identify non-canonical motifs. The pipeline combines the standard practices of HT-SELEX data modeling [29] with an additional set of conservative filtering criteria to eliminate experimental and statistical artifacts (shown in red). These criteria include selecting L-mers (sequences of length L) with a minimum count and a minimum di-nucleotide entropy, followed by selecting L-mers that have a minimum enrichment, positive enrichment over successive rounds, and alone occurrence (i.e., occurrence independent of the canonical motifs)

To investigate the existence of non-canonical motifs and their difference from canonical motifs, we developed a pipeline for analyzing HT-SELEX data. We combine the standard practices of HT-SELEX data modeling [29] with an additional set of conservative filtering criteria (Fig. 4). The additional criteria aim to ensure that any observed signal of non-canonical motifs is likely not an artifact of the HT-SELEX procedure [23]. The following is an outline of our pipeline; we describe the steps in detail in the following sub-sections.

Step 1. The HT-SELEX experiment for a TF starts with a library of random oligonucleotides (oligos), also known as the round #0 library. From the TF-bound oligos from the round #0 library, one then constructs the round #1 library and repeats this process for several rounds. Thus, each HT-SELEX round becomes more enriched for oligos with specific sites for the TF [14]. Here we analyze the HT-SELEX libraries that Yang et al. selected for each TF after applying a set of quality-control criteria [35]. These criteria were to ensure that the chosen rounds contained binding sites with an expected level of variation in the TFs’ DNA binding affinity.

Step 2. Within the oligos of the selected round, we identify the occurrences of the known motifs of the TF. As the known motifs, we use every CIS-BP motif [33] that is derived based on direct binding evidence in human. (In a post-analysis checking, we confirmed that the discovered non-canonical motifs do not match with CIS-BP motifs derived from indirect evidence.) We use the program FIMO [10] to identify the occurrences of the CIS-BP motifs in the oligos.

Step 3. Following the approach of Slattery et al. [29], we compute the effective length (*L*) of the TF’s binding sites. This is an information-theoretic approach to estimate the length of a TF’s DNA-binding sites without making assumptions about its DNA-binding properties.

Step 4. We perform an initial filtering on *L*-mers based on the following two criteria. First, an *L*-mer should occur at least 100 times (following [29]) in the selected round’s oligos. Secondly, we computed the entropy (based on dinucleotide frequency) of each *L*-mer, and discarded all *L*-mers that have an entropy lower than the minimum entropy of a CIS-BP motif occurrence.

Step 5. We compute the enrichment of the remaining *L*-mers in the selected round’s oligos with respect to round #0 oligos. To estimate the count of an *L*-mer in round #0, we build higher-order Markov models of the round #0 oligos following Slattery et al.’s approach [29].

Step 6. We then identify the “motif *L*-mers” and “non-motif *L*-mers”. An *L*-mer is a motif *L*-mer if it is a substring or a superstring of a CIS-BP motif occurrence, otherwise it is a non-motif *L*-mer. We discarded all non-motif *L*-mers that fail to satisfy the following three criteria. First, we discard all non-motif *L*-mers that have an enrichment lower than the least enriched motif *L*-mer. Secondly, for each non-motif *L*-mer, we compute its “round-over-round enrichment”, i.e., the ratio of its enrichment in each pair of successive rounds. We discard a non-motif *L*-mer if its round-over-round enrichment for any pair of successive rounds is less than 1. Finally, we discard the non-motif *L*-mers that always co-occur with motif *L*-mers in the selected round’s oligos.

Step 7. We separately cluster the motif *L*-mers and the non-motif *L*-mers into canonical and non-canonical motifs. To ensure the reliability of the clustering algorithm, we confirm that the canonical motifs are similar to the CIS-BP motifs of the corresponding TF. We discard every non-canonical motif which does not occur alone in at least 5% of the selected round’s oligos. When counting the number of oligos where a non-canonical motif occurs alone, we also ensure that the same oligo is not counted more than once for different non-canonical motifs.

Step 8. For each canonical and non-canonical motif, we compute its similarity score with the CIS-BP motifs. Based on the similarity scores of the canonical motifs, we then compute the empirical one-tailed *p*-values for the similarity scores of the non-canonical motif. We called a non-canonical motif as significantly different from the CIS-BP motifs if this one-tailed *p*-value passed the threshold corresponding to 5% FDR (false discovery rate) correction [4].

### Modeling ***k***-mer frequencies in round #0 libraries

We followed Slattery et al.’s [29] Markov model based procedure to model *k*-mer frequencies in round #0 of the HT-SELEX datasets. For each round #0 library, we first shuffled the order of the sequences and partitioned those into two equal sized datasets for training and validation. We then computed the optimal order of a Markov model for that library by fitting Markov models of order between zero and an integer *M* on the training sequences, and comparing the model performance (coefficient of determination, *R*^2^) on the validation sequences. To determine *M*, we identified the largest value of *k* such that every *k*-mer “occurs” at least 100 times in the library, and we set *M* = *k* − 1. We say that a *k*-mer *occurs* in a DNA sequence if the sequence has a *k*-length substring (either in the forward or the reverse complement direction) exactly matching the *k*-mer. Across the datasets, the median value of *M* was 6 (range: 5−8), the median value of optimal orders was 5 (range: 4−7), and the median *R*^2^ values of the optimal models was 0.94 (range: 0.76−0.99).

### Computing the effective length (***L***) of TF binding sites

We followed Slattery et al.’s [29] procedure to identify the effective site length (*L*) of a TF from its selected round’s library sequences. According to Slattery et al., for the effective site length *L*, the distribution of *L*-mer frequencies in the selected round should be maximally distant from the distribution of *L*-mer frequencies in round #0. Thus, for each value of *k* between *m* + 1 and 15, where *m* is the optimal order of Markov model for the corresponding round #0 library, we computed the KL divergence *D*_*KL*_ between the distributions of *k*-mer frequencies in the selected round and round #0 (to compute the *k*-mer frequencies in round #0, we used the Markov models computed above). We then set *L* equal to the value of *k* for which the above KL divergence was the maximum. Like Slattery et al., we took the frequencies of all *k*-mers that occur at least 100 times in the selected round and considered all other *k*-mers as one combined group. Thus, we computed the above KL divergence *D*_*KL*_ as follows.

$${D}_{KL}={\sum}_{w\in {S}_{100}}\kern0.24em {P}_R(w)\mathit{\log}\frac{P_R(w)}{P_0(w)}+{Q}_R(w)\mathit{\log}\frac{Q_R(w)}{Q_0(w)},$$

where:

*S*_100 _is the set of all *k*-mers that occur at least 100 times in the selected round *R*,

*P*_*R*_(*w*) and *P*_0_(*w*) are the frequencies of the *k*-mer *w* in rounds #R and #0 respectively, and ${Q}_R(w)=1-{\sum}_{w\in {S}_{100}}\;{P}_R(w)$ and ${Q}_0(w)=1-{\sum}_{w\in {S}_{100}}\kern0.24em {P}_0(w)$.

### Initial filtering of ***L***-mers from the library of the selected round

From the sequences (oligos) of the selected round’s library, we removed every *L*-mer if it failed in either of the following two criteria.

1. Minimum count: we eliminate an *L*-mer if it occurs less than 100 times in the library sequences
2. Minimum di-nucleotide based entropy: we first computed the minimum di-nucleotide based entropy of all matches to the TF’s CIS-BP motif, and we eliminate an *L*-mer if its di-nucleotide based entropy is less than the minimum of that computed from the CIS-BP motifs. We defined the di-nucleotide based entropy for a given sequence as follows.

$$H={\sum}_{k\in {S}_2}\;{p}_k\mathit{\log}\left(\frac{1}{p_k}\right),$$

where:

*S*_2 _is the set of all di-nucleotides, i.e., {AA, AC, AG, AT, …, TG, TT}, and

*p*_*k *_is the frequency of the *k*-th di-nucleotide in that sequence.

### Identifying motif ***L***-mers and non-motif ***L***-mers

We then rank all *L*-mers in the selected round’s library according to their enrichment with respect to round #0 library. We defined the enrichment of an *L*-mer *w* as $\frac{P_R(w)}{P_0(w)}$, where *P*_*R*_(*w*) and *P*_0_(*w*) are the frequencies of the *k*-mer *w* in rounds #R (the selected round) and #0 respectively.

We then mark every *L*-mer that is as either a substring or a superstring of the TF’s CIS-BP motif matches, and call these the “motif *L*-mers”. We identify the lowest ranked motif *L*-mer and discard every lower-ranked *L*-mers. From the remaining *L*-mers, we call an *L*-mer to be a “non-motif *L*-mer” if it is not a motif *L*-mer.

### Filtering non-motif ***L***-mers based on round-over-round (RoR) enrichment and alone occurrence

We further filtered non-motif *L*-mers based on the following two criteria.

1. Round-over-Round enrichment is at least one: for each non-motif *L*-mer, we computed its enrichment between every pair of consecutive rounds (Round-over-Round, RoR enrichment). We discarded a non-motif *L*-mer if its RoR enrichment between any pair of consecutive rounds was < 1.
2. Alone occurrence: we discard a non-motif *L*-mer if it never occurs “alone” in the selected round’s sequences, i.e., if it occurs with some motif *L*-mer in all oligos.

### Clustering ***L***-mers into canonical and non-canonical motifs

We take the filtered lists of motif *L*-mers and non-motif *L*-mers, and cluster the *L*-mers into canonical and non-canonical motifs. The key idea is to iteratively identify a cluster head (defined below) and cluster all the *L*-mers that: (a) have not been assigned to any other cluster yet and (b) are covered by the current cluster head (defined below).

A cluster head is an *l*-mer (we chose *l* = 8; if *L* < 8, then we chose *l* = *L* − 2) that occurs in the maximum number of *L*-mers with up to *m* = 2 mismatches (we use *m* = 1 if *l* ≤ 5). These choices were adopted from previous string-kernel based support vector machine models of TF binding specificity [2]. We say that a cluster head covers an *L*-mer if it occurs in the *L*-mer with up to *m* mismatches. Intuitively, a cluster head identifies a core region within the *L*-mers that it covers. After we cluster the *L*-mers covered by the current cluster head, we identify a new cluster head for the remaining *L*-mers and repeat the same process. We continue this iterative process until every *L*-mer has been assigned to a cluster or we have identified a maximum number of clusters (we set the limit at five).

We next align the *L*-mers in every cluster. We identify the position within each *L*-mer where the cluster head occurs with the fewest number of mismatches. We call these positions the anchor positions for alignment. If there are more than one anchor position for an *L*-mer, we choose the one that is closest to the middle position of the *L*-mer. We then align the *L*-mers along the anchor positions, and pad each *L*-mer with ‘N’s to make sure that all *L*-mers in the alignment have the same length. Of note, we always count mismatches by considering *l*-mers in both the forward and the reverse complement orientation.

From these alignments, we finally create the position weight matrices or motifs by counting the number of occurrences of each nucleotide at each position of the alignment. An ‘N’ at a position of an *L*-mer contributes a count of 0.25 to each nucleotide at that position.

We visually confirmed each canonical motif constructed from the above process and confirmed their similarity with the CIS-BP motifs of the same TF (Supplementary Table 1).

It is useful here to mention a final point about the non-canonical motifs constructed in the above process. At any stage during cluster construction, if we find multiple cluster heads (i.e., each of them covers the same number of *L*-mers), then we execute the above process independently for each cluster head. In such cases, the same *L*-mer will be assigned to more than one cluster and thus, will contribute to more than one motif. It is not clear how this may influence our downstream analyses. Therefore, after performing multiple test corrections on the non-canonical motifs (see below), we manually check if there is any pair of significant non-canonical motifs that includes the same *L*-mer and keep the motif that is more different from the CIS-BP motifs (see below). Thus, in our results, an *L*-mer never occurs more than once in the non-canonical motifs.

### Selecting non-canonical motifs based on fraction of Oligos explained

We say that a motif (canonical or non-canonical) of a TF *occurs* in a sequence (an oligo or a ChIP-Seq peak) if any of its constituent *L*-mers occur in the sequence. When a non-canonical motif of a TF occurs in a sequence, but none of the canonical motifs of the TF occurs in that sequence, we say that the non-canonical motif *occurs alone* in that sequence. We eliminate a non-canonical motif if it does not occur alone in at least 5% of the selected round’s oligos.

### Statistical significance of non-canonical motifs

For each canonical and non-canonical motif of a TF, we first computed its minimum distance *D*_*min*_ from the collection of CIS-BP motifs of the same TF. To compute the distance *D* between two motifs, we first trim the motifs by eliminating non-informative positions (information content less than 0.25 bits) from the two ends. Then we consider the every possible *l*-length sub-motifs (see below) of the two trimmed motifs, compute their Euclidean distances normalized by *l*, and report the minimum of these normalized distances as *D*. We chose *l* = 8 or set *l *= the length of the smaller motif if its length is smaller than 8. As we did for cluster heads above, the *l*-length sub-motifs capture the similarity between the two motifs in a core region. While computing the Euclidean distances, we always consider one of the motifs in both forward and reverse complement orientation, and take the smaller of the two distances.

Next, we compute the statistical significance of the *D*_*min*_ value of each non-canonical motif by computing a *p*-value using a normal distribution with mean and variances computed from the *D*_*min*_ values of the canonical motifs. We report this *p*-value as the statistical significance of the non-canonical motif.

Finally, we reported the non-canonical motifs that pass a 5% false discovery rate threshold in Benjamini-Hochberg procedure [4].

### ChIP-Seq and regulatory sequence data

We collected the ChIP-Seq data from Cistrome DB [17, 38] (Batch download for Human_Factor) and regulatory sequence annotations based on ENCODE [6, 8] and Roadmap Epigenomics [27] from Cao et al.’s [5] recent study. For promoters, we downloaded from UCSC [11] the sequences 1000 bases upstream of annotated transcription starts of RefSeq genes with annotated 5′ UTRs.

### Computational validation: enrichment of non-canonical motifs in ChIP-seq data

We computed the enrichment of non-canonical and canonical motifs in Cistrome ChIP-seq data using the following three control data: (i) shuffled versions of L-mers constituting the motifs, (ii) dinucleotide shuffled versions of ChIP-peaks, and (iii) randomly selected genomic sequences matched for length, GC-content, and repeat content (using gkmSVM [9]).

For the first analysis, for a given motif and a ChIP-seq dataset, we define the enrichment *e*(*m*) of a motif *m* as follows.

$$e(m)=\frac{1}{\left|D\right|}\sum \limits_{d\in D}\;\frac{1}{\left|L\right|}\sum \limits_{l\in L}\kern0.24em \frac{n\left(l,d\right)}{n\left(l\hbox{'},d\right)}$$

where,

*D *is the set of ChIP-seq datasets of the corresponding TF,

*L* is the set of L-mers constituting the motif,

*l*′ is the shuffled sequence of a constituent L-mer *l* of the motif *m*,

*n*(*l*, *d*) and *n*(*l*′, *d*) are the number of times *l* and *l*′ occur in the ChIP-peaks of a dataset *d*, respectively.

In other words, for each motif, we first take the mean ratio of the number of times its constituent L-mers and their shuffled sequences occur in the ChIP-peaks (we considered a pseudocount of 1). Then, we take the mean of the above statistic over all datasets of the corresponding TF.

For the other two analyses, for a given motif and a ChIP-seq dataset, we define the enrichment *e*(*m*) of a motif *m* as follows.

$$e(m)=\frac{1}{\left|D\right|}\sum \limits_{d\in D}\;\frac{1}{\left|L\right|}\sum \limits_{l\in L}\;\frac{n\left(l,d\right)}{n\left(l,C\right)}$$

where,

*D *is the set of ChIP-seq datasets of the corresponding TF,

*L* is the set of L-mers constituting the motif,

*n*(*l*, *d*) and *n*(*l*, *C*) are the number of times an L-mer *l* occurs in the ChIP-peaks of a dataset *d* and the corresponding control dataset *C*, respectively.

In other words, for each motif, we first take the mean ratio of the number of times its constituent L-mers occur in the ChIP-peaks compared to the control sequences (we considered a pseudocount of 1). Then, we take the mean of the above statistic over all datasets of the corresponding TF.

We show the results in Supplementary Figure 1 and discuss in Supplementary Text 2. To make the comparisons clear between non-canonical and canonical motifs, we have plotted the *e*(*m*) value of each non-canonical motif against the mean *e*(*m*) value of all canonical motifs of that TF.

### Computational validation: checking for the existence of non-canonical motifs in a separate HT-SELEX data

As a second computational validation, we analyzed the HT-SELEX dataset of Yin et al. [36]. This dataset includes DNA-binding data of full-length TFs and extended DNA binding domains for 28 of the 31 TFs that have non-canonical motifs in the Yang et al. dataset [35]. For each non-canonical motif, we computed its constituent L-mers’ enrichments in the HT-SELEX round that Yin et al. used to derive motifs compared to the first round of that dataset. We eliminated all L-mers with enrichment less than 1, and applied our clustering algorithms discussed above. We then scrutinized the resulting motifs for similarity with the non-canonical motifs discovered from Yang et al. data [35].

### Evolutionary conservation analysis

For each TF, we compared the 7-way (human and six vertebrates) phyloP scores [24] between its canonical and non-canonical motif occurrences within its ChIP-Seq peaks (Cistrome datasets [17, 38]). For non-canonical motif occurrences, we count only the occurrences within peaks that lack occurrences of the TF’s canonical motifs. For each matching sequence in these two groups, we computed its mean phyloP score from the basewise scores and performed a two sample Kolmogorov-Smirnov test on the two groups. We then computed the fraction of datasets per non-canonical motif where the two groups do not have a significantly different level of phyloP scores (two sample KS test *p*-value > 0.01).

## Supplementary Information

**Additional file 1: Supplementary Table 1.** All non-canonical motifs, logo visualization, and the associated statistics.

**Additional file 2: Supplementary Table 2.** Numbers of Cistrome DB ChIP-Seq datasets where the non-canonical and the canonical motif occurrences show similar evolutionary conservation.

**Additional file 3: Supplementary Text 1.**

**Additional file 4: Supplementary Text 2.**

**Additional file 5: Supplementary Figure 1.** Scatterplots showing the enrichments of non-canonical motifs against enrichments of canonical motifs in ChIP-seq data using three different control sequences: (a) dinucleotide shuffled versions of ChIP-peaks, (b) randomly selected genomic sequences matched for length, GC-content, and repeat-content, and (c) shuffled sequences of L-mers constituting the non-canonical and the canonical motifs.

**Additional file 6: Supplementary Text 3.**
