# Supplementary material for: Systematic identification of non-canonical transcription factor motifs
Source: BMC Mol Cell Biol. 2021 Aug 31;22:44. doi: 10.1186/s12860-021-00382-6 (PMC8408965; doi:10.1186/s12860-021-00382-6)
Supplement: Supplementary file 1 — Additional file 1: Supplementary Table 1. All non-canonical motifs, logo visualization, and the associated statistics. [file 12860_2021_382_MOESM1_ESM.pdf]

SUPPLEMENTARY TABLE 1

CHUMPITAZ-DIAZ, SAMEE, POLLARD

Contents

1. HOXB2, motif .1.1.2.1.3.1 2
2. SHOX, motif .1.1.2.1 4
3. SHOX, motif .1.1.2.1.3.1 6
4. SHOX, motif .1.1.2.1.3.2 8
5. EVX2, motif .1.1.2.1.3.2 10
6. EVX2, motif .1.1.2.1.3.1 12
7. SHOX2, motif .1.1.2.1 14
8. RAXL1, motif .1.1 16
9. TCF4, motif .1.1 18
10. TCF4, motif .1.1.2.1 20
11. BSX, motif .1.1.2.1.3.1.4.1.5.1 22
12. DBP, motif .1.1.2.1 24
13. ONECUT1, motif .1.1 26
14. LBX2, motif .1.1.2.1.3.1 28
15. ETV4, motif .1.1.2.1.3.1 30
16. PAX7, motif .1.1 32
17. PAX7, motif .1.1.2.1.3.1 34
18. ONECUT3, motif .1.1.2.1.3.1 36
19. ZNF784, motif .1.1 38
20. ZBTB49, motif .1.1 40
21. PDX1, motif .1.1 42
22. CEBPG, motif .1.1.2.1.3.1 44
23. POU2F3, motif .1.1 46
24. NKX3-1, motif .1.1.2.1 48
25. HOXC10, motif .1.1 50
26. CEBPG, motif .1.2.2.1 52
27. NKX3-1, motif .1.1.2.1.3.1 54
28. NOTO, motif .1.1.2.1 56
29. NOTO, motif .1.1.2.1.3.1 58
30. ZNF740, motif .1.1 60
31. FOXI1, motif .1.1.2.1.3.1.4.1 62
32. ATF4, motif .1.1.2.1 64
33. ATF4, motif .1.1.2.1.3.1 66
34. ATF4, motif .1.1 68
35. NOTO, motif .1.1 70
36. SPDEF, motif .1.1 72
37. KLF16, motif .1.1.2.1 75
38. FOXI1, motif .1.1 77
39. NOTO, motif .1.1.2.1.3.1.4.1 79
40. POU3F2, motif .1.1 81
41. FOXB1, motif .1.1 83

Date: March 3, 2021.

1. HOXB2, MOTIF .1.1.2.1.3.1

Fractions of oligos explained (independently of CIS-BP motifs): 9.7458%
Distance: 0.175976804718
p-value: 0.000000

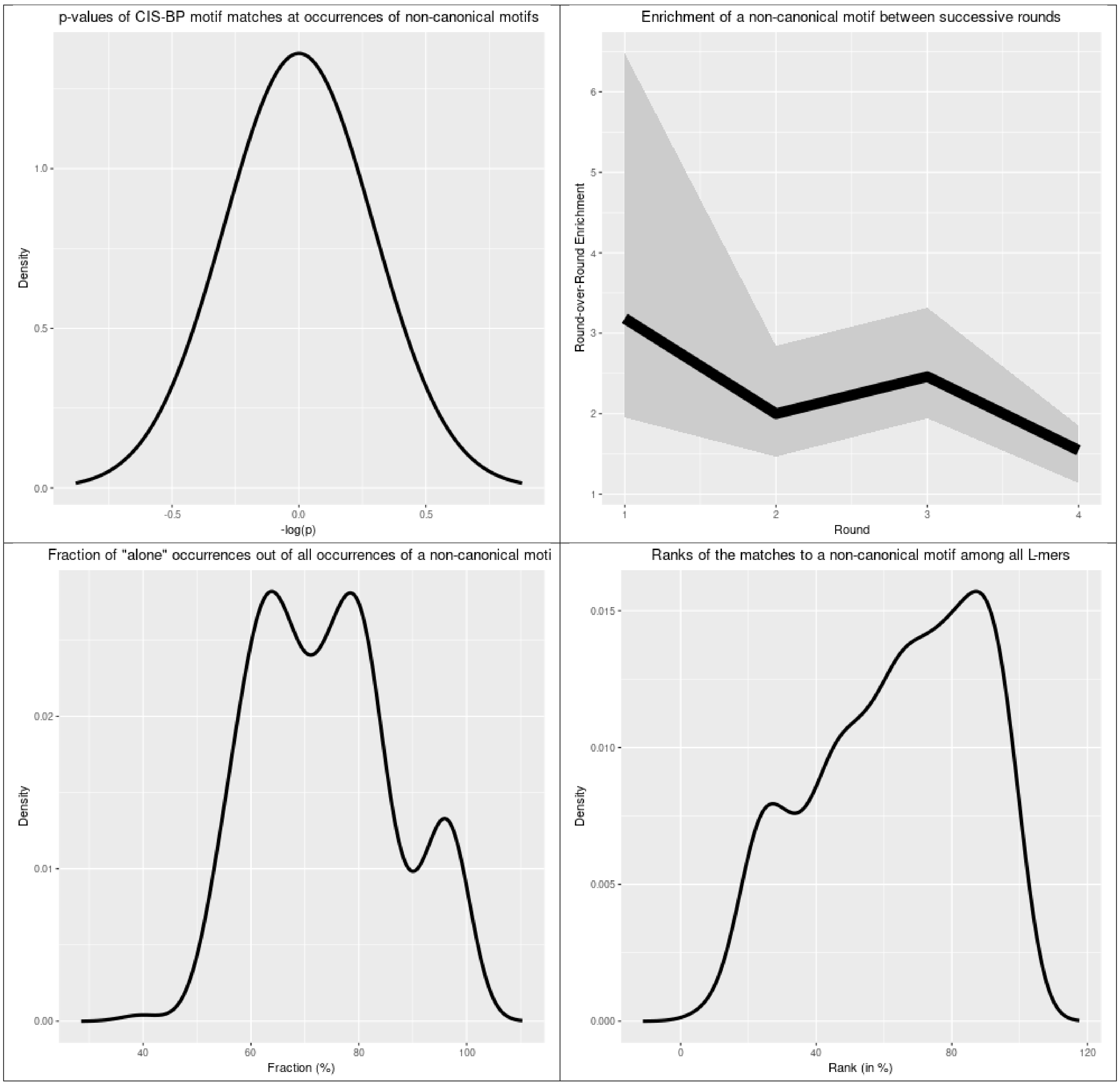

CIS-BP	Canonical	Non-canonical	Yin et al.
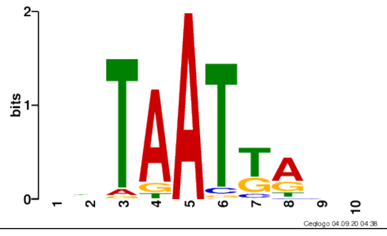	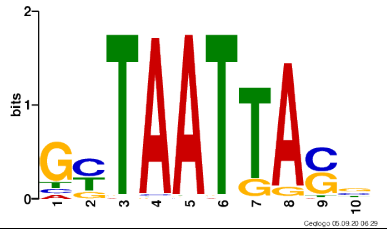	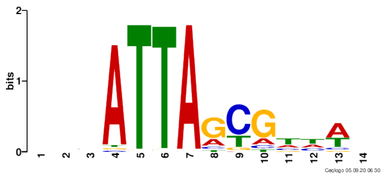	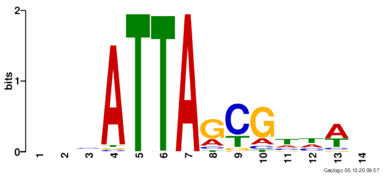
	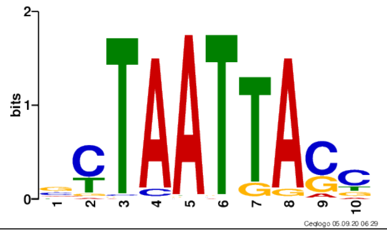		

Back to Table of Contents

2. SHOX, motif .1.1.2.1

Fractions of oligos explained (independently of CIS-BP motifs): 19.8060%
Distance: 0.0775156848142
p-value: 0.002078

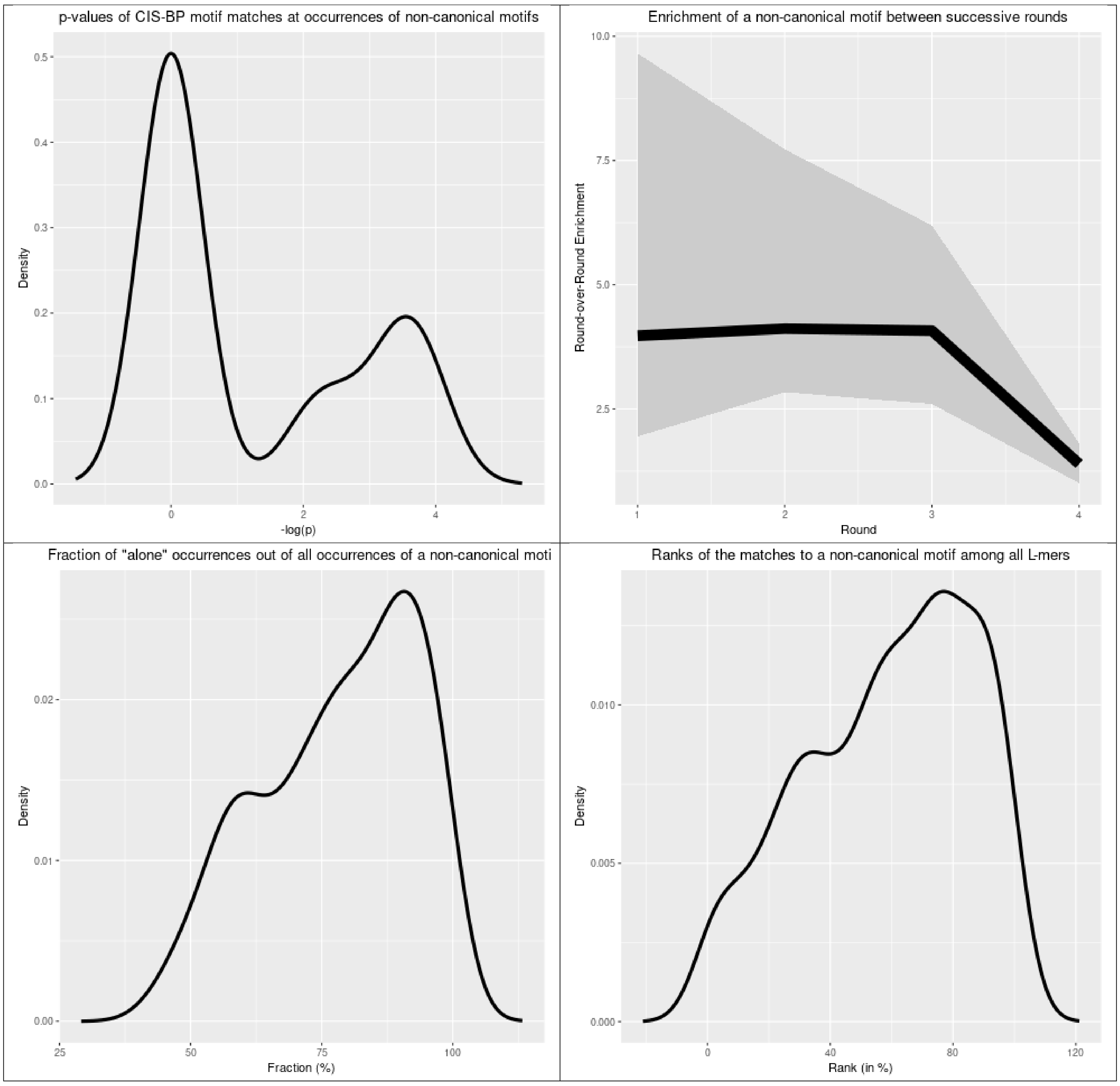

CIS-BP	Canonical	Non-canonical	Yin et al.
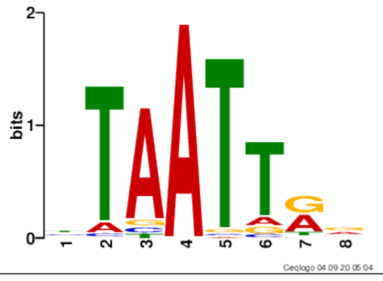	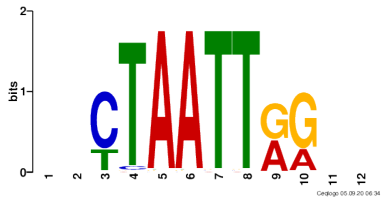	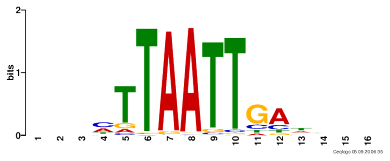	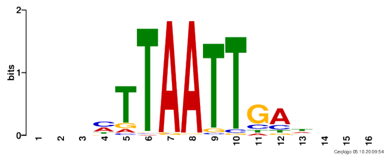
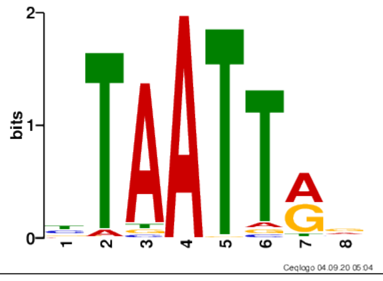	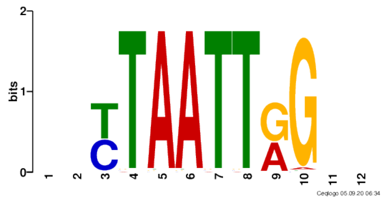		
	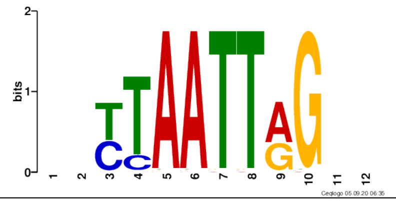		
	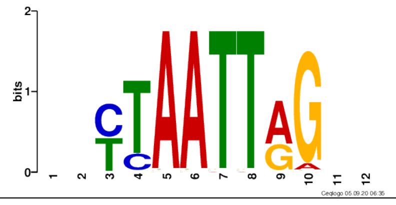		
	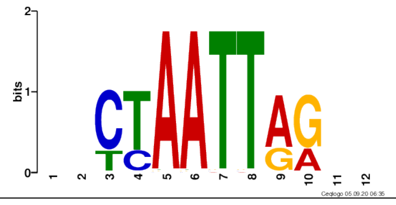		

Back to Table of Contents

3. SHOX, motif .1.1.2.1.3.1

Fractions of oligos explained (independently of CIS-BP motifs): 6.8528%
Distance: 0.174119759296
p-value: 0.000000

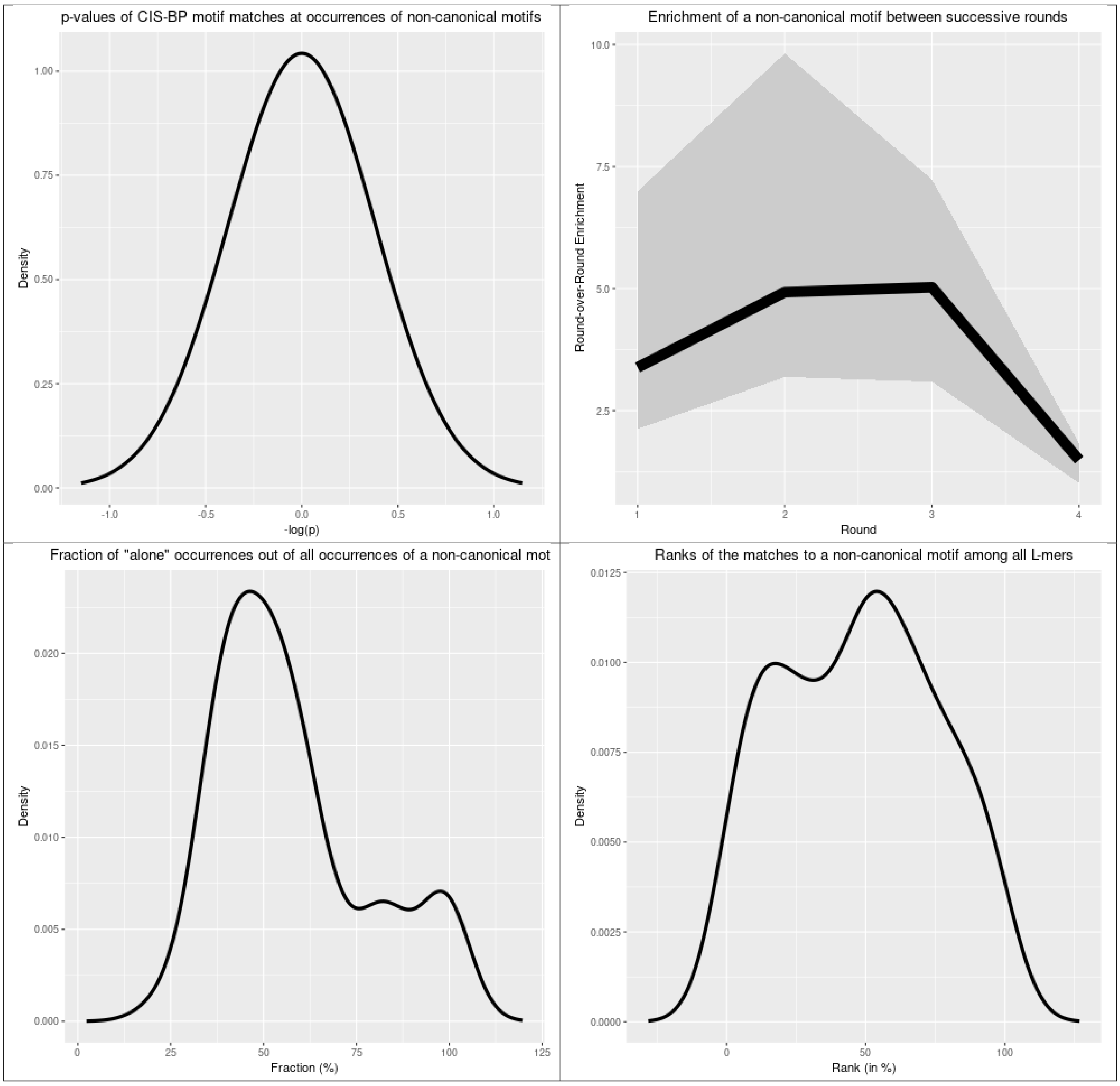

CIS-BP	Canonical	Non-canonical	Yin et al.
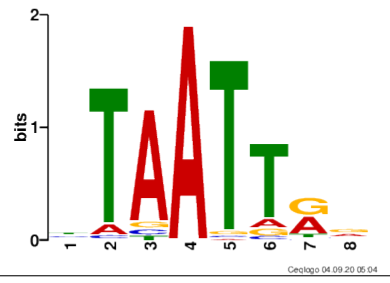	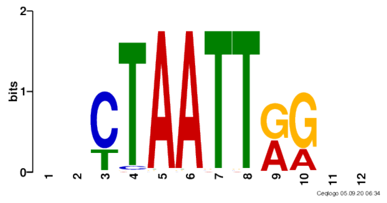	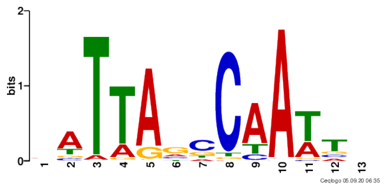	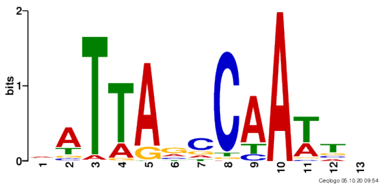
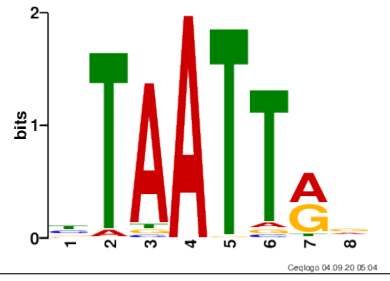	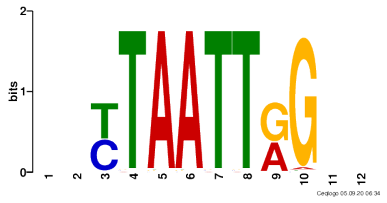		
	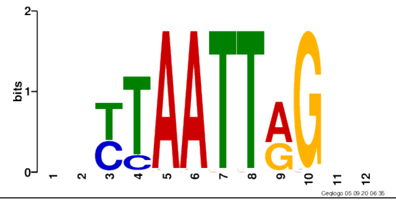		
	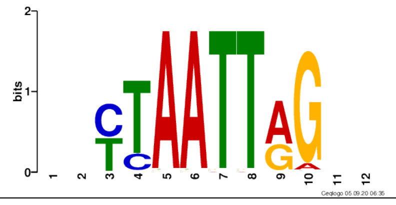		
	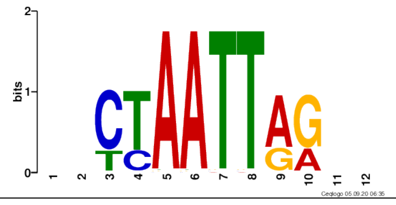		

Back to Table of Contents

4. SHOX, MOTIF .1.1.2.1.3.2

Fractions of oligos explained (independently of CIS-BP motifs): 7.2097%
Distance: 0.136678842816
p-value: 0.000000

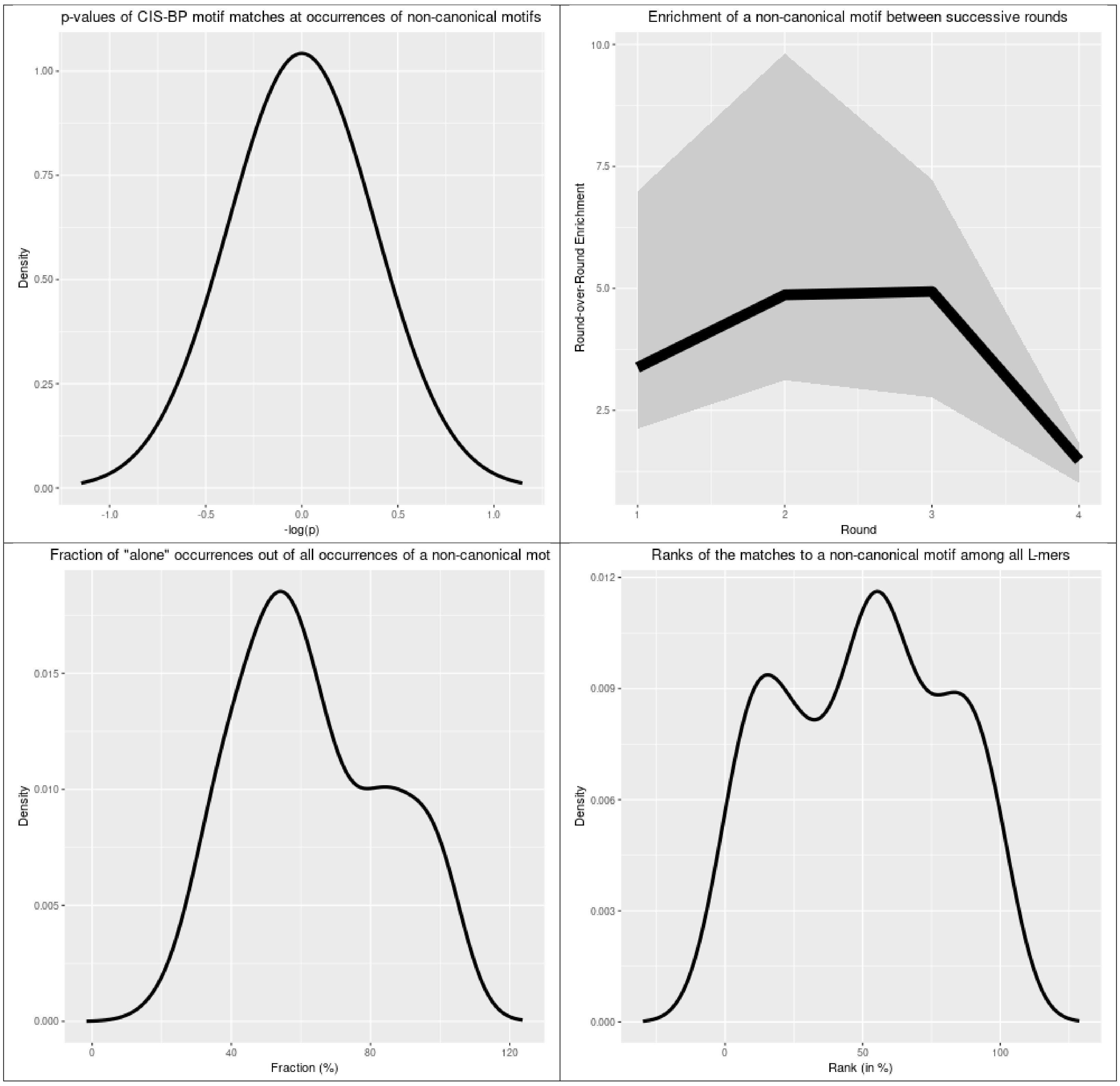

CIS-BP	Canonical	Non-canonical	Yin et al.
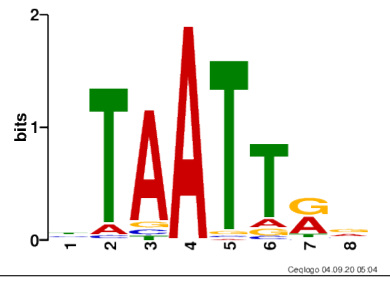	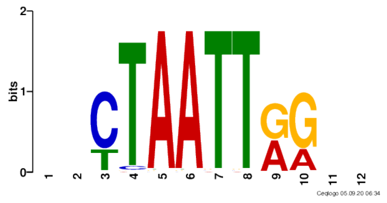	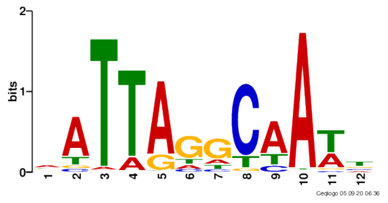	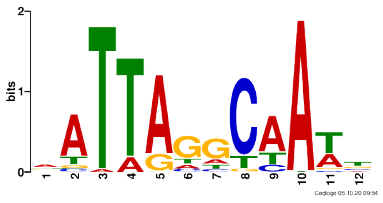
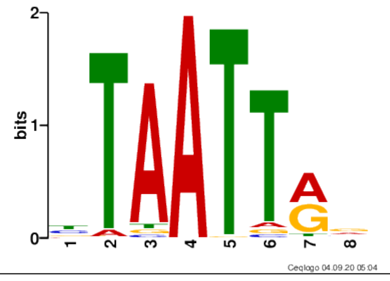	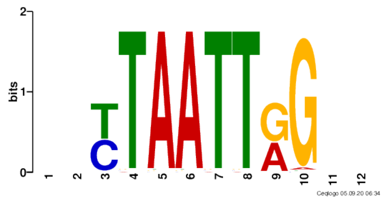		
	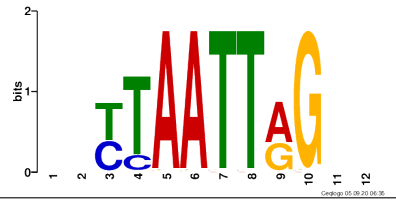		
	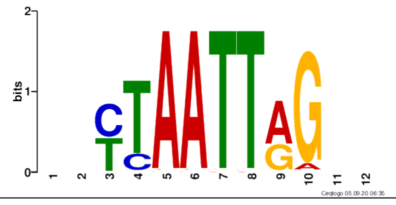		
	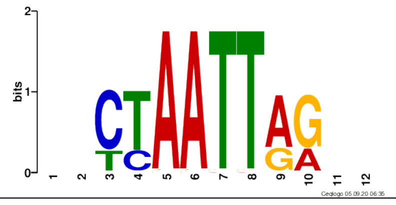		

Back to Table of Contents

5. EVX2, motif .1.1.2.1.3.2

Fractions of oligos explained (independently of CIS-BP motifs): 10.2403%
Distance: 0.1312144463452
p-value: 0.000000

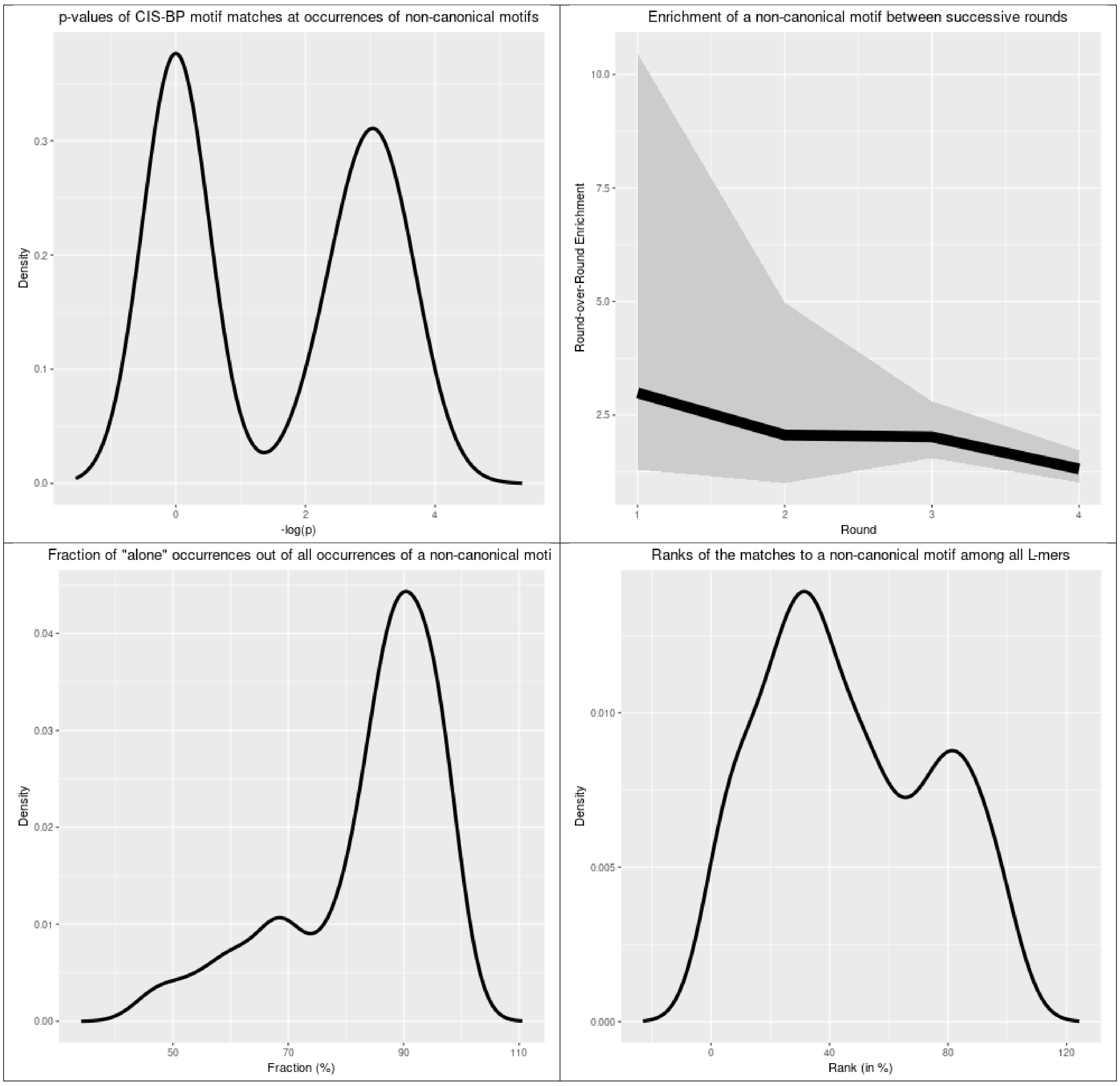

CIS-BP	Canonical	Non-canonical	Yin et al.
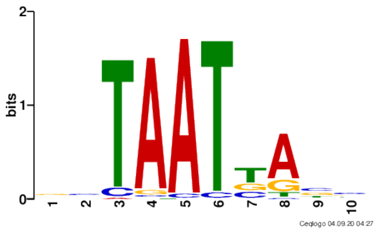	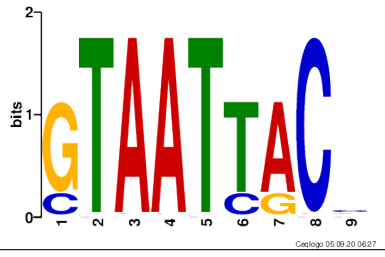	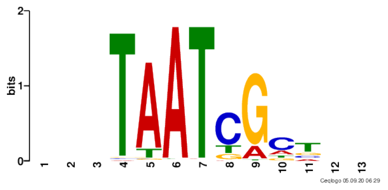	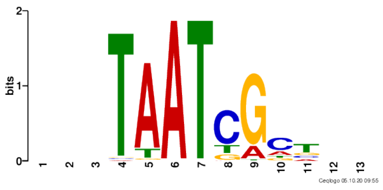
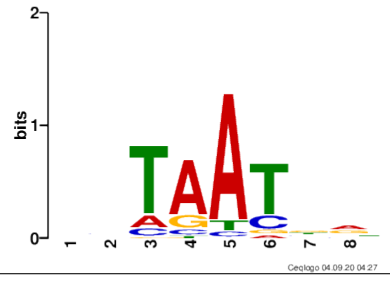	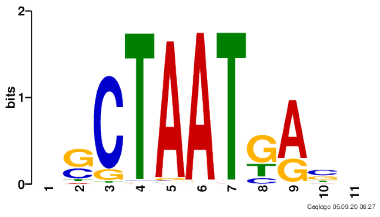		
	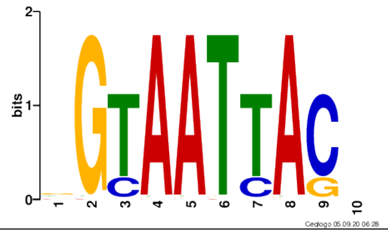		

Back to Table of Contents

6. EVX2, motif .1.1.2.1.3.1

Fractions of oligos explained (independently of CIS-BP motifs): 8.3259%
Distance: 0.139462503726
p-value: 0.000000

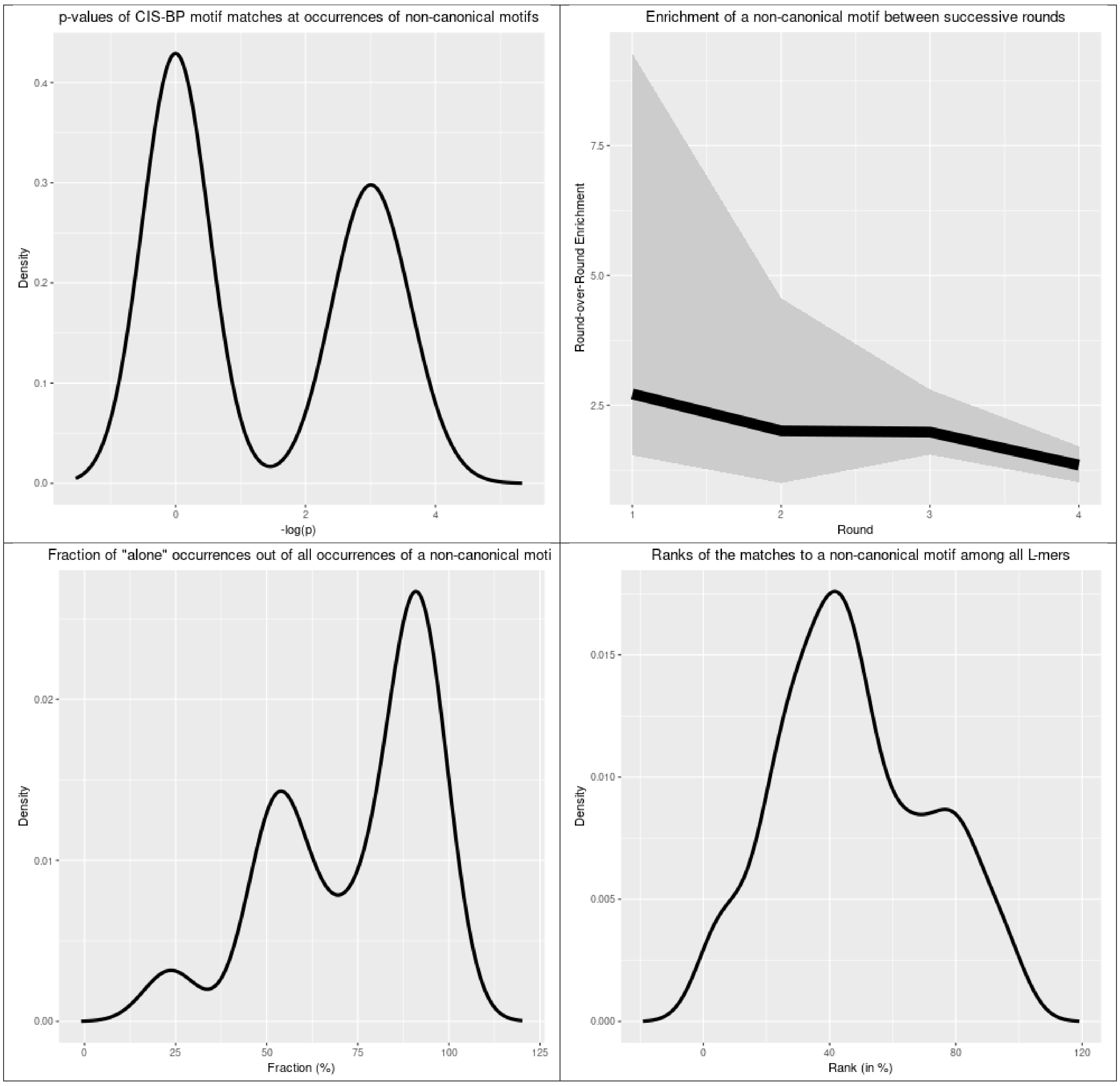

CIS-BP	Canonical	Non-canonical	Yin et al.
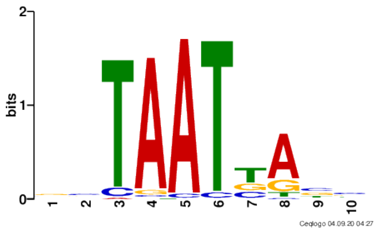	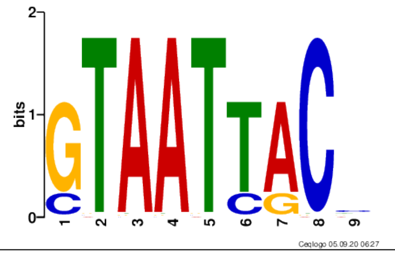	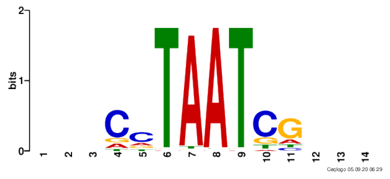	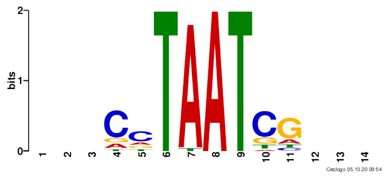
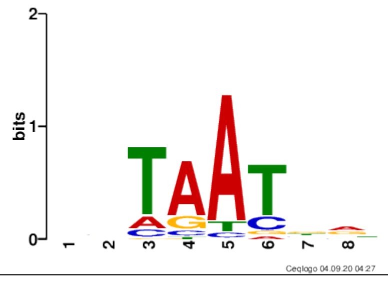	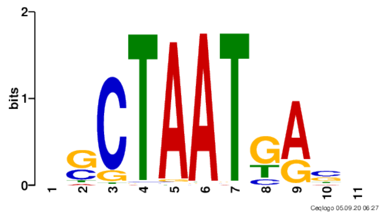		
	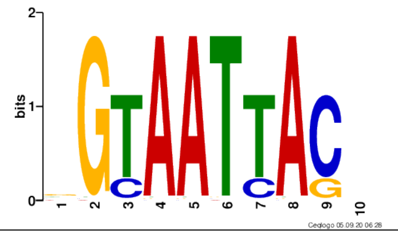		

Back to Table of Contents

7. SHOX2, motif .1.1.2.1

Fractions of oligos explained (independently of CIS-BP motifs): 16.0336%
Distance: 0.0844764470273
p-value: 0.000000

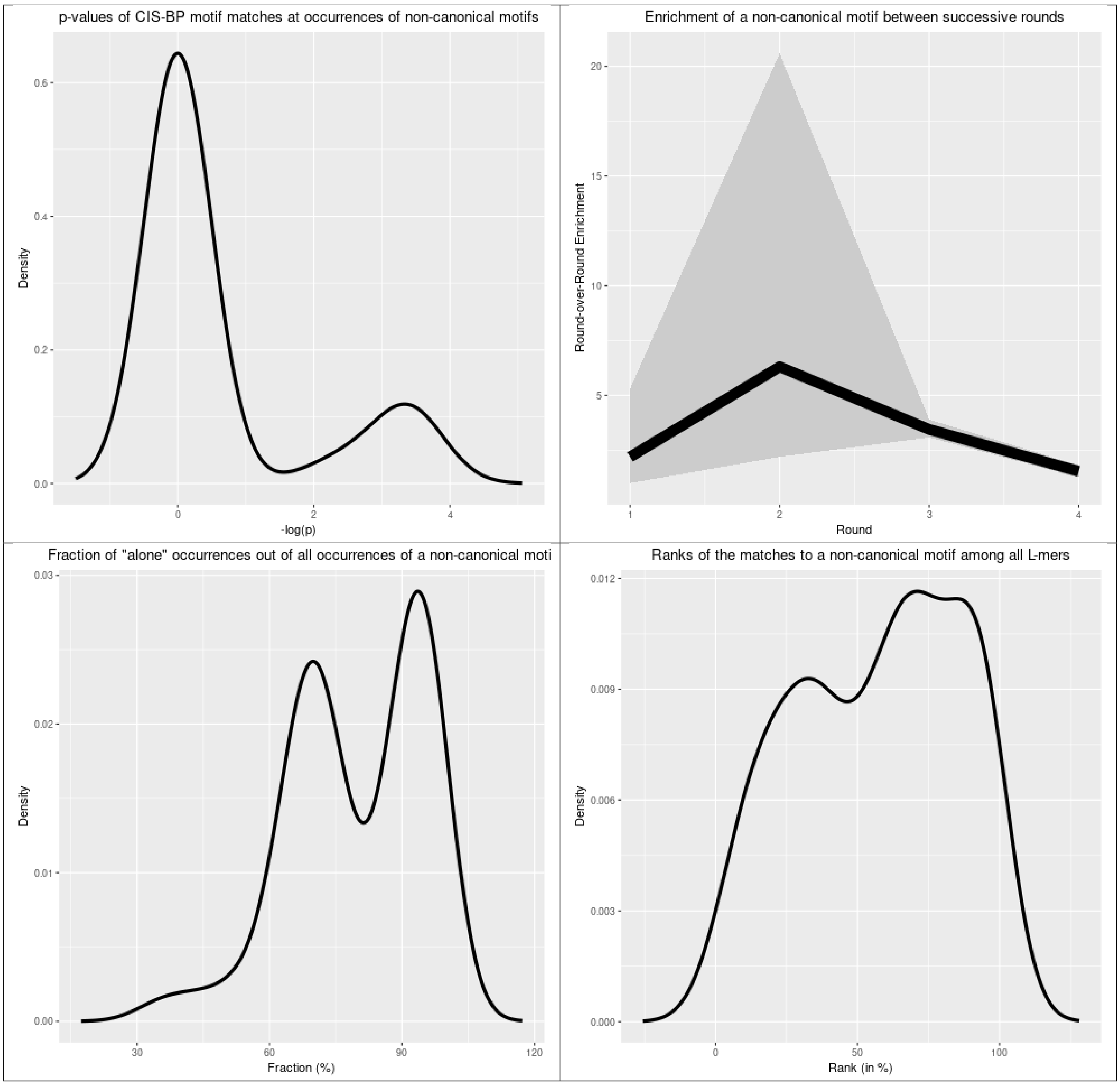

CIS-BP	Canonical	Non-canonical	Yin et al.
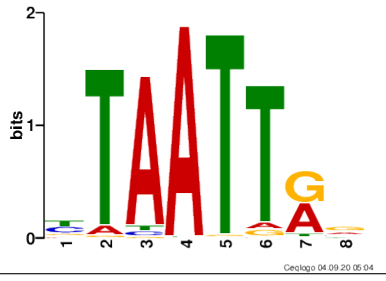	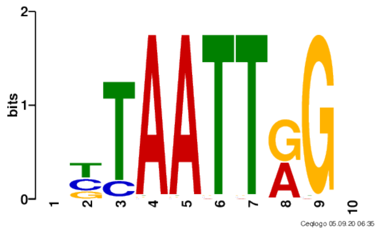	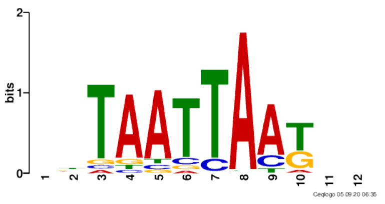	NA
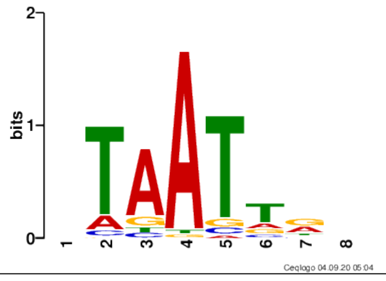	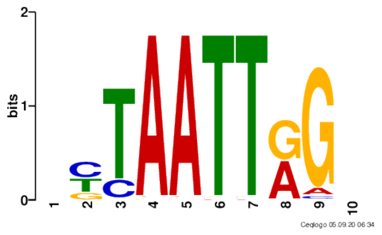		
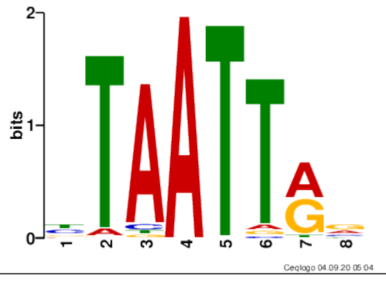			

Back to Table of Contents

8. RAXL1, motif .1.1

Fractions of oligos explained (independently of CIS-BP motifs): 22.3923%
Distance: 0.0879281013149
p-value: 0.000000

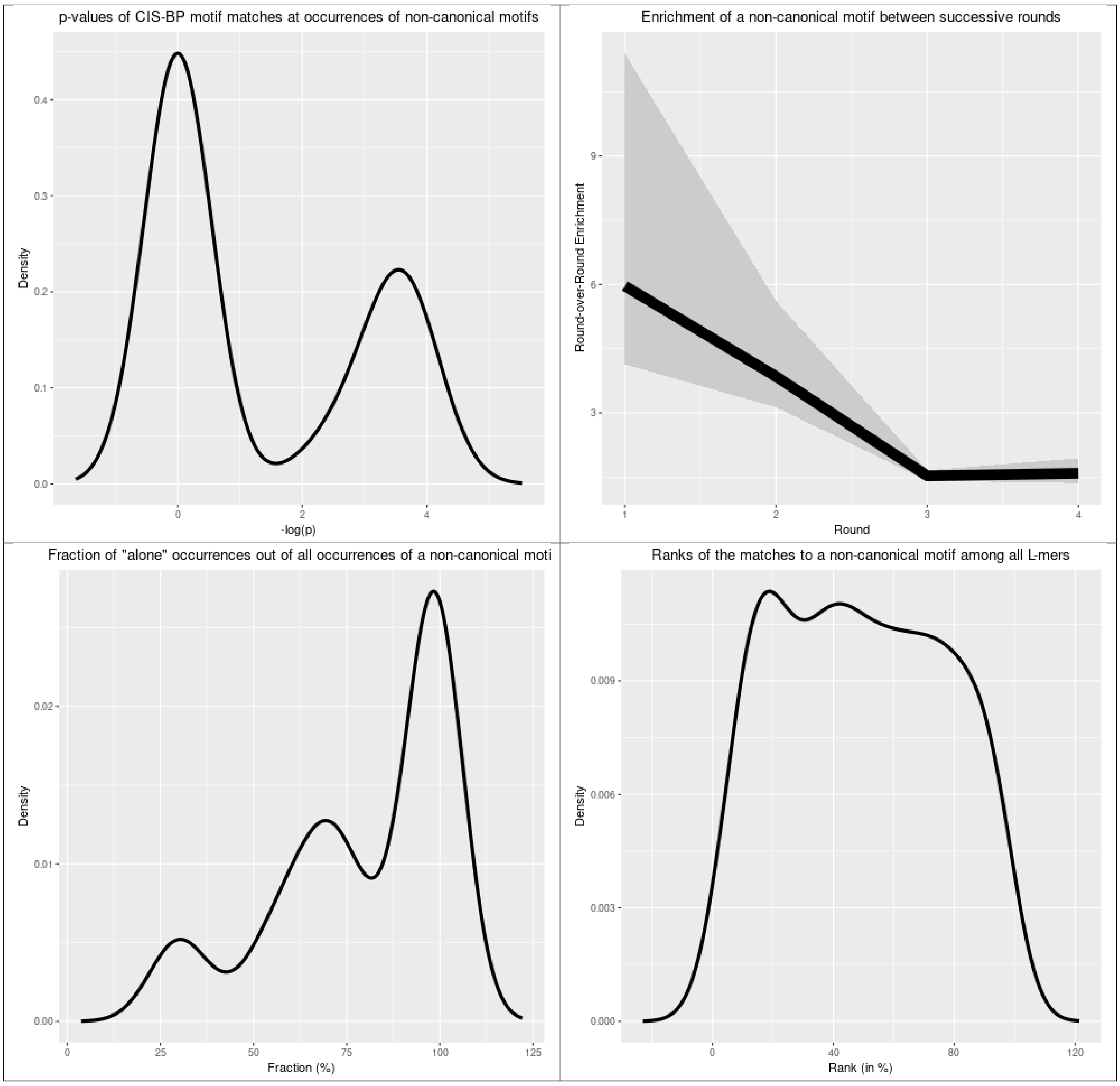

CIS-BP	Canonical	Non-canonical	Yin et al.
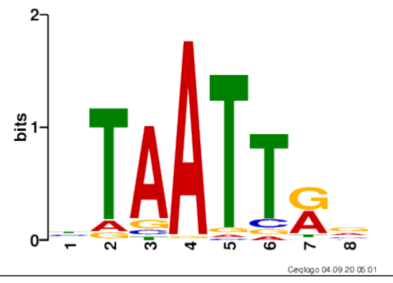	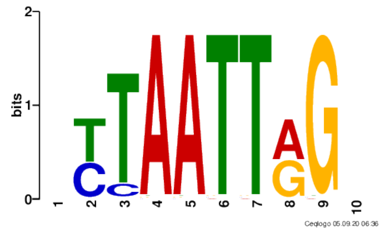	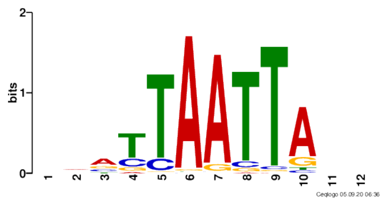	NA
	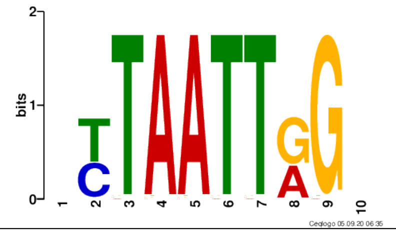		

Back to Table of Contents

9. TCF4, MOTIF .1.1

Fractions of oligos explained (independently of CIS-BP motifs): 22.0287%
Distance: 0.0613763144455
p-value: 0.000000

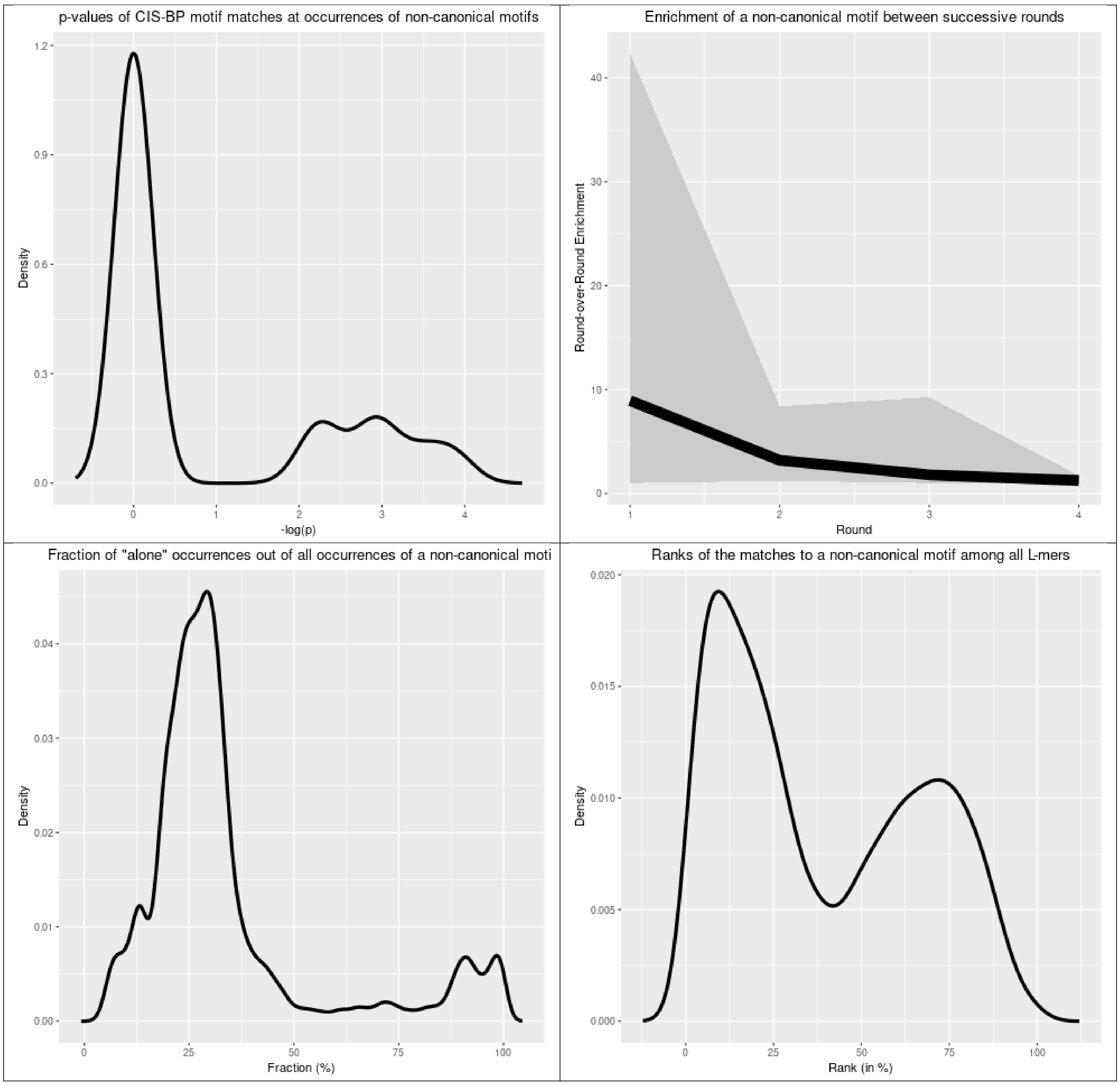

CIS-BP	Canonical	Non-canonical	Yin et al.
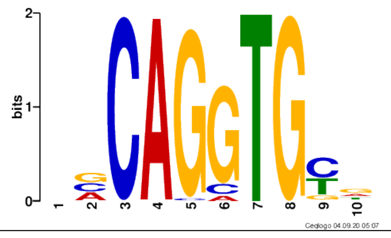	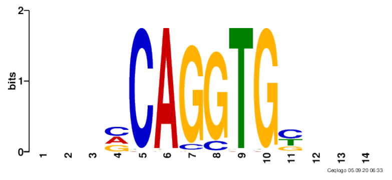	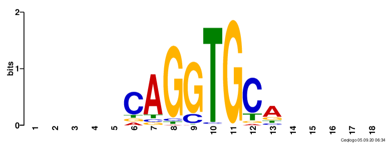	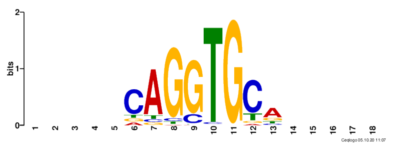
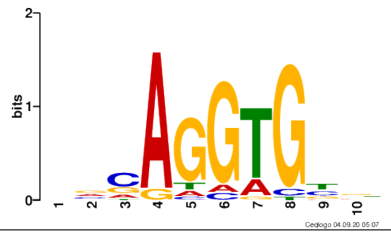	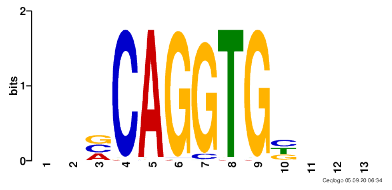		
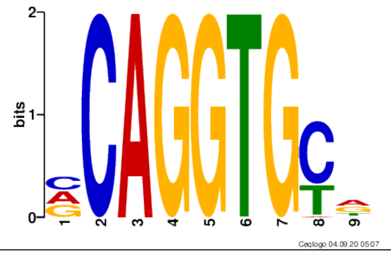	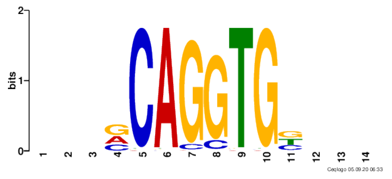		
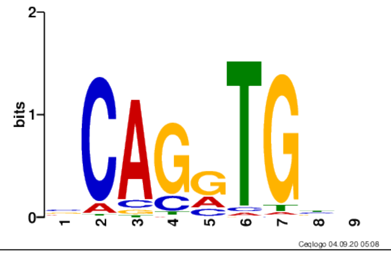			

Back to Table of Contents

10. TCF4, MOTIF .1.1.2.1

Fractions of oligos explained (independently of CIS-BP motifs): 7.6175%
Distance: 0.0752497297677
p-value: 0.000000

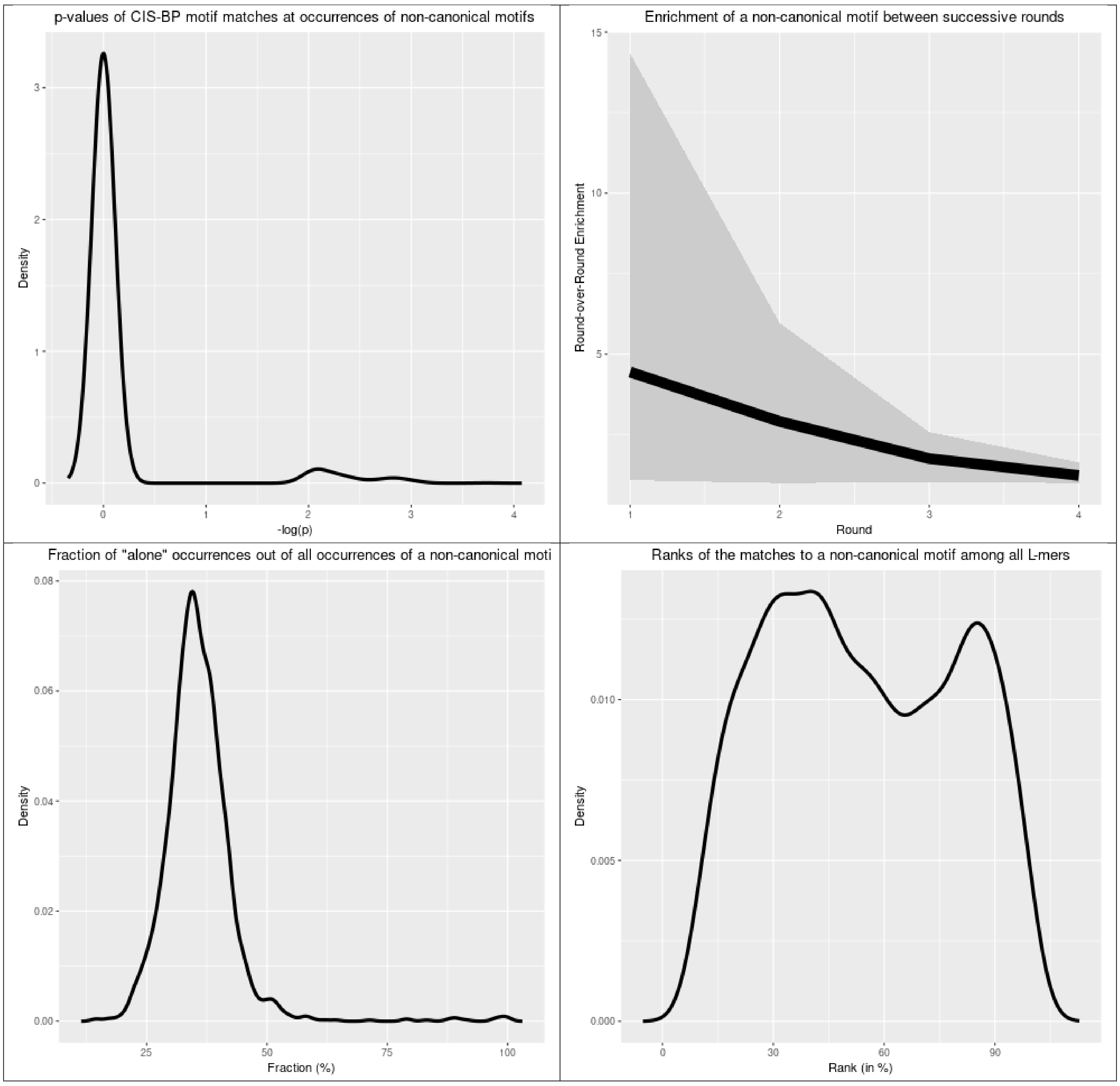

CIS-BP	Canonical	Non-canonical	Yin et al.
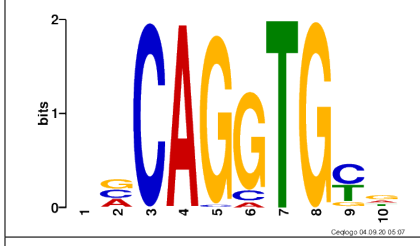	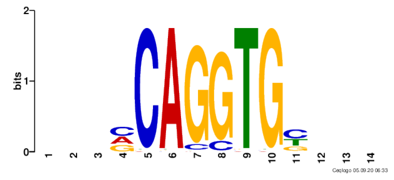	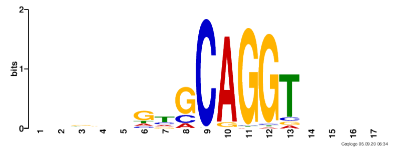	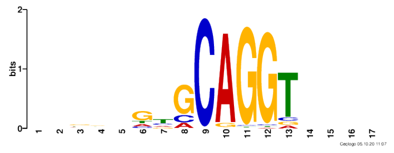
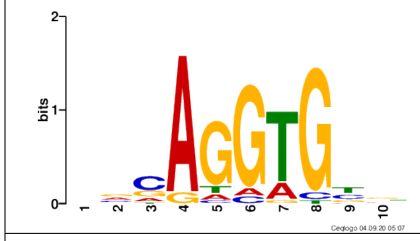	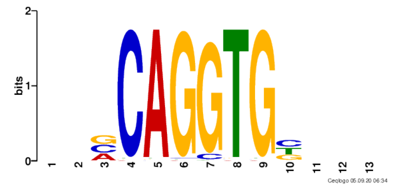		
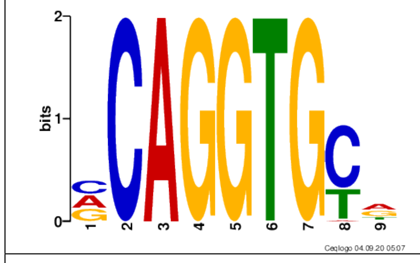	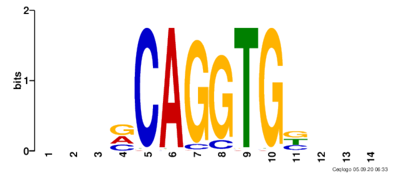		
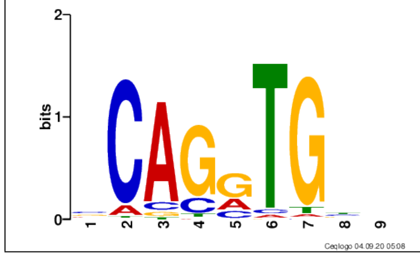			

Back to Table of Contents

11. BSX, MOTIF .1.1.2.1.3.1.4.1.5.1

Fractions of oligos explained (independently of CIS-BP motifs): 7.6262%
Distance: 0.199665188511
p-value: 0.000000

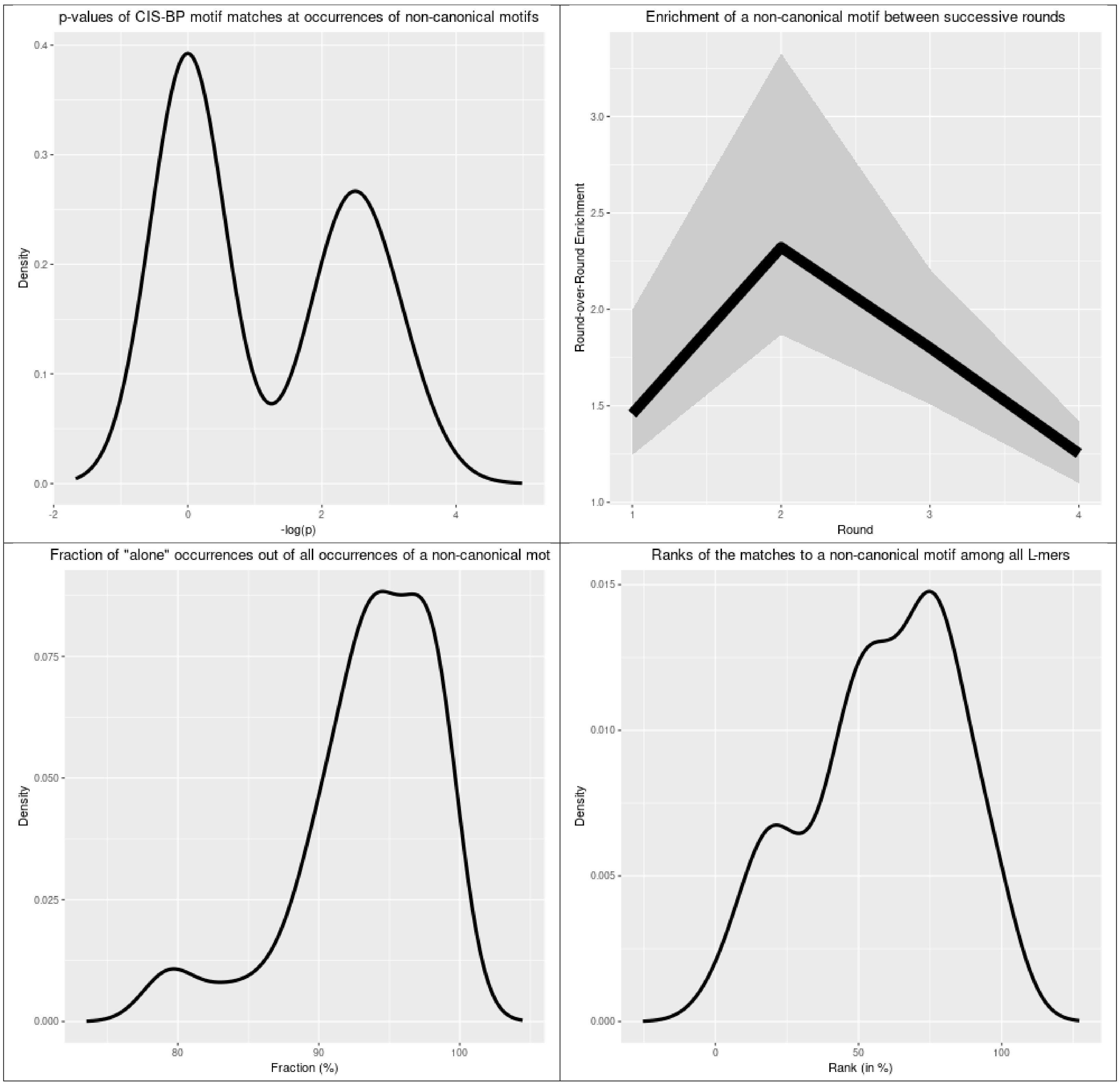

CIS-BP	Canonical	Non-canonical	Yin et al.
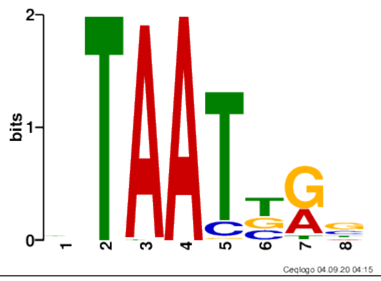	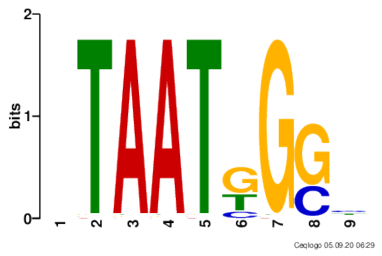	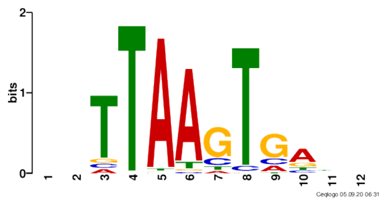	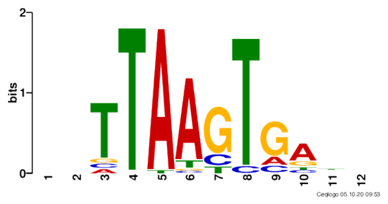
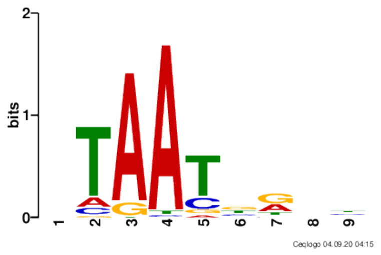	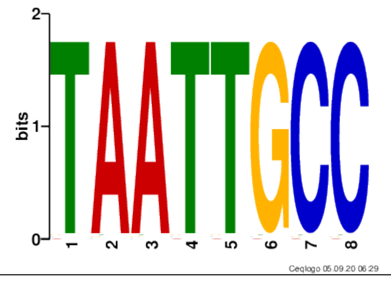		

Back to Table of Contents

12. DBP, motif .1.1.2.1

Fractions of oligos explained (independently of CIS-BP motifs): 5.4804%
Distance: 0.1041908889426
p-value: 0.000865

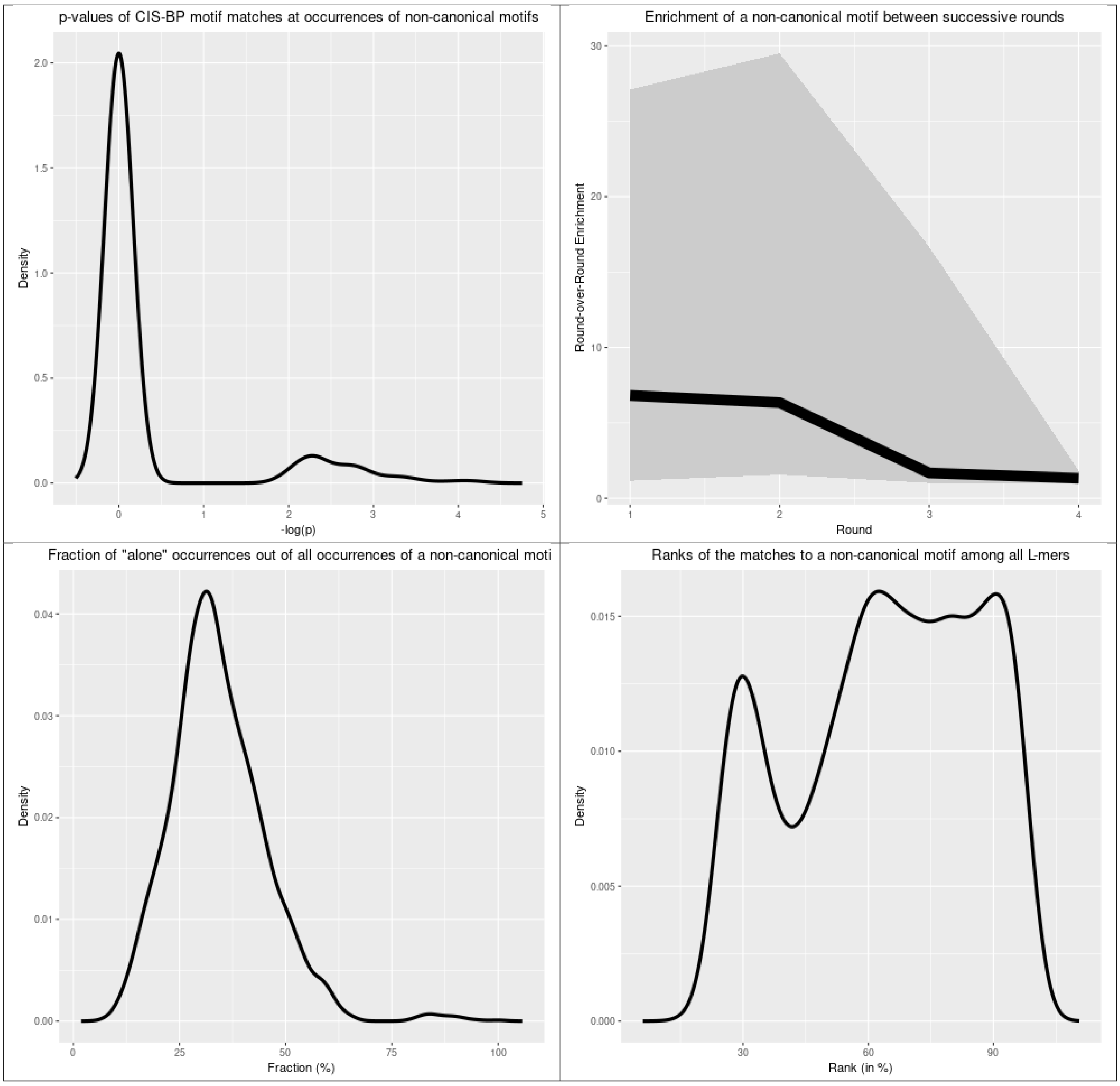

CIS-BP	Canonical	Non-canonical	Yin et al.
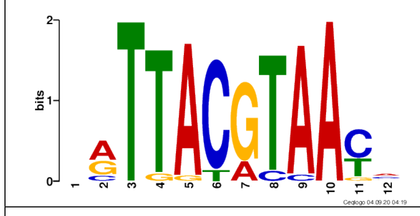	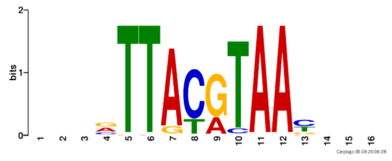	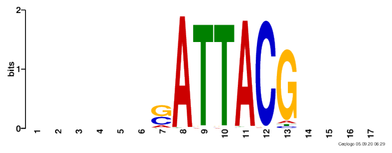	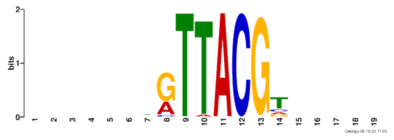
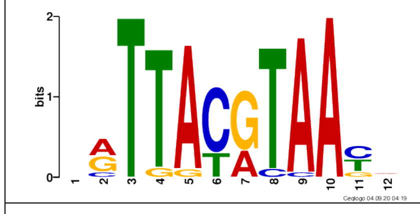	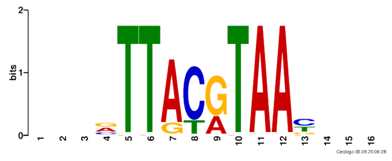		
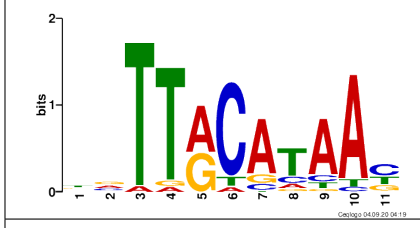	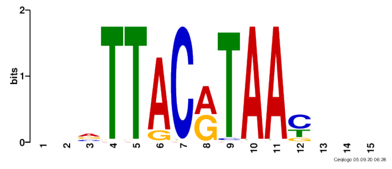		
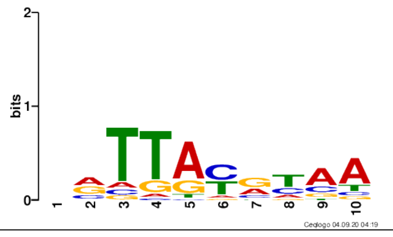	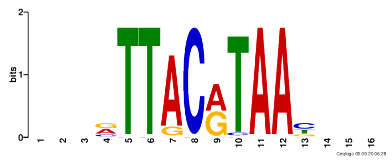		
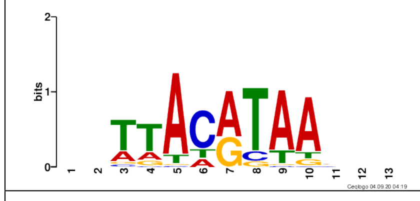			
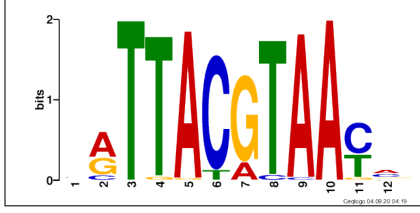			

Back to Table of Contents

13. ONECUT1, MOTIF .1.1

Fractions of oligos explained (independently of CIS-BP motifs): 16.6771%
Distance: 0.0693210852259
p-value: 0.000000

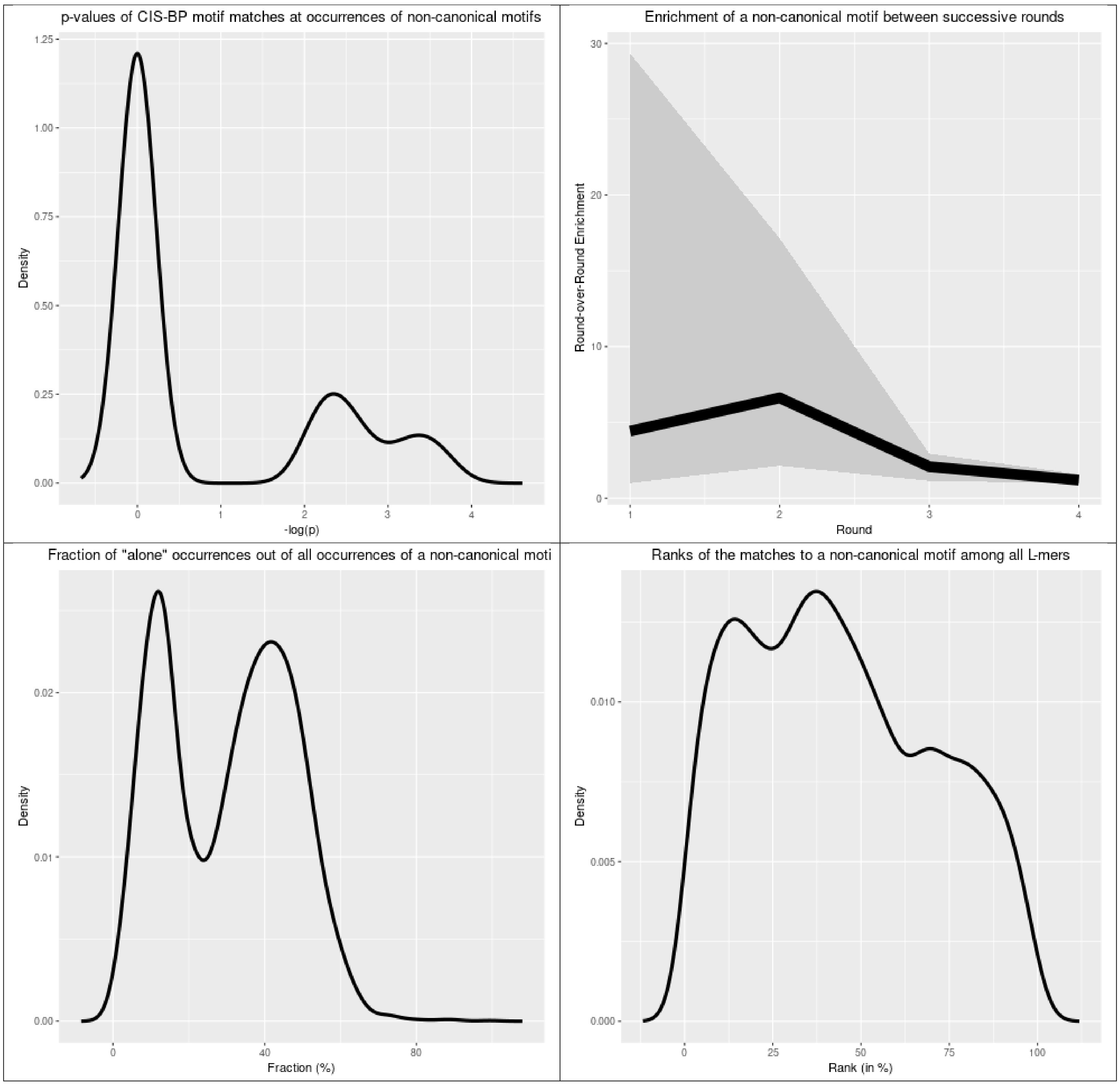

CIS-BP	Canonical	Non-canonical	Yin et al.
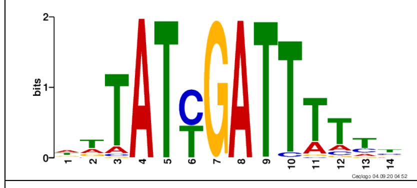	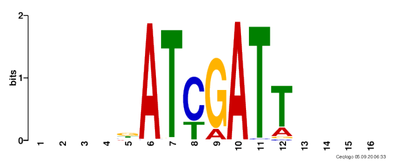	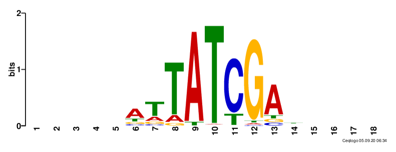	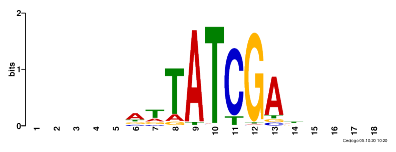
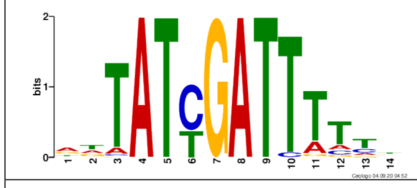	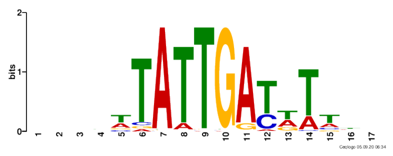		
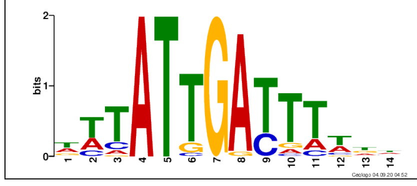			

Back to Table of Contents

14. LBX2, motif .1.1.2.1.3.1

Fractions of oligos explained (independently of CIS-BP motifs): 8.1947%
Distance: 0.183275635208
p-value: 0.001760

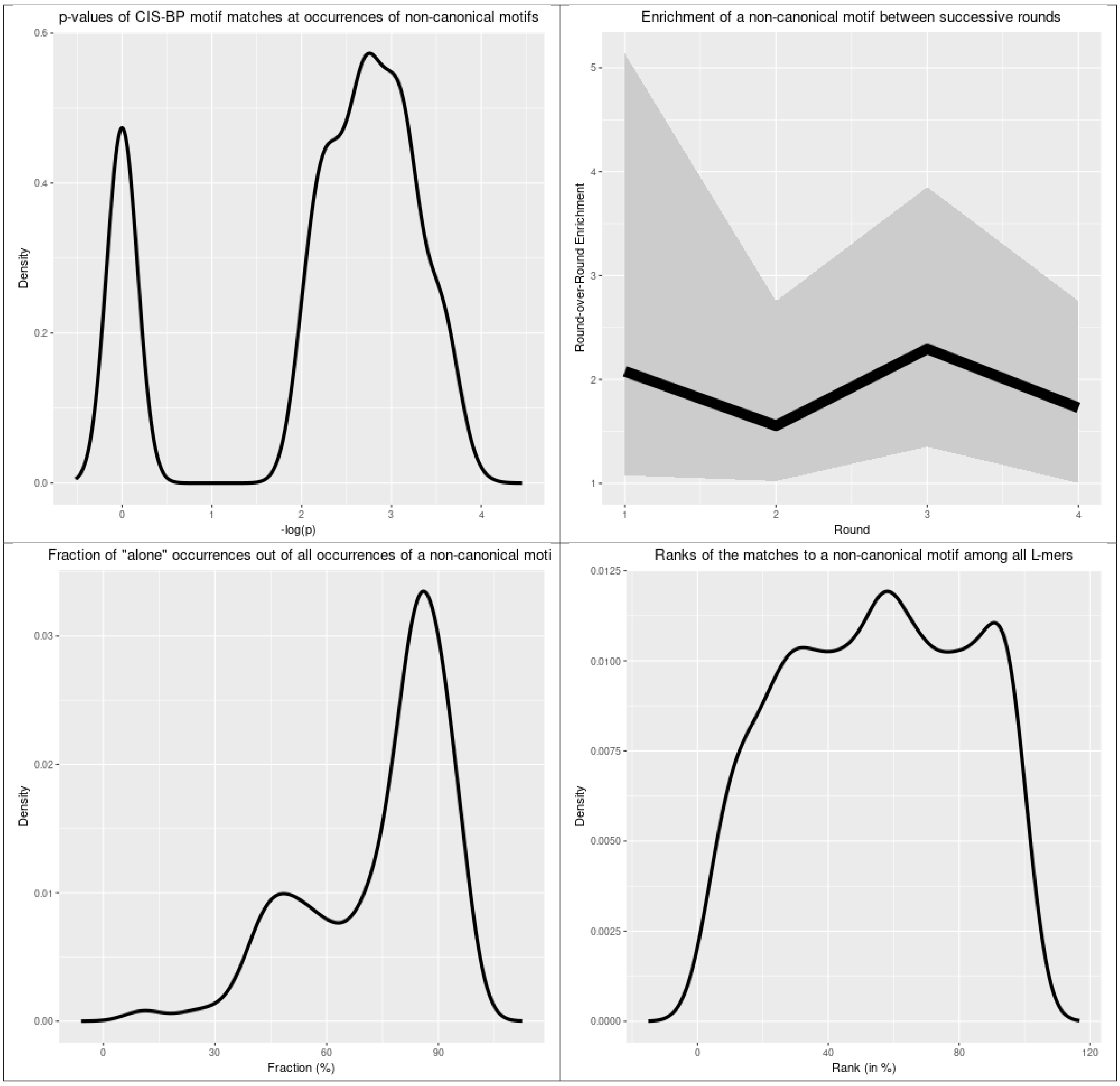

CIS-BP	Canonical	Non-canonical	Yin et al.
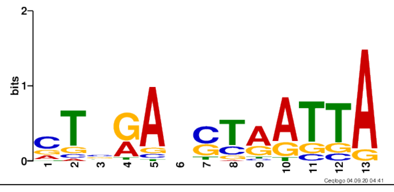	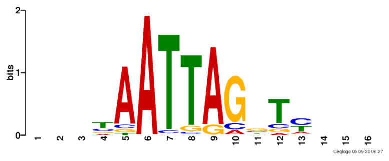	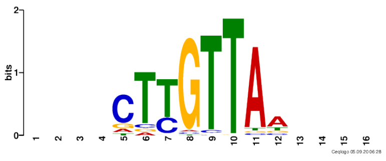	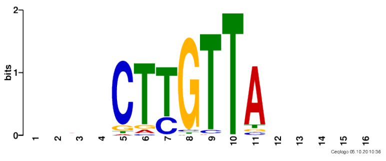
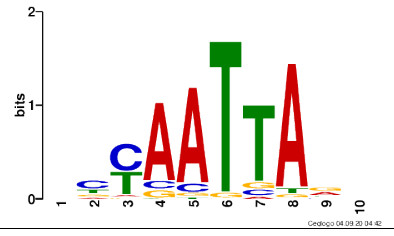	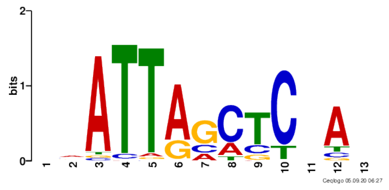		
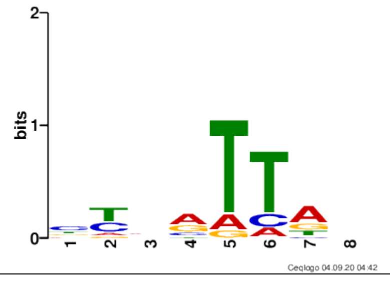	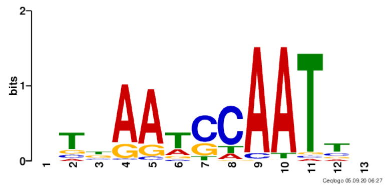		

Back to Table of Contents

15. ETV4, MOTIF .1.1.2.1.3.1

Fractions of oligos explained (independently of CIS-BP motifs): 5.7322%
Distance: 0.21973128756
p-value: 0.004996

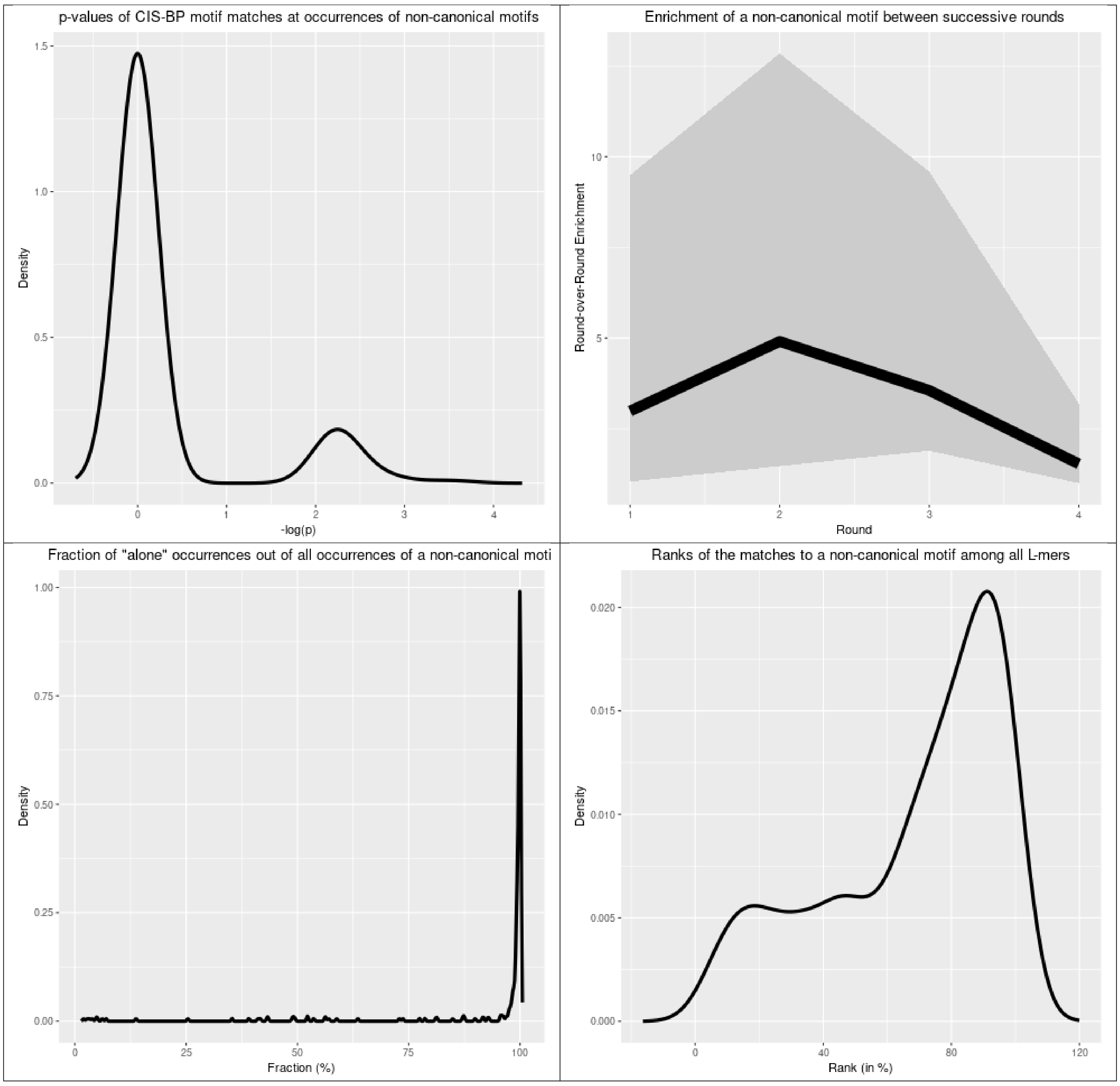

CIS-BP	Canonical	Non-canonical	Yin et al.
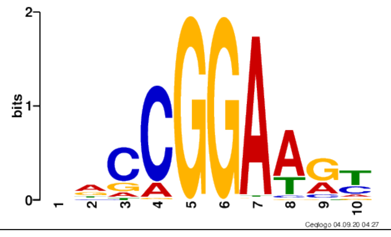	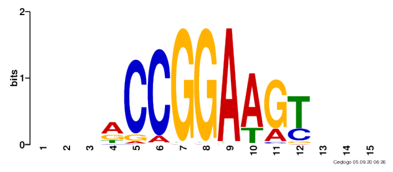	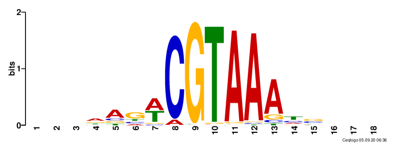	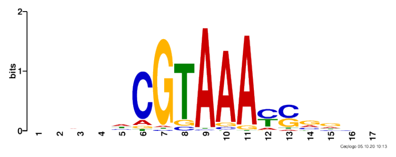
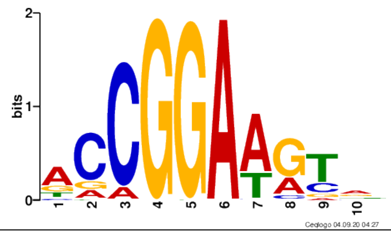	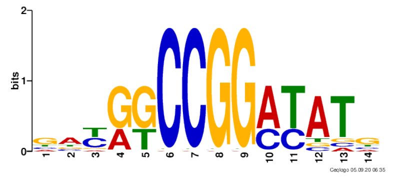		
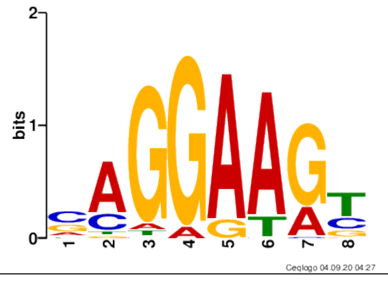	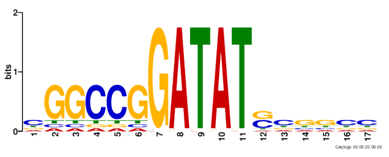		
	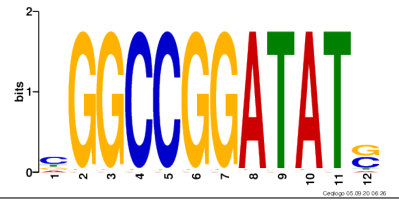		

Back to Table of Contents

16. PAX7, motif .1.1

Fractions of oligos explained (independently of CIS-BP motifs): 12.9464%
Distance: 0.204346152437
p-value: 0.000000

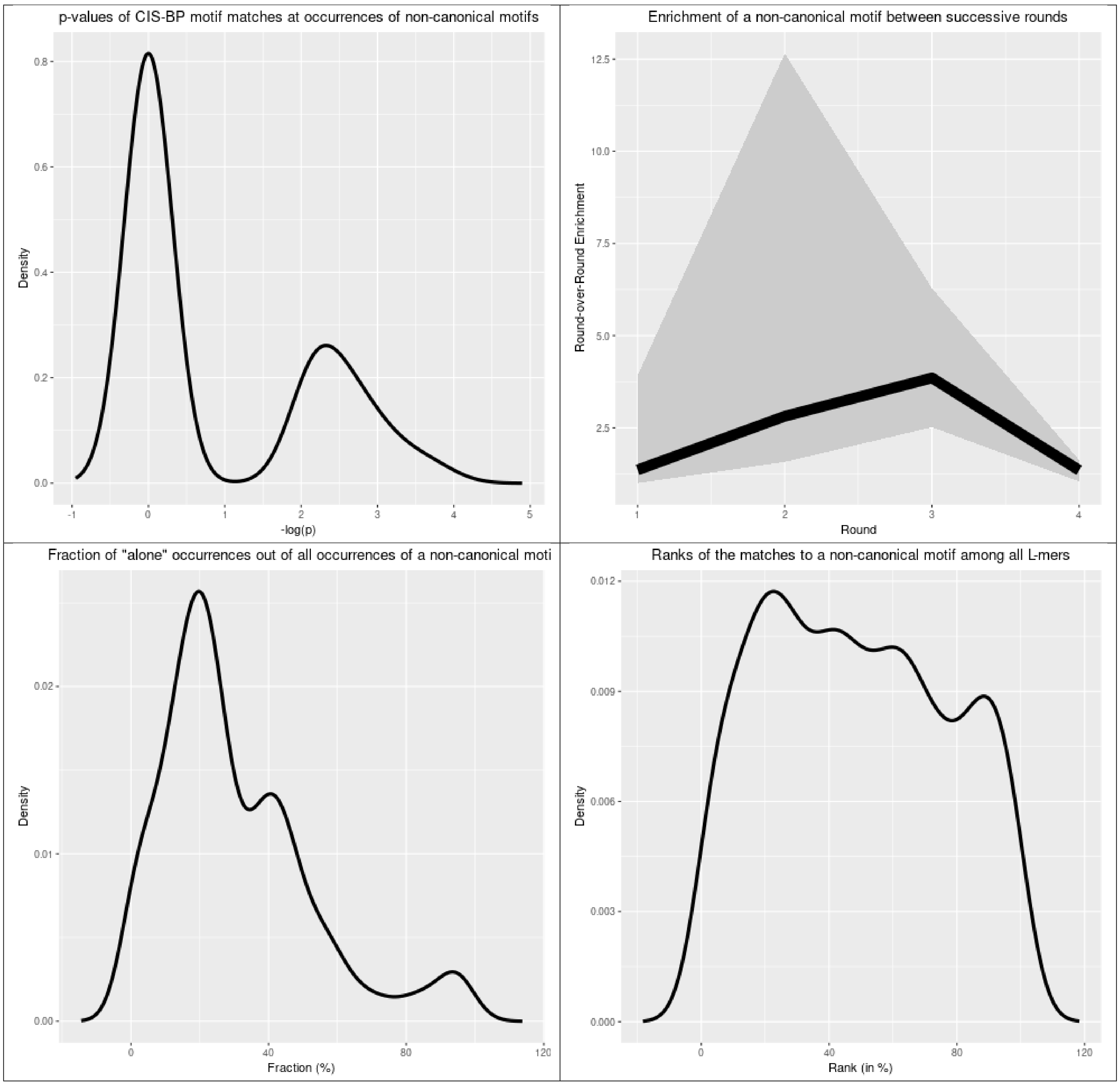

CIS-BP	Canonical	Non-canonical	Yin et al.
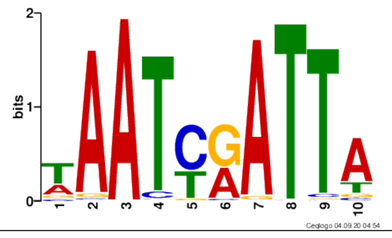	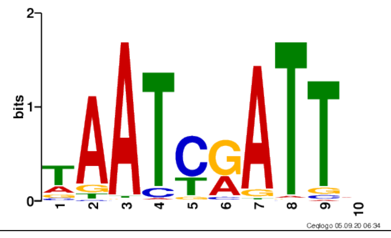	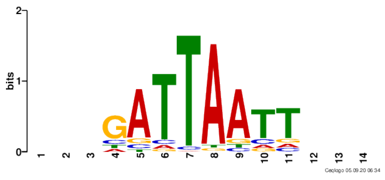	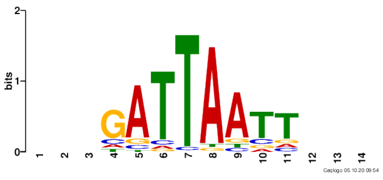
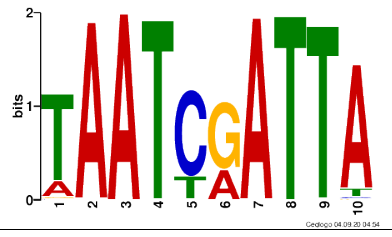	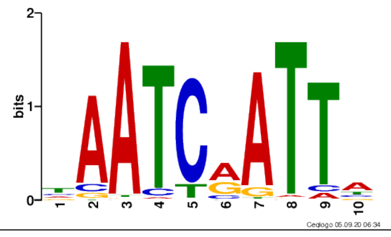		
	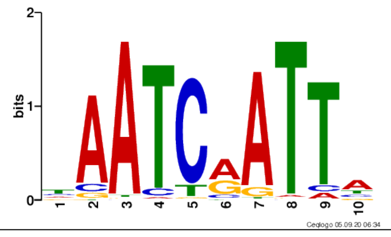		

Back to Table of Contents

17. PAX7, motif .1.1.2.1.3.1

Fractions of oligos explained (independently of CIS-BP motifs): 11.6935%
Distance: 0.108331941691
p-value: 0.000122

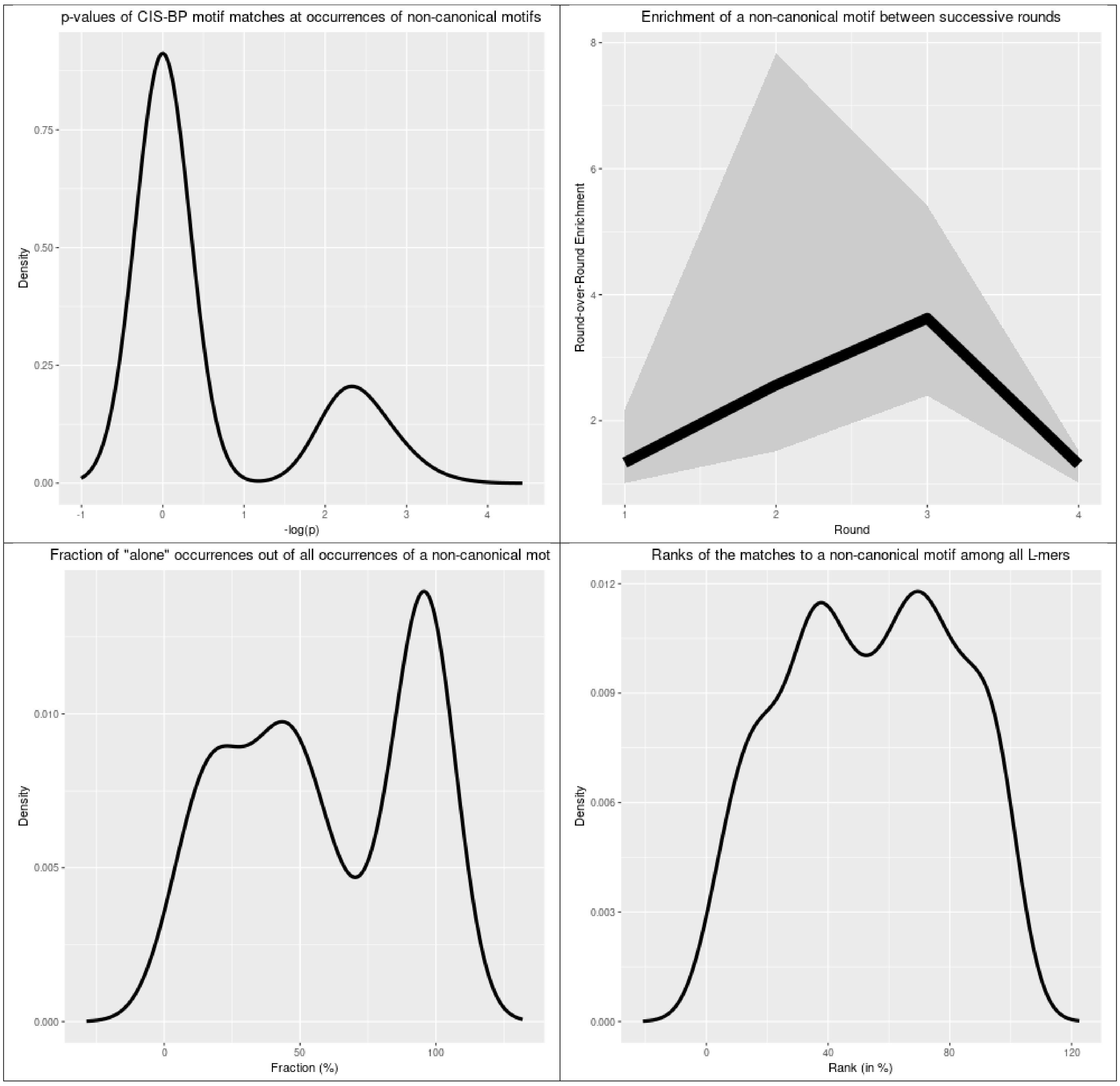

CIS-BP	Canonical	Non-canonical	Yin et al.
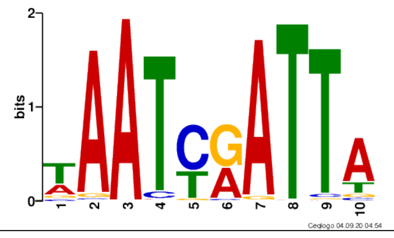	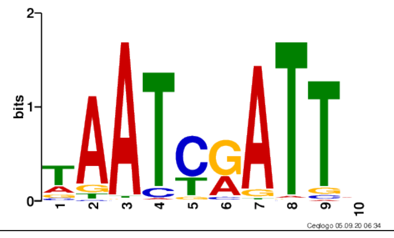	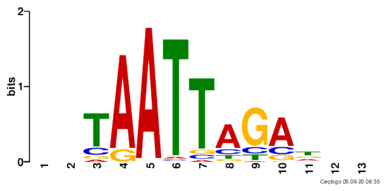	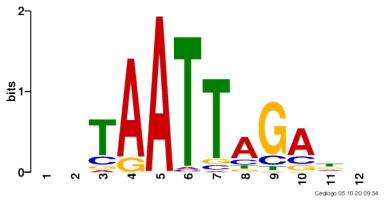
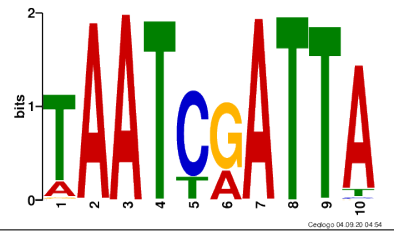	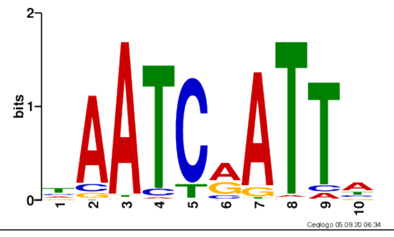		
	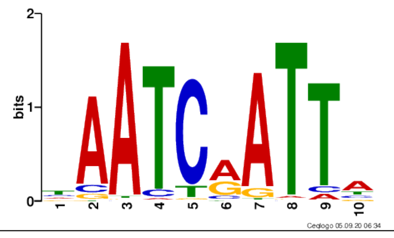		

Back to Table of Contents

18. ONECUT3, motif .1.1.2.1.3.1

Fractions of oligos explained (independently of CIS-BP motifs): 6.2826%
Distance: 0.221784327111
p-value: 0.000234

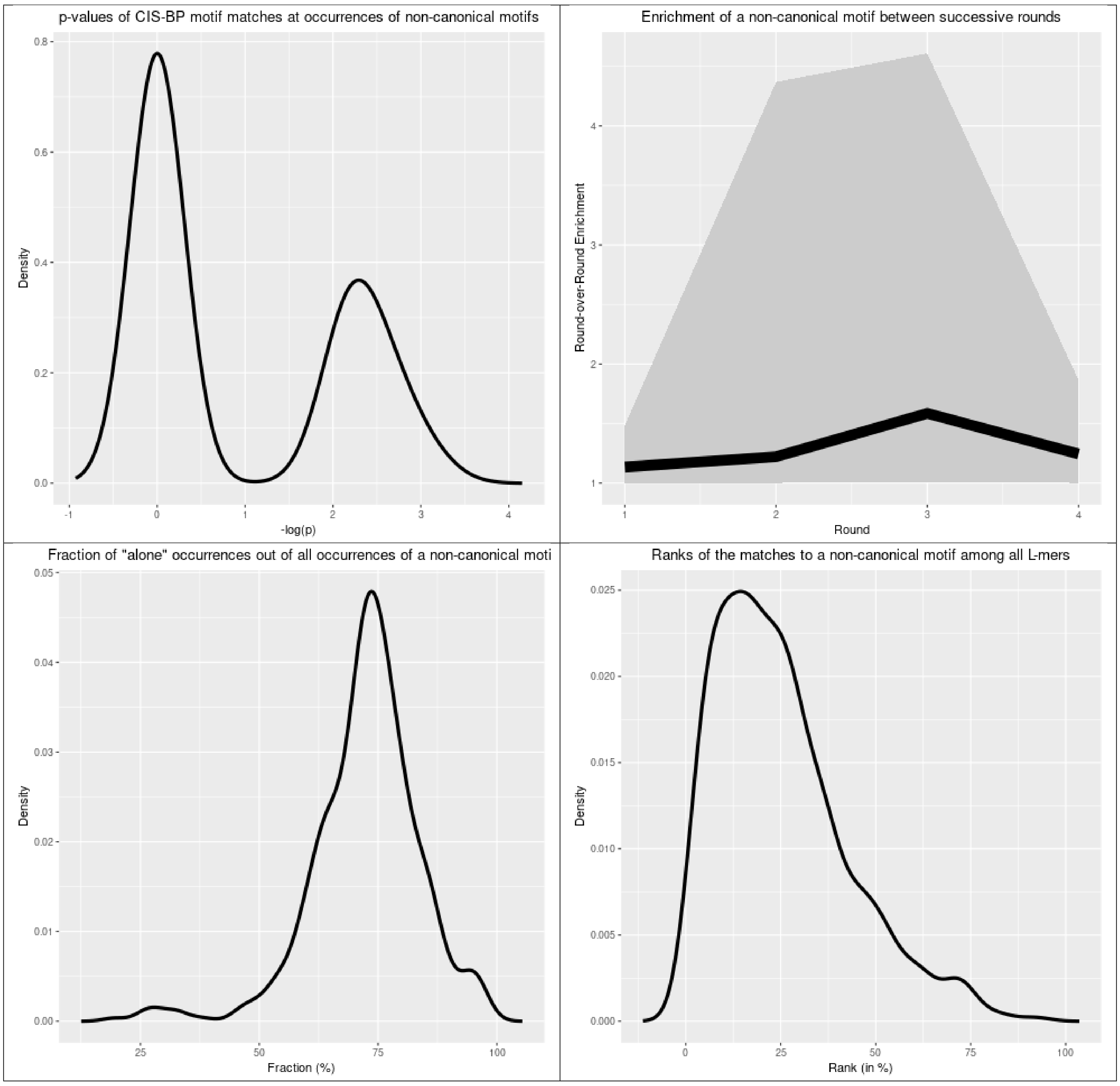

CIS-BP	Canonical	Non-canonical	Yin et al.
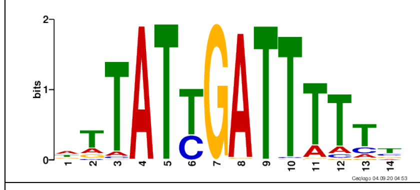	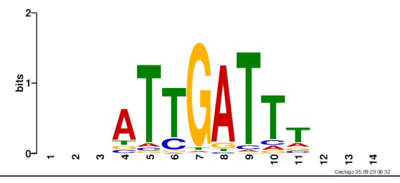	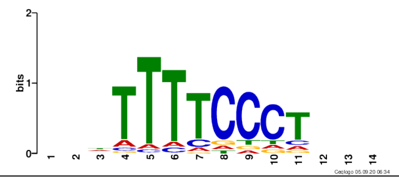	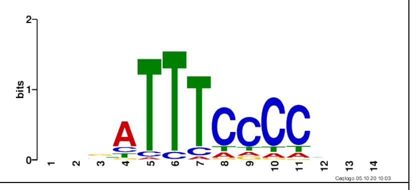
	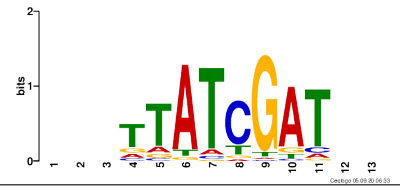		
	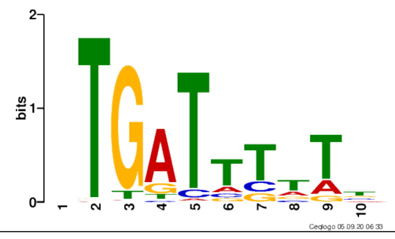		
	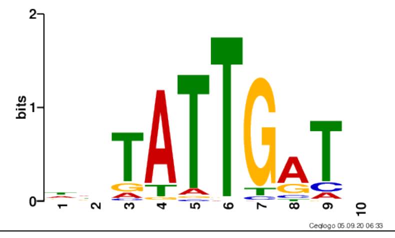		
	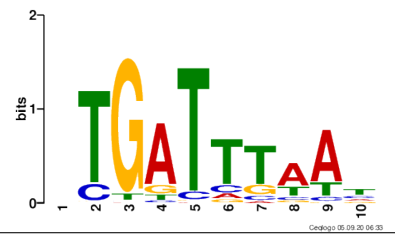		
	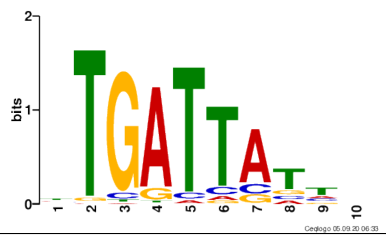		

Back to Table of Contents

19. ZNF784, MOTIF .1.1

Fractions of oligos explained (independently of CIS-BP motifs): 9.0462%
Distance: 0.283264601621
p-value: 0.006244

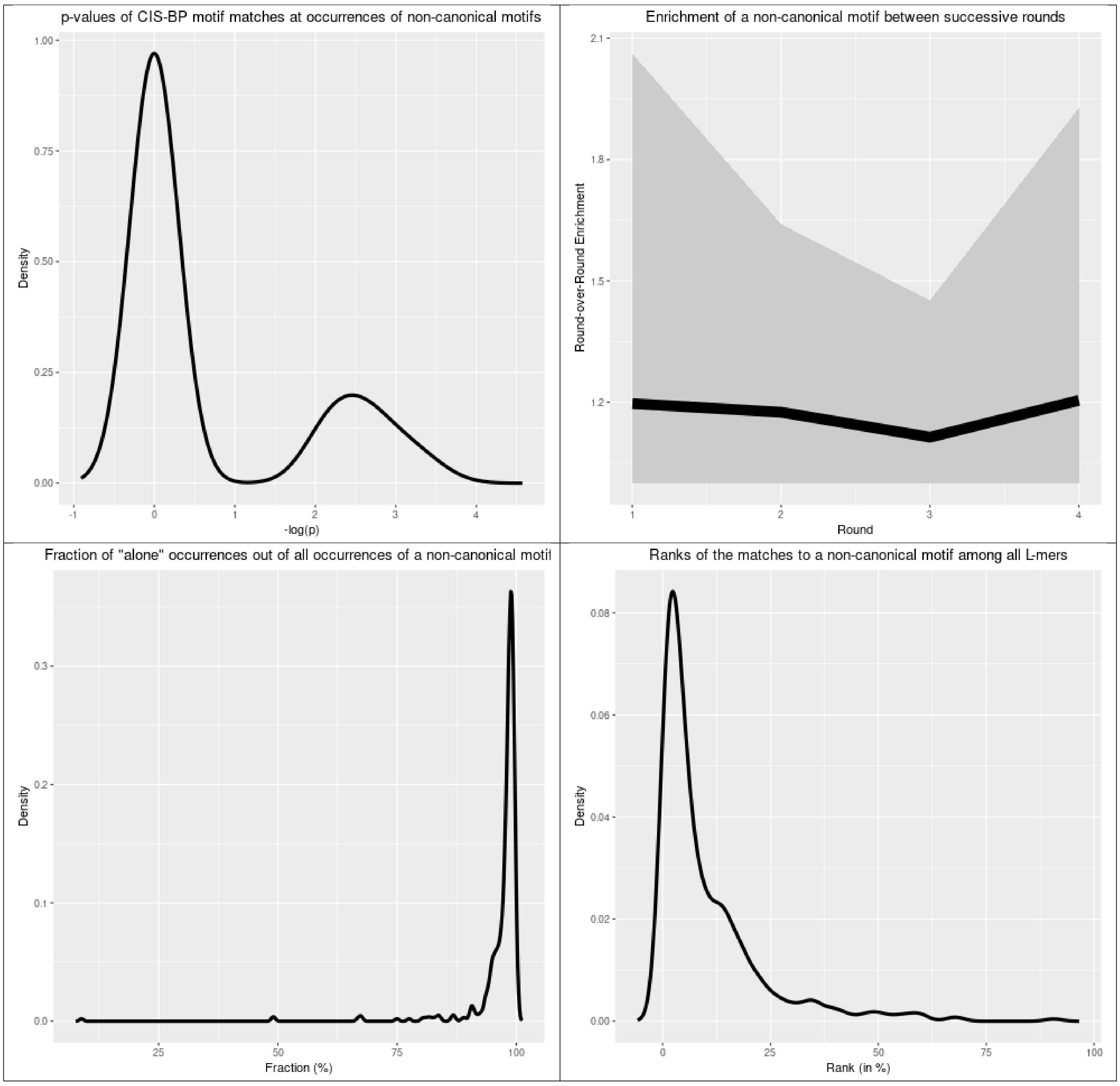

CIS-BP	Canonical	Non-canonical	Yin et al.
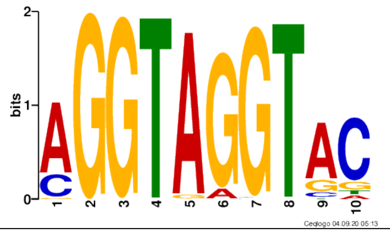	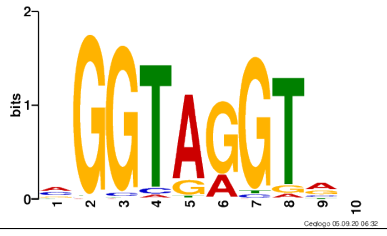	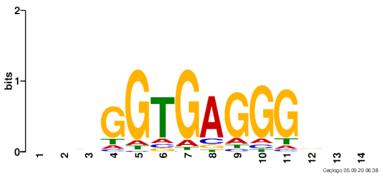	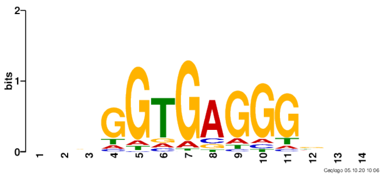
	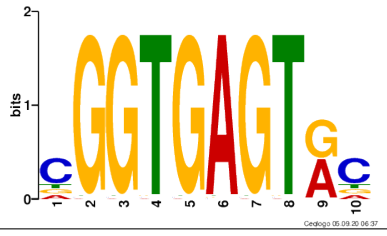		
	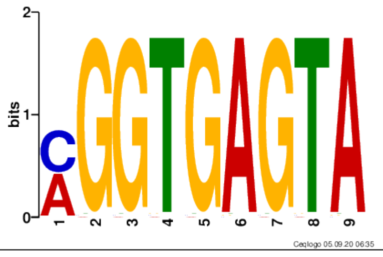		

Back to Table of Contents

20. ZBTB49, MOTIF .1.1

Fractions of oligos explained (independently of CIS-BP motifs): 6.1436%
Distance: 0.276949607225
p-value: 0.000001

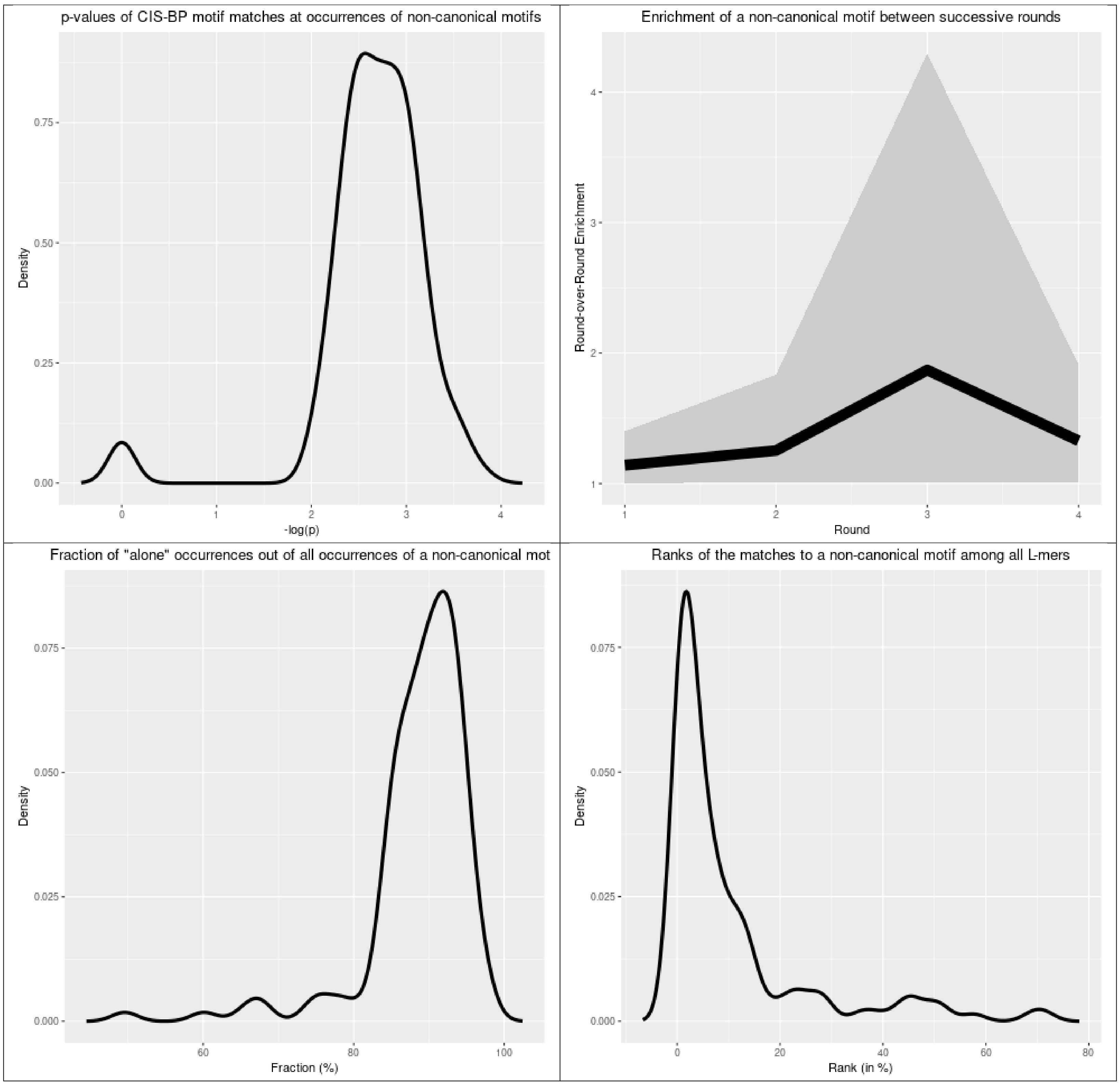

CIS-BP	Canonical	Non-canonical	Yin et al.
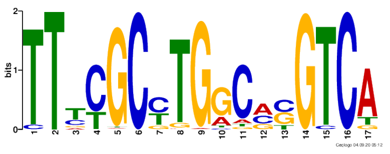	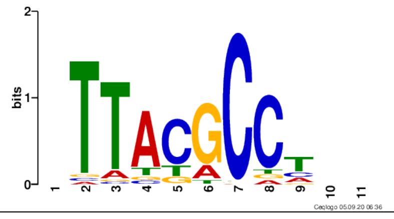	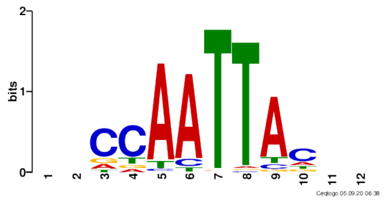	NA
	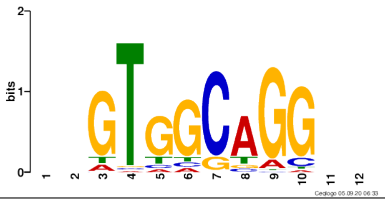		
	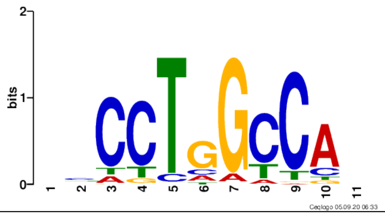		
	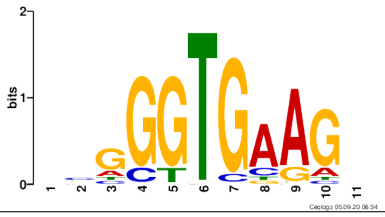		
	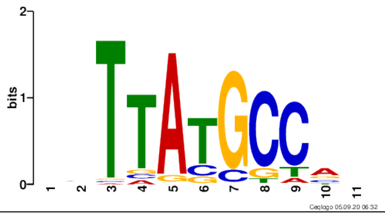		
	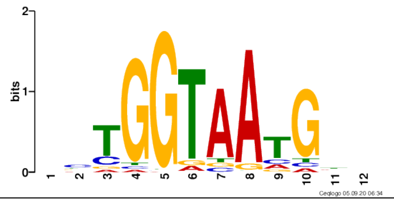		

Back to Table of Contents

21. PDX1, motif .1.1

Fractions of oligos explained (independently of CIS-BP motifs): 5.1974%
Distance: 0.265071694848
p-value: 0.000000

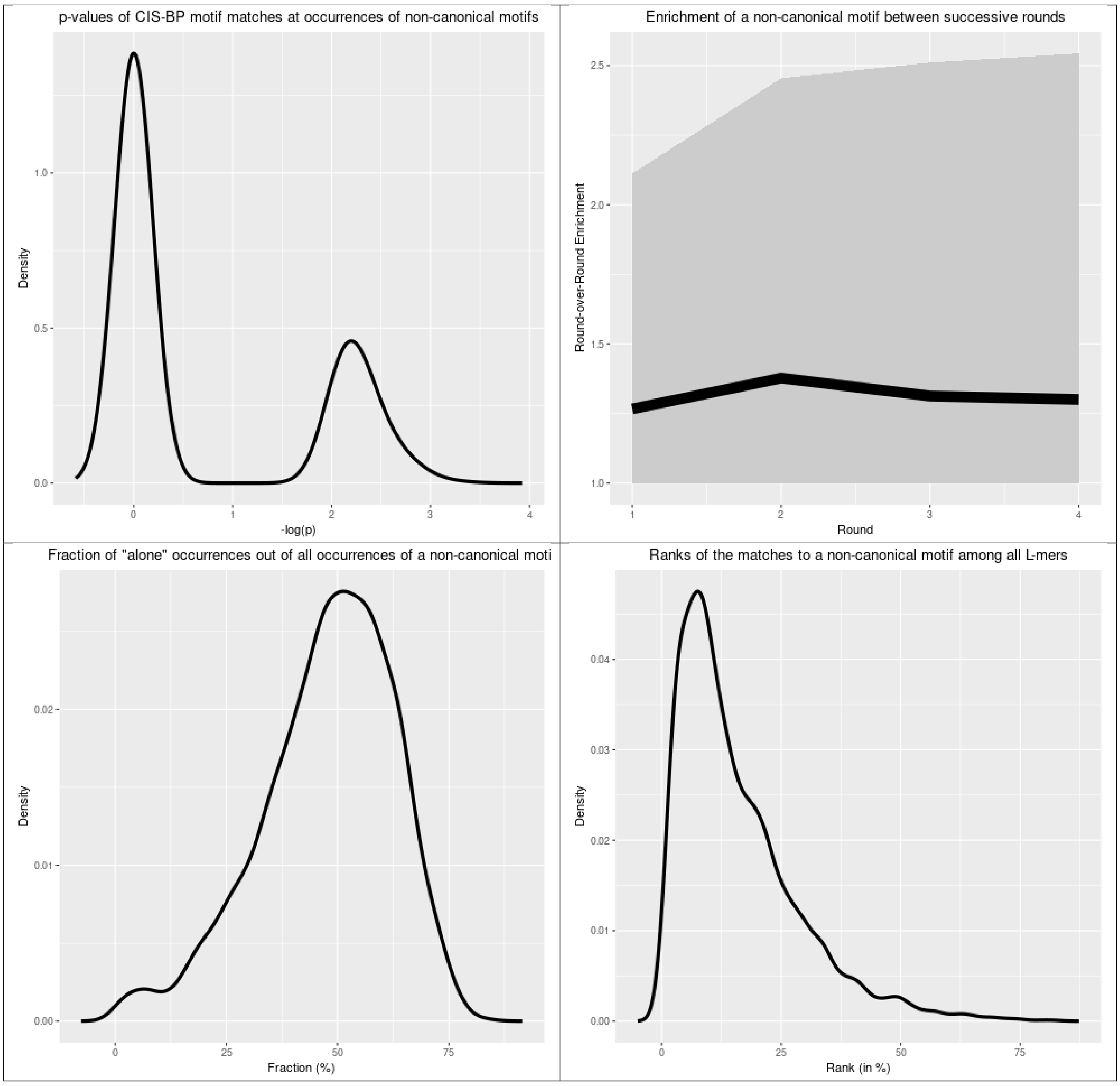

CIS-BP	Canonical	Non-canonical	Yin et al.
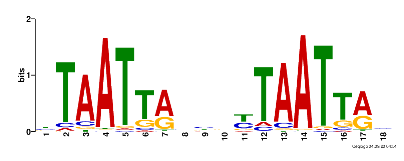	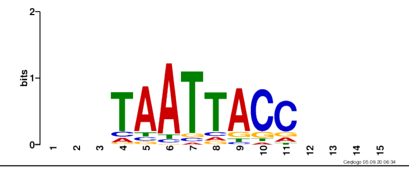	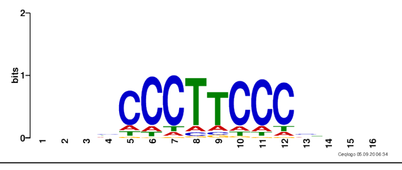	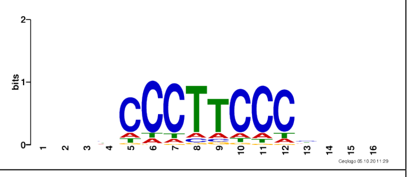
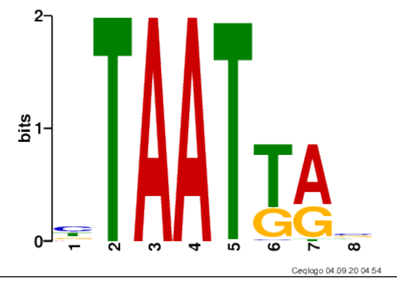	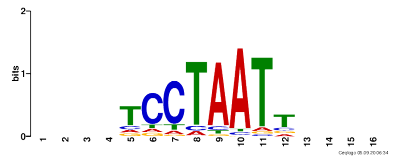		
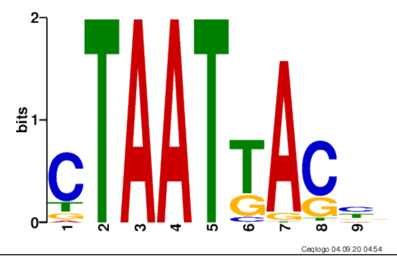	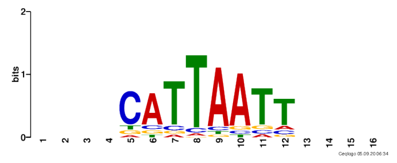		
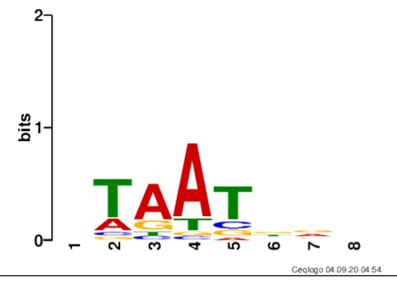	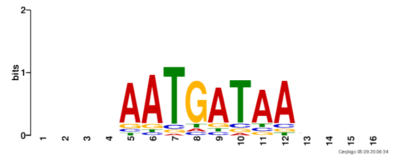		
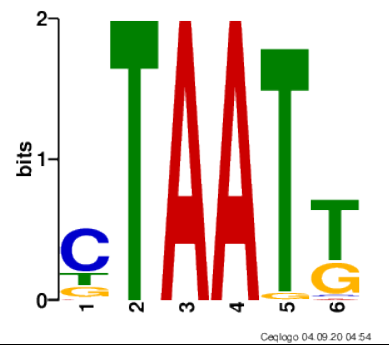	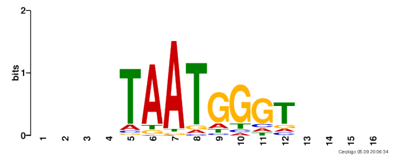		

Back to Table of Contents

22. CEBPG, motif .1.1.2.1.3.1

Fractions of oligos explained (independently of CIS-BP motifs): 5.1124%
Distance: 0.248622930015
p-value: 0.000002

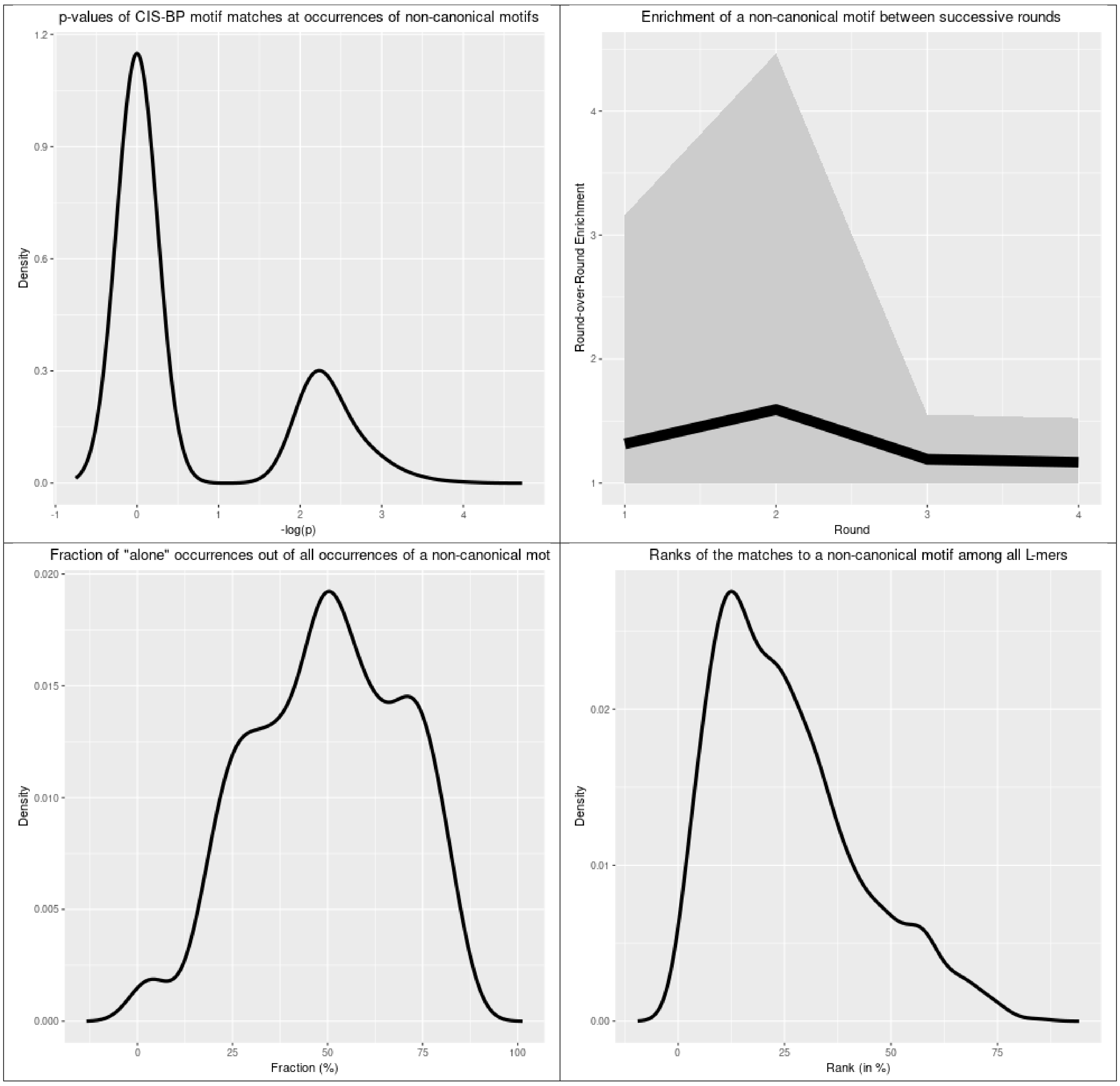

CIS-BP	Canonical	Non-canonical	Yin et al.
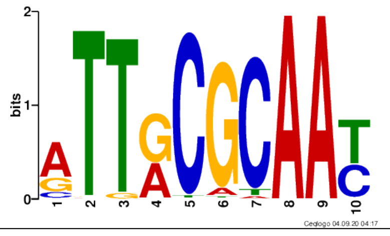	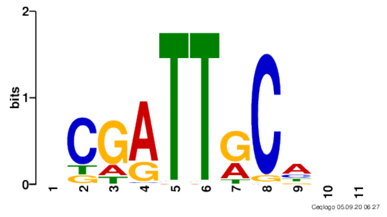	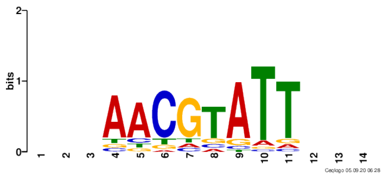	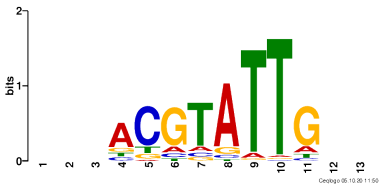
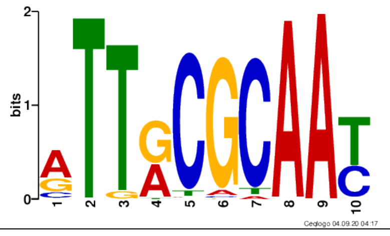	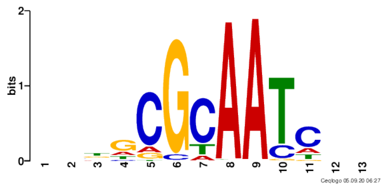		
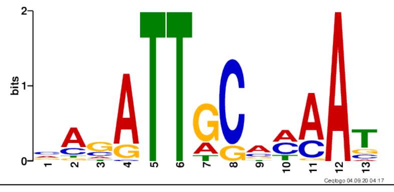	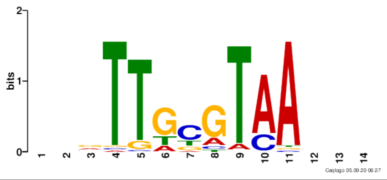		
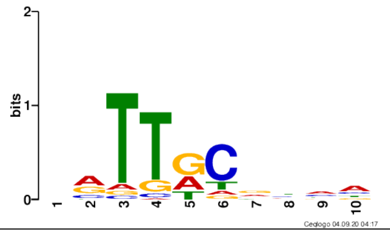	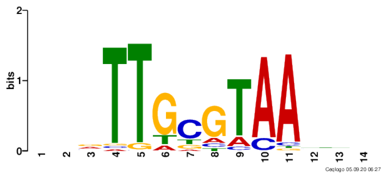		
	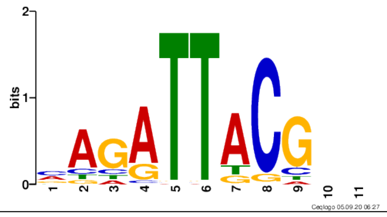		
	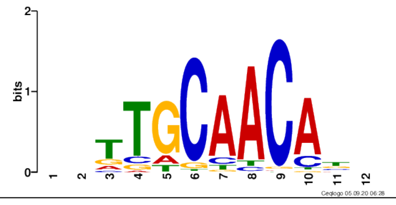		
	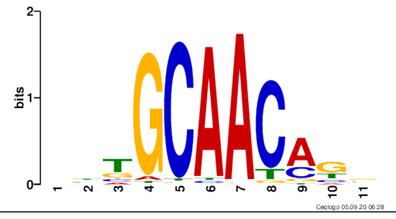		

Back to Table of Contents

23. POU2F3, MOTIF .1.1

Fractions of oligos explained (independently of CIS-BP motifs): 9.7421%
Distance: 0.209658489204
p-value: 0.000250

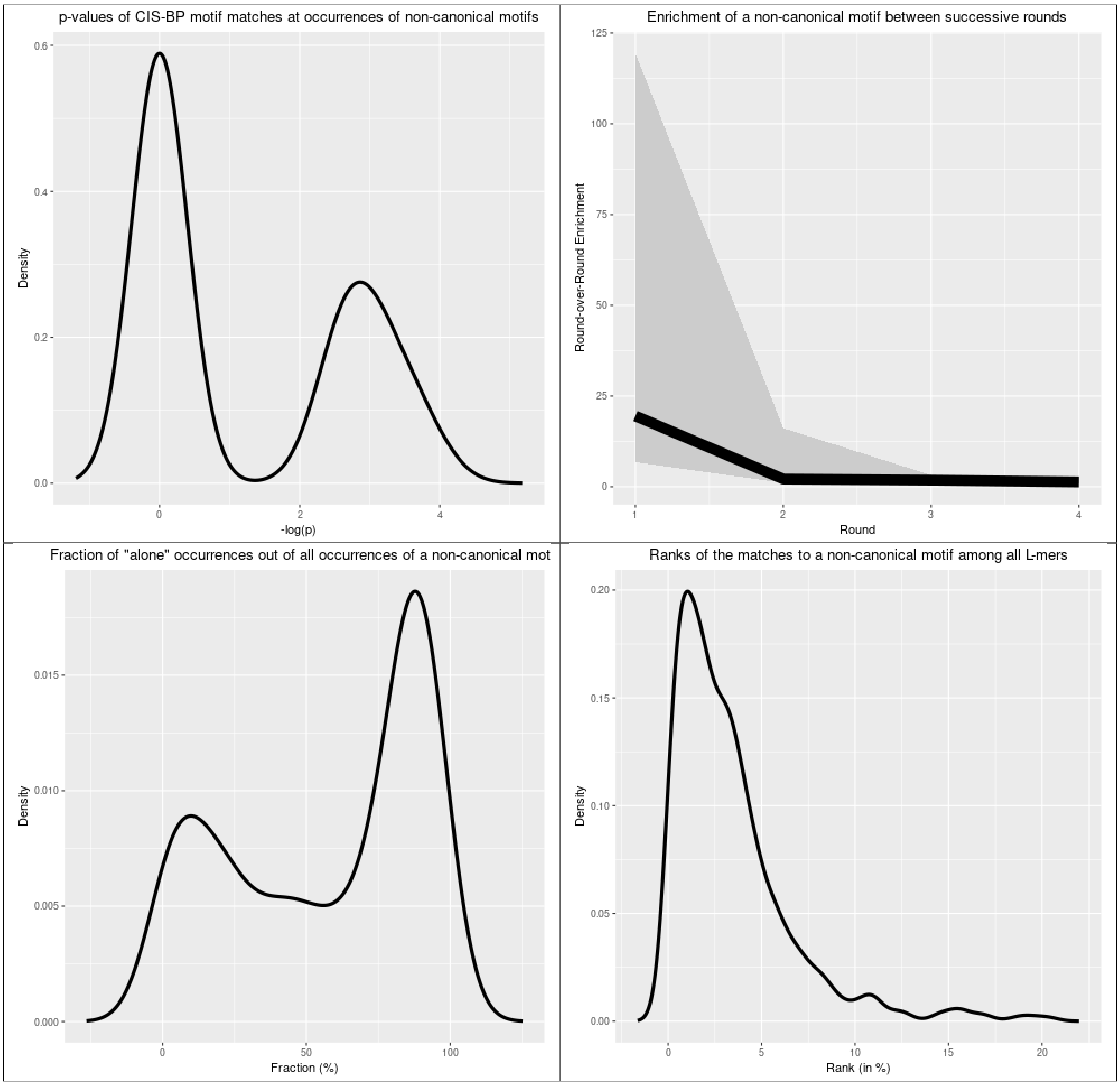

CIS-BP	Canonical	Non-canonical	Yin et al.
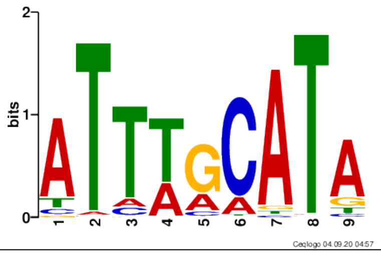	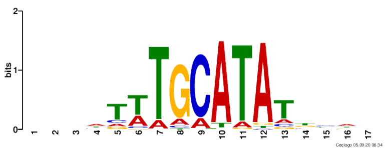	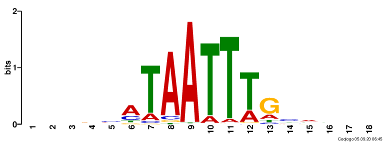	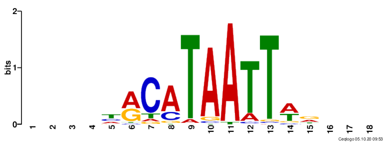
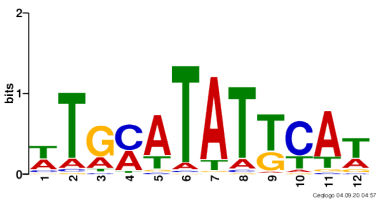	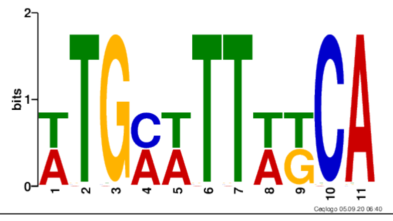		
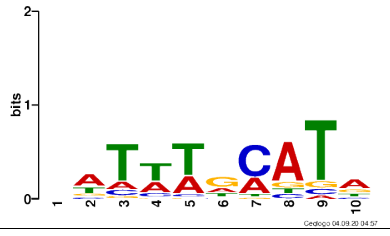	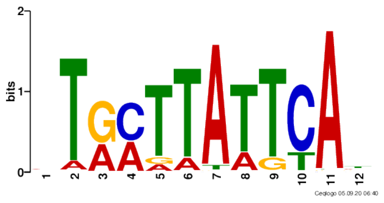		
	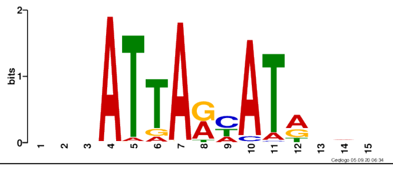		
	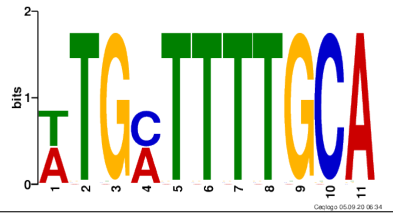		
	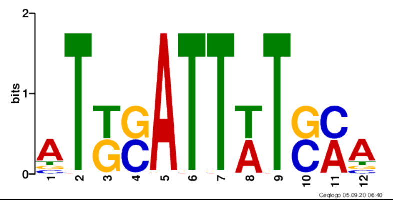		

Back to Table of Contents

24. NKX3-1, motif .1.1.2.1

Fractions of oligos explained (independently of CIS-BP motifs): 6.0151%
Distance: 0.201012010957
p-value: 0.000972

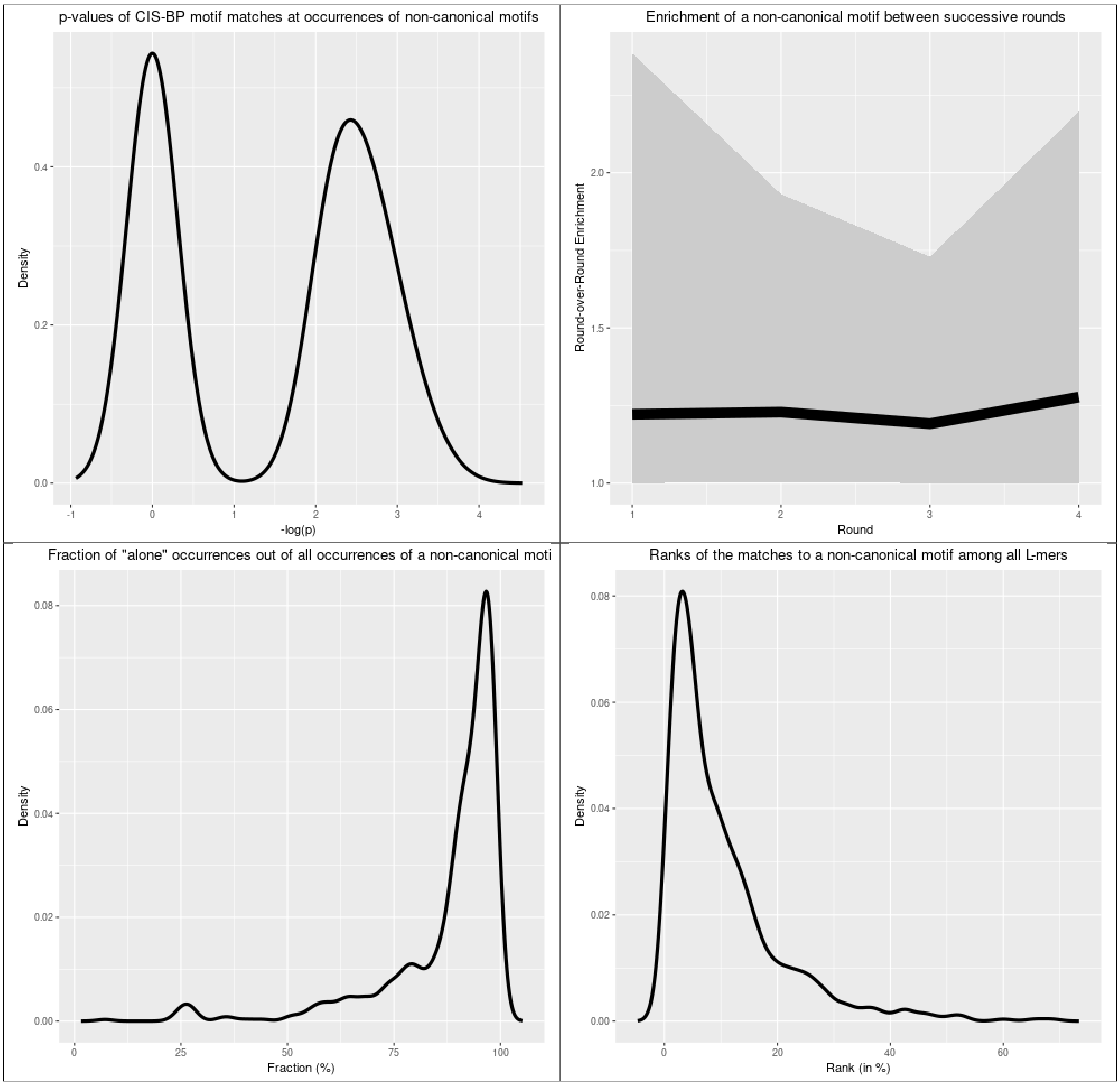

CIS-BP	Canonical	Non-canonical	Yin et al.
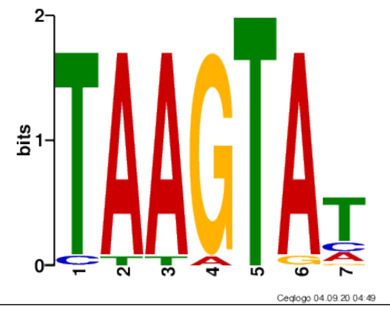	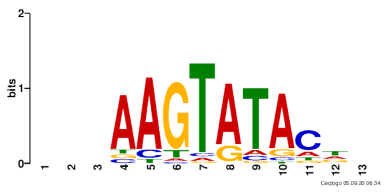	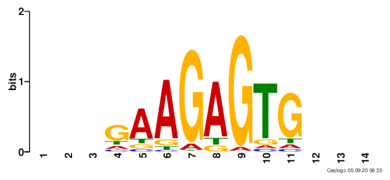	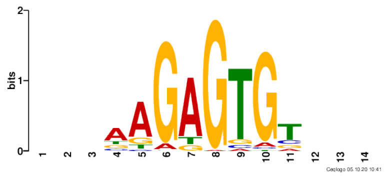
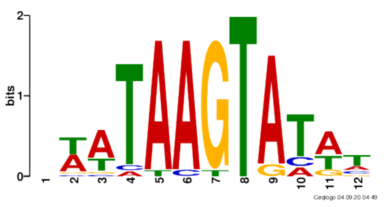	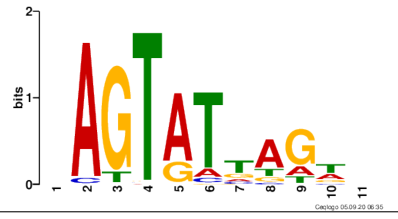		
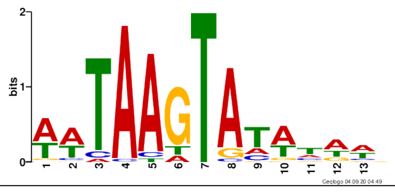	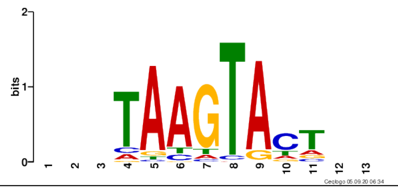		
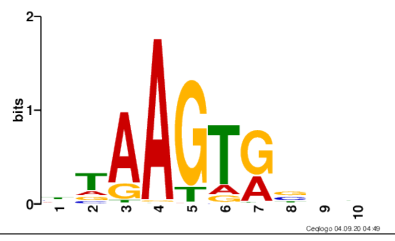	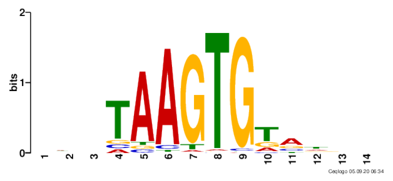		
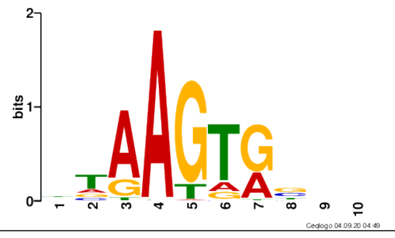	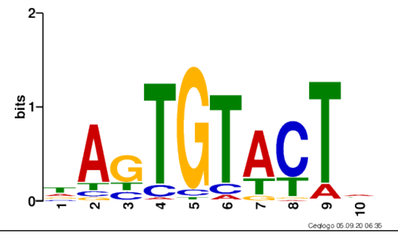		

Back to Table of Contents

25. HOXC10, MOTIF .1.1

Fractions of oligos explained (independently of CIS-BP motifs): 8.7191%
Distance: 0.116457070286
p-value: 0.007445

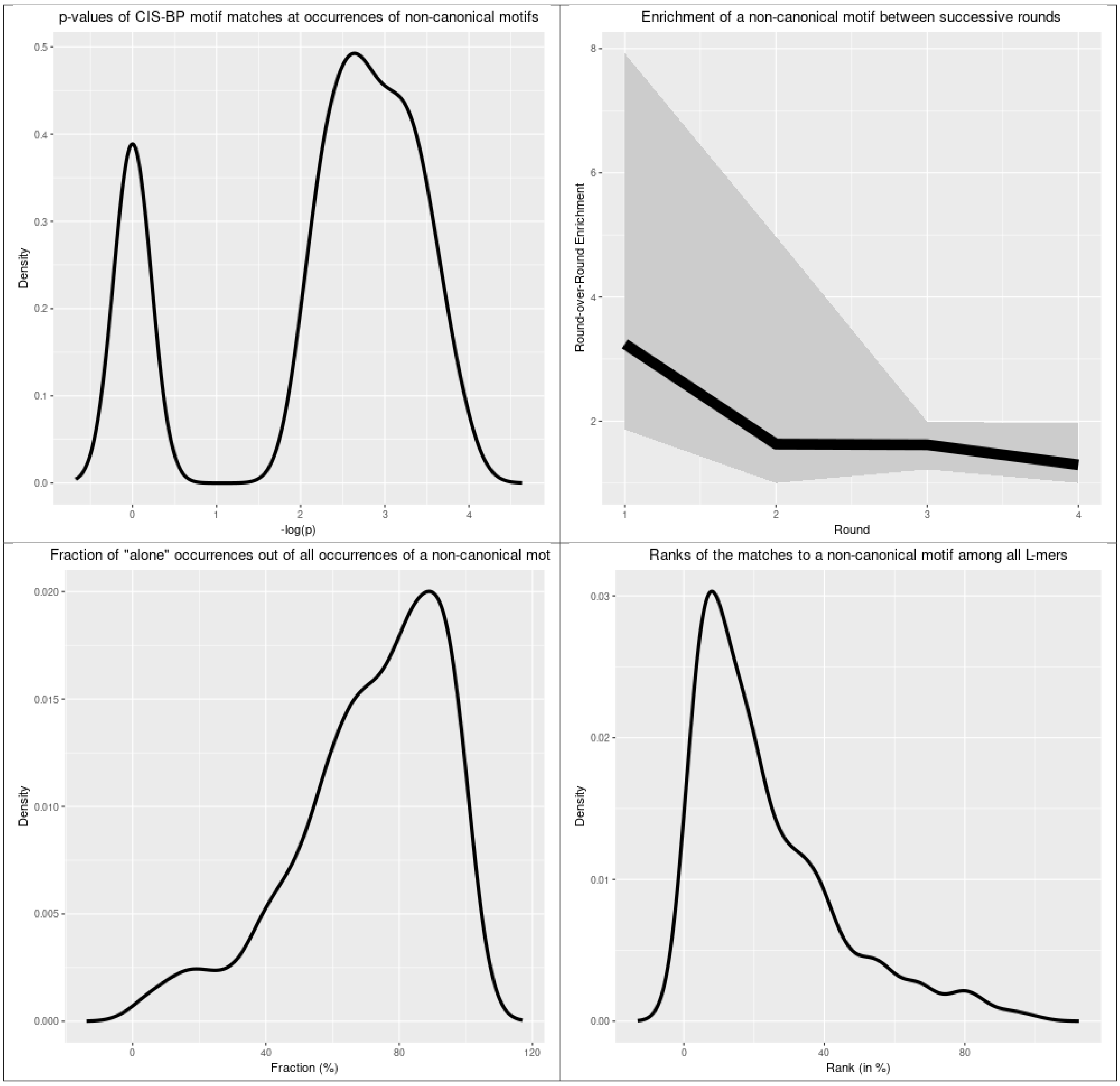

CIS-BP	Canonical	Non-canonical	Yin et al.
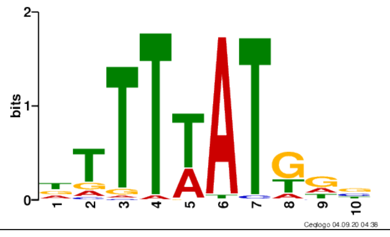	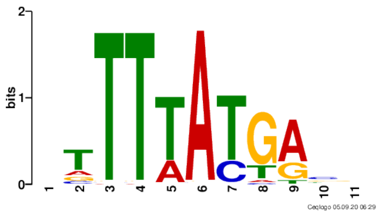	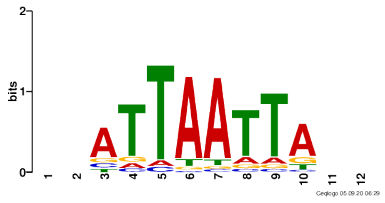	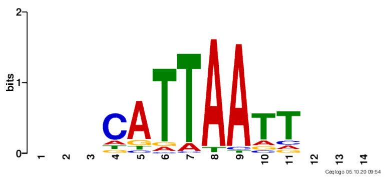
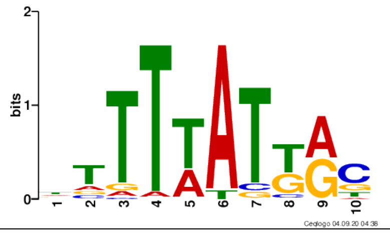	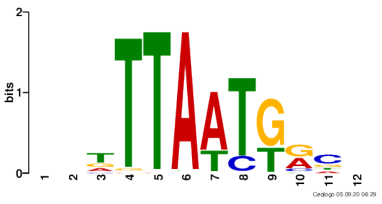		
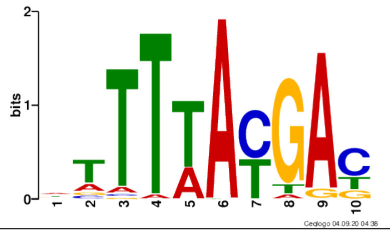			
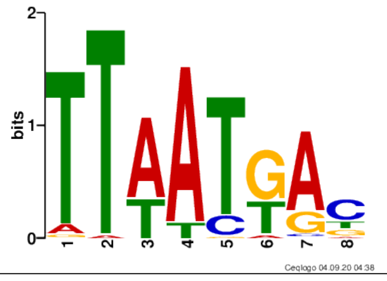			
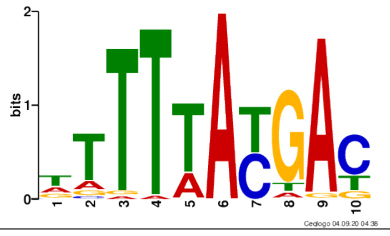			
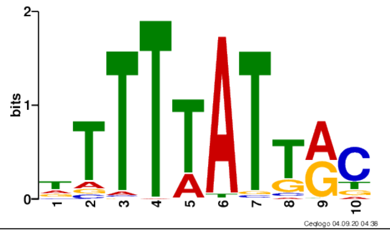			

Back to Table of Contents

26. CEBPG, motif .1.2.2.1

Fractions of oligos explained (independently of CIS-BP motifs): 5.4183%
Distance: 0.17219311329
p-value: 0.010332

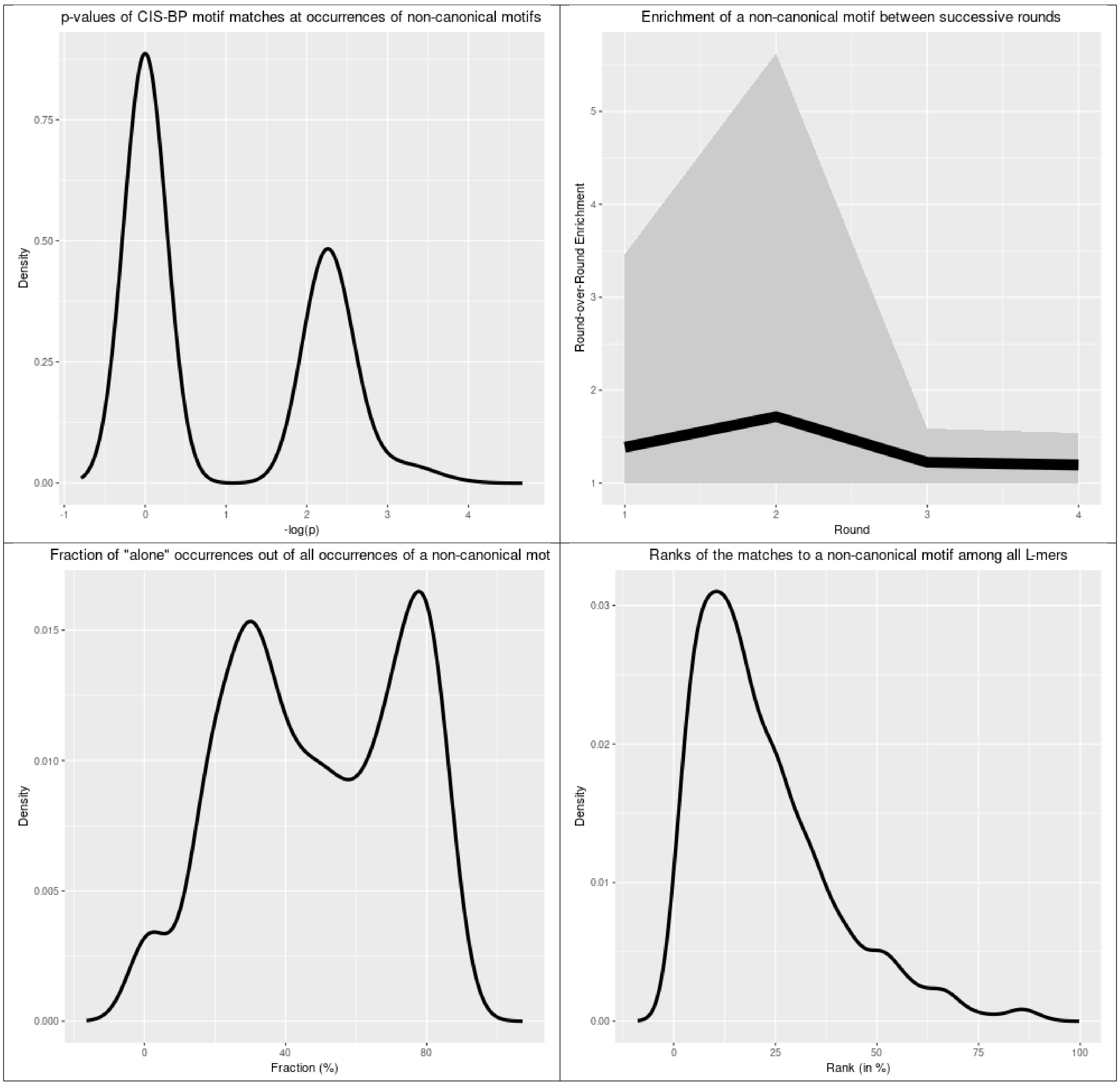

CIS-BP	Canonical	Non-canonical	Yin et al.
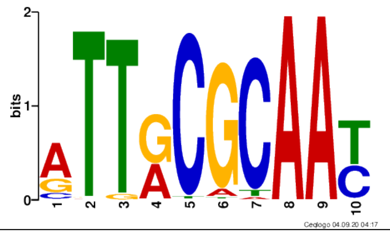	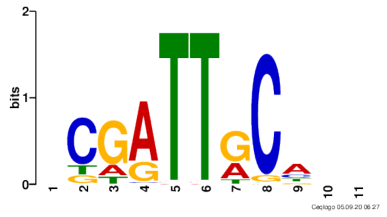	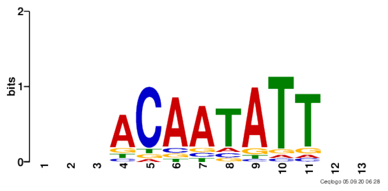	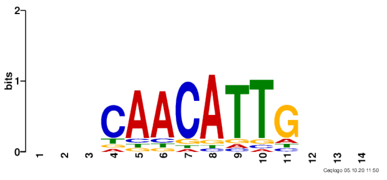
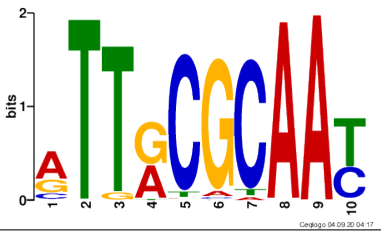	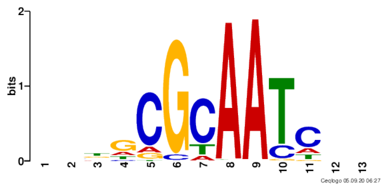		
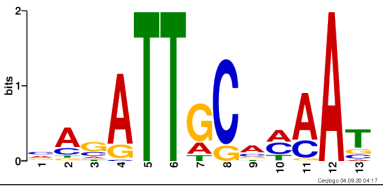	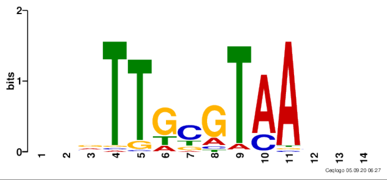		
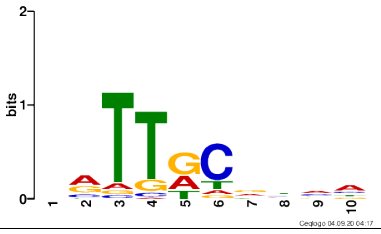	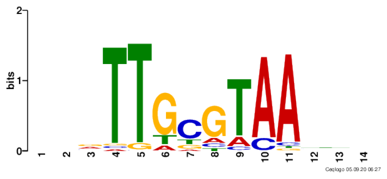		
	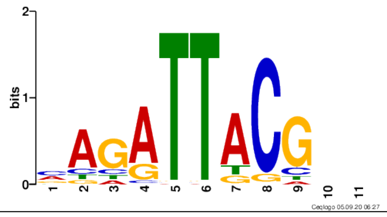		
	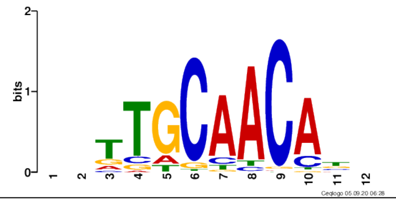		
	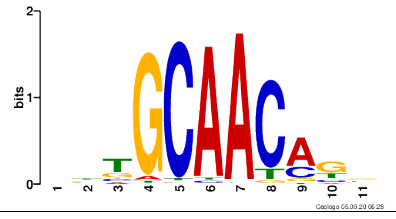		

Back to Table of Contents

27. NKX3-1, motif .1.1.2.1.3.1

Fractions of oligos explained (independently of CIS-BP motifs): 7.0100%
Distance: 0.195402070963
p-value: 0.001710

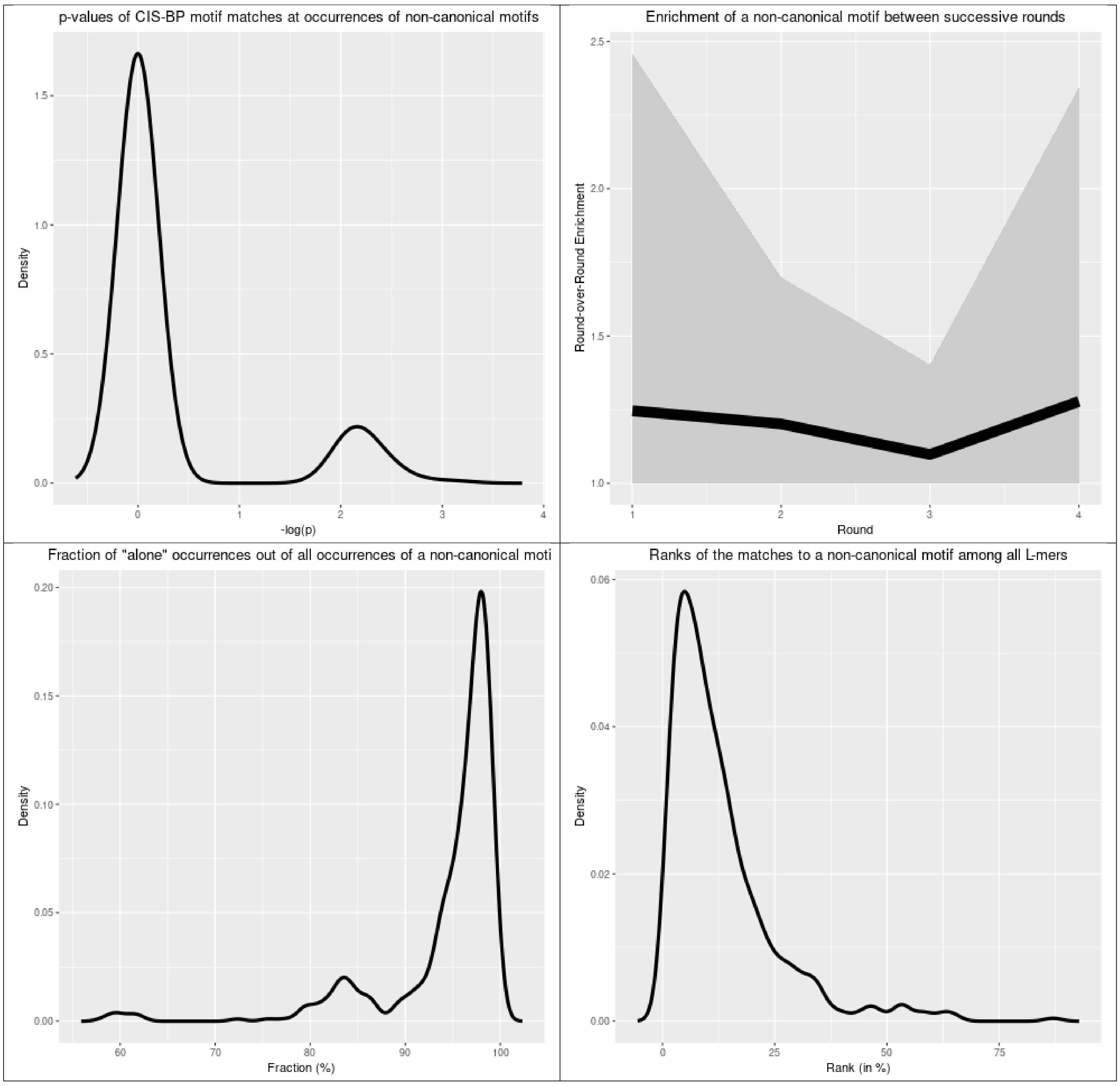

CIS-BP	Canonical	Non-canonical	Yin et al.
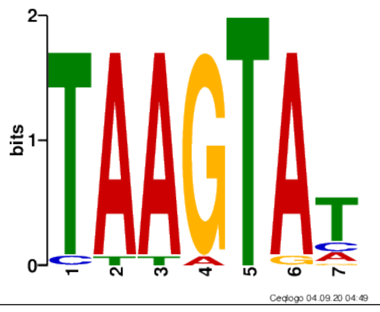	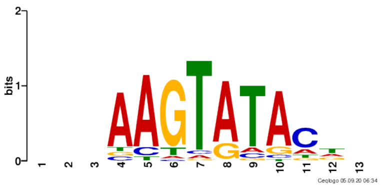	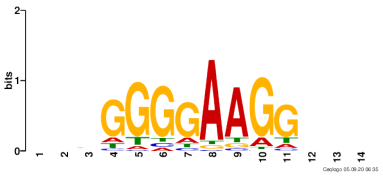	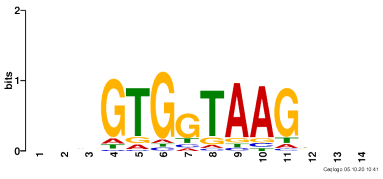
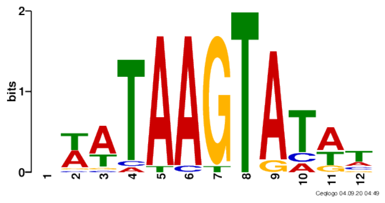	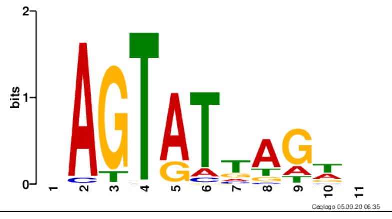		
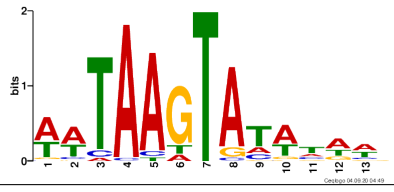	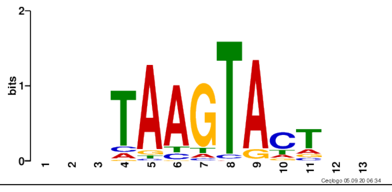		
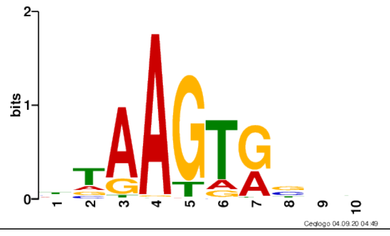	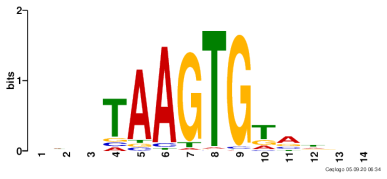		
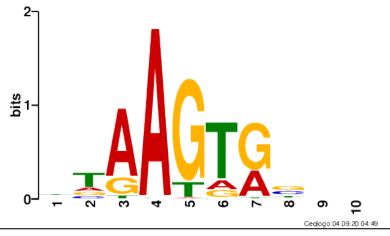	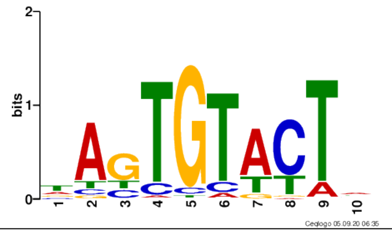		

Back to Table of Contents

28. NOTO, MOTIF .1.1.2.1

Fractions of oligos explained (independently of CIS-BP motifs): 14.1016%
Distance: 0.284022915358
p-value: 0.000000

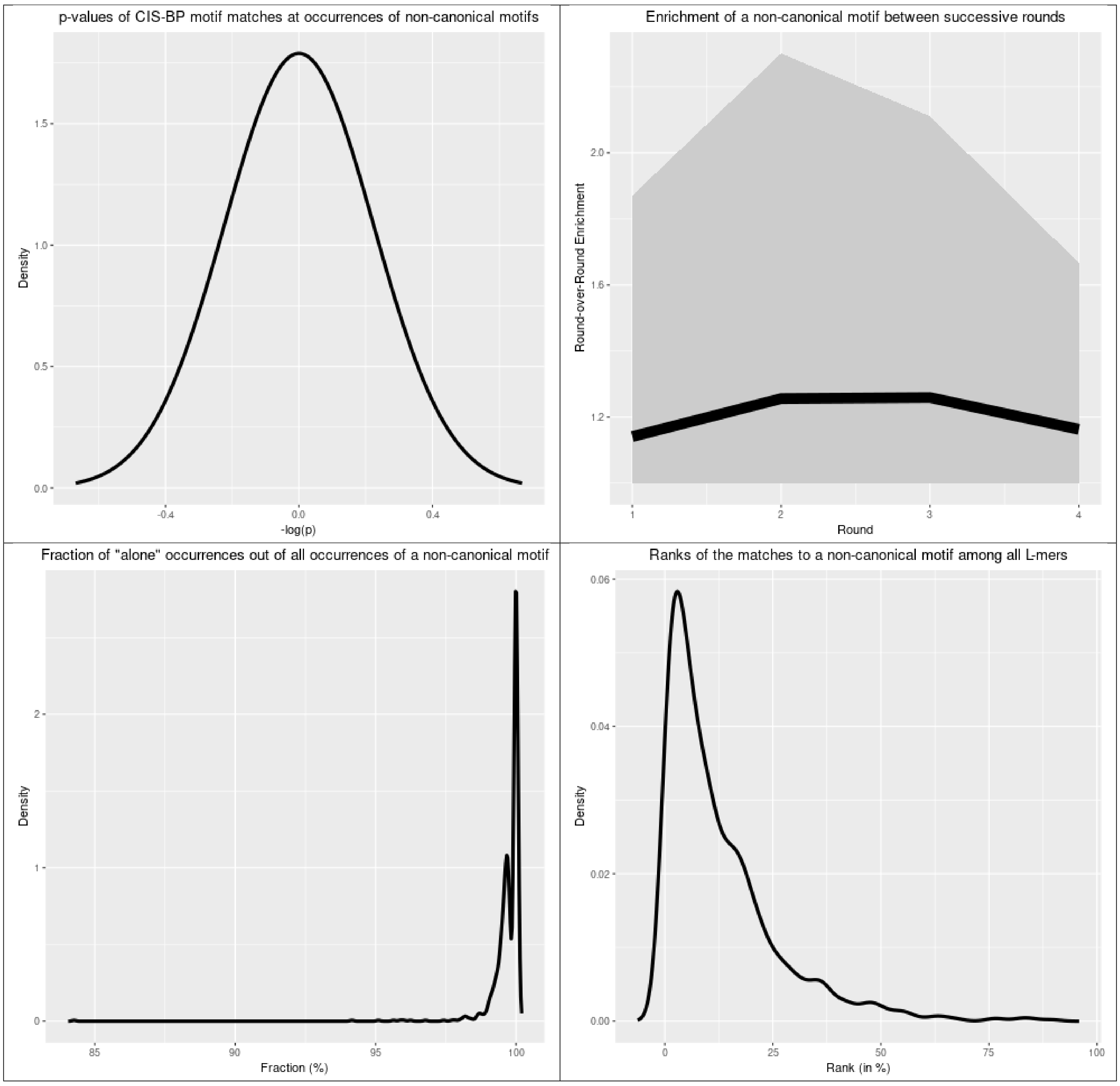

CIS-BP	Canonical	Non-canonical	Yin et al.
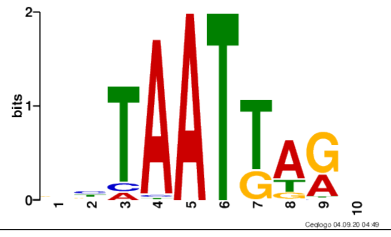	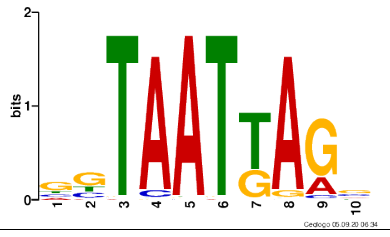	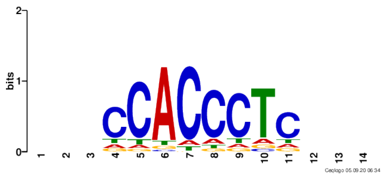	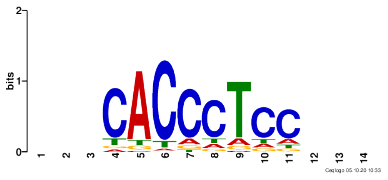
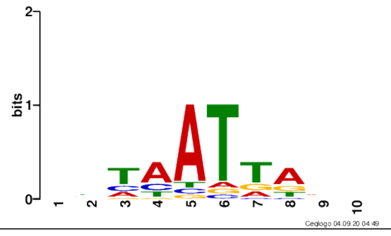			

Back to Table of Contents

29. NOTO, MOTIF .1.1.2.1.3.1

Fractions of oligos explained (independently of CIS-BP motifs): 12.0231%
Distance: 0.258050736819
p-value: 0.000000

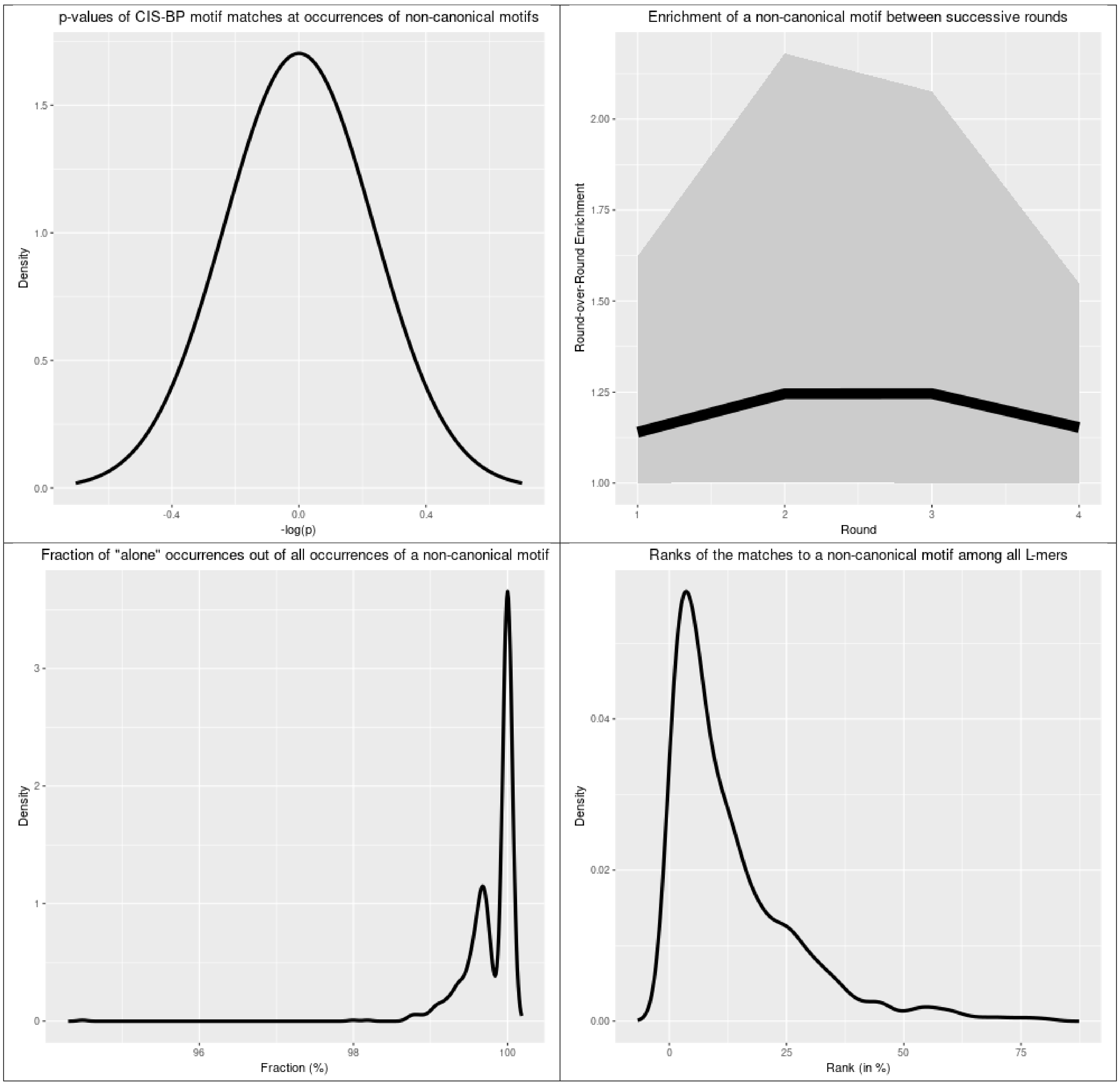

CIS-BP	Canonical	Non-canonical	Yin et al.
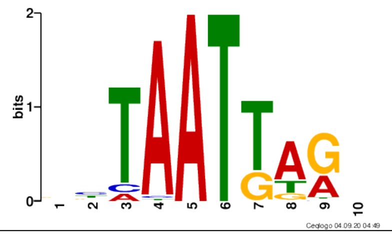	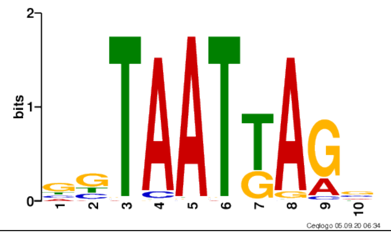	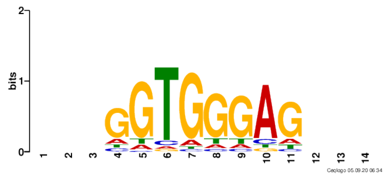	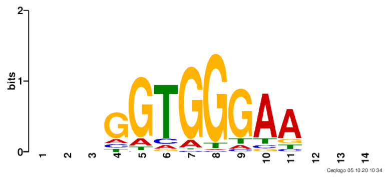
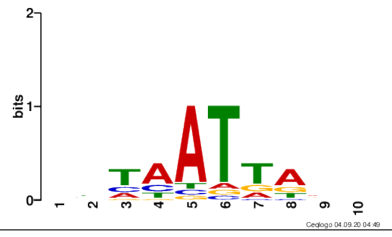			

Back to Table of Contents

30. ZNF740, motif .1.1

Fractions of oligos explained (independently of CIS-BP motifs): 6.2046%
Distance: 0.260564464446
p-value: 0.005672

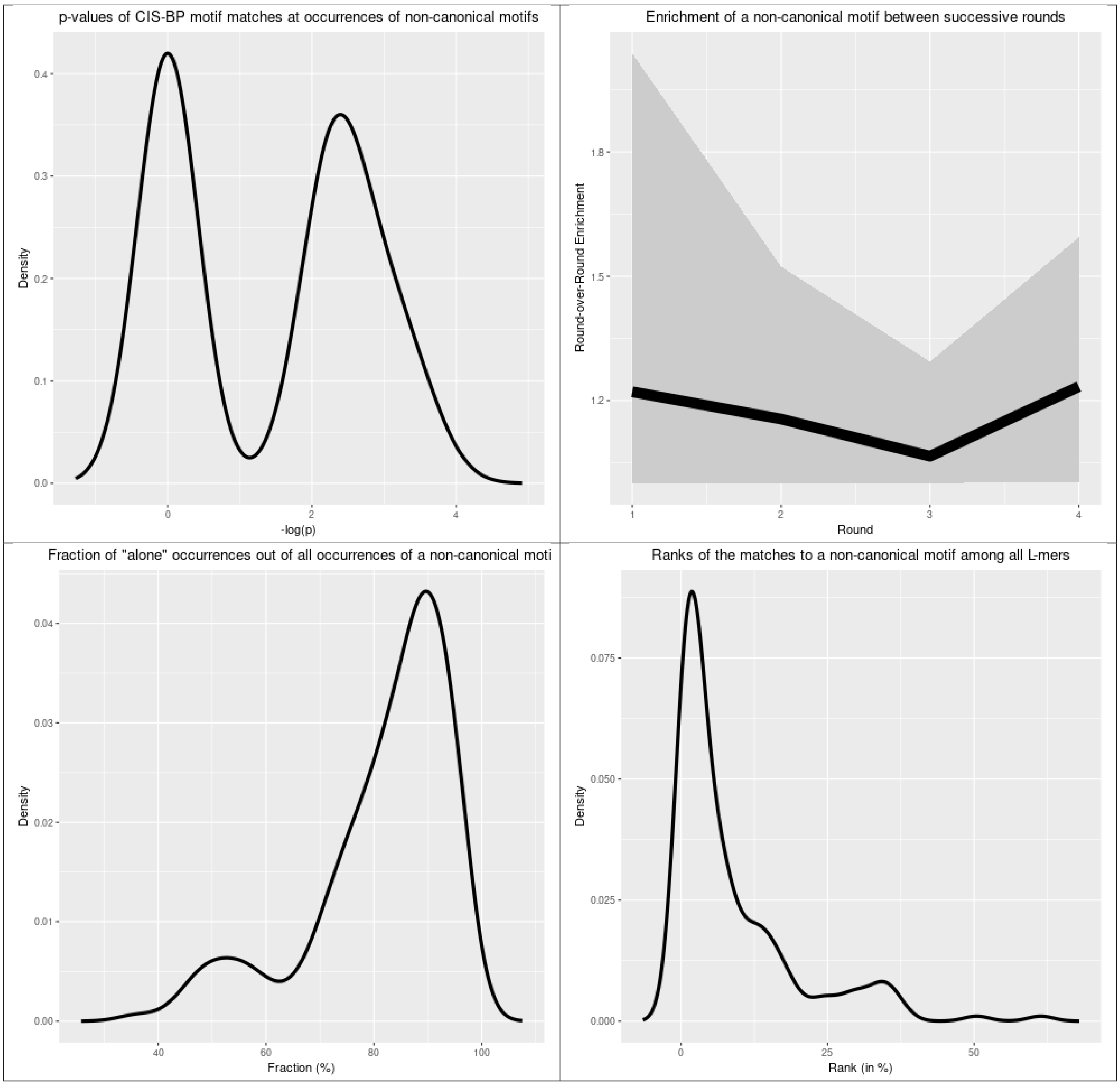

CIS-BP	Canonical	Non-canonical	Yin et al.
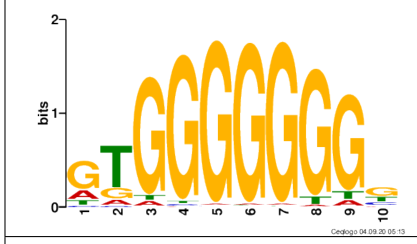	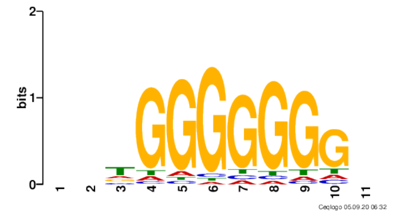	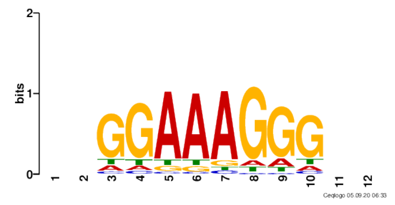	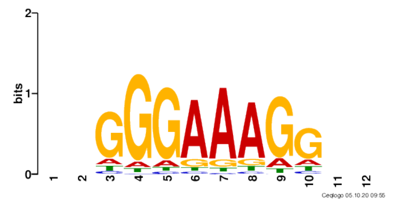
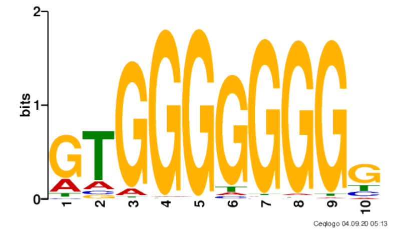	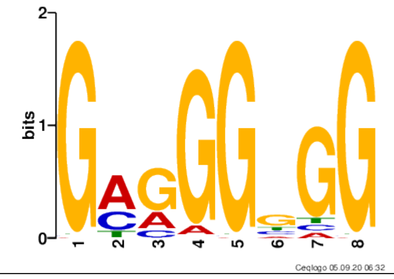		
	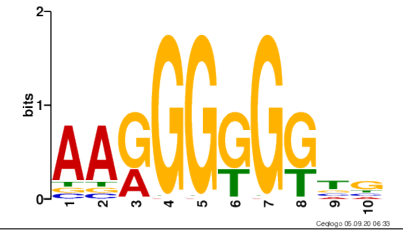		

Back to Table of Contents

31. FOXI1, motif .1.1.2.1.3.1.4.1

Fractions of oligos explained (independently of CIS-BP motifs): 6.1257%
Distance: 0.240646948646
p-value: 0.003439

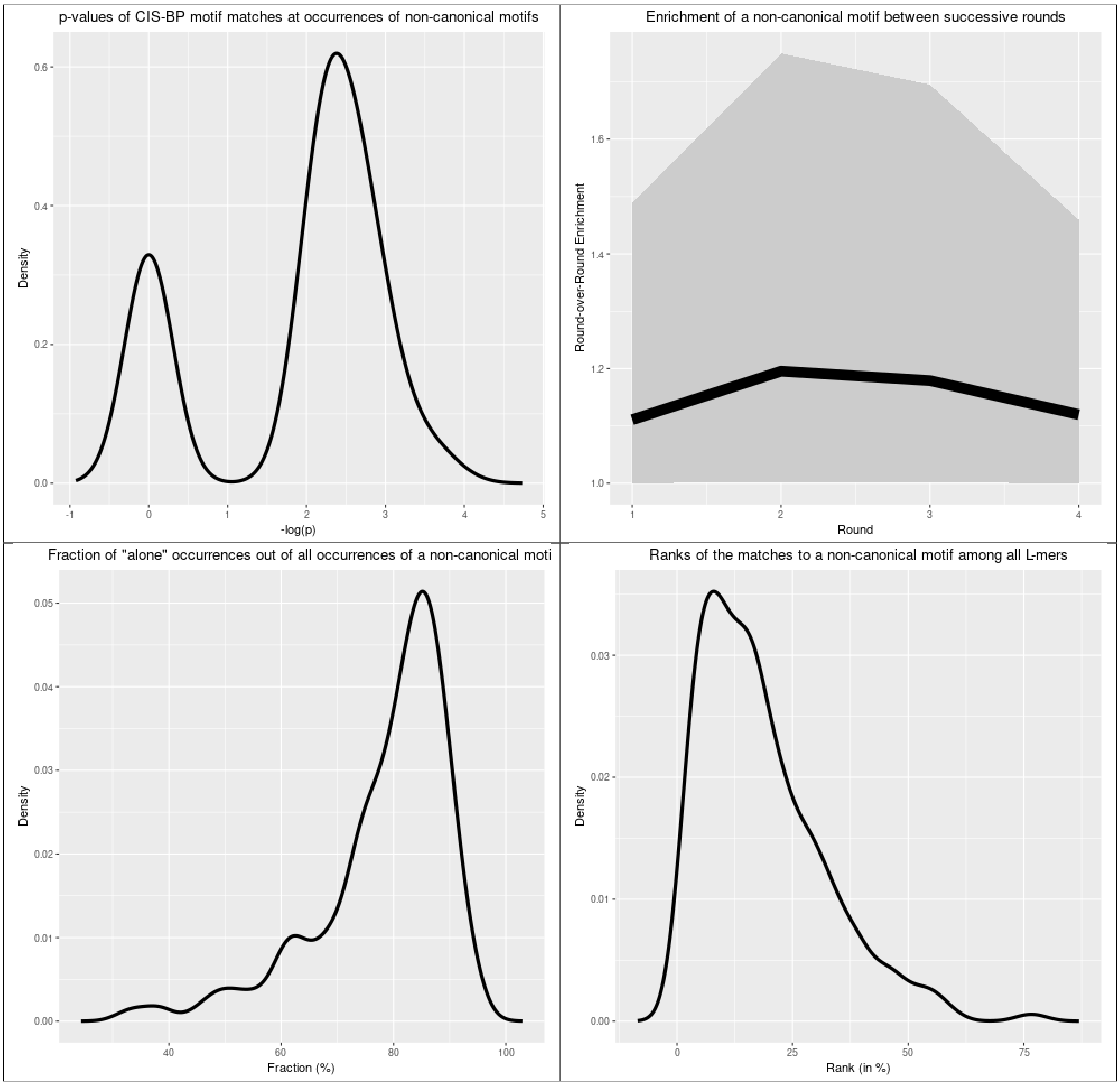

CIS-BP	Canonical	Non-canonical	Yin et al.
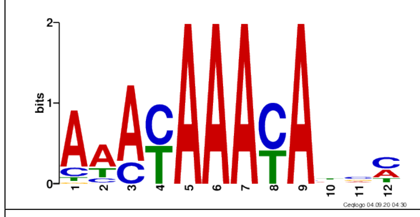	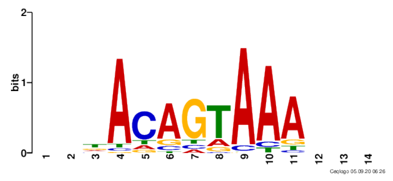	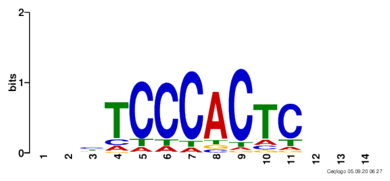	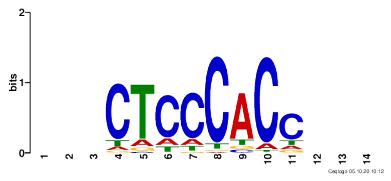
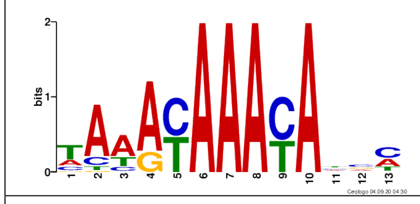	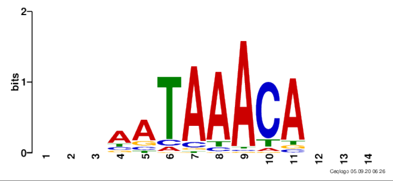		
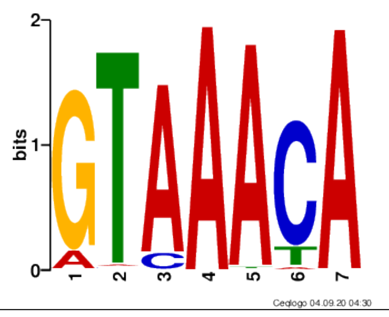	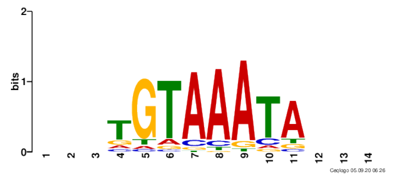		
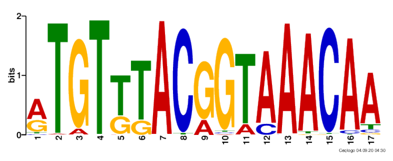	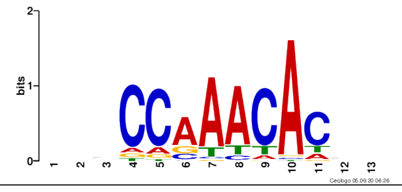		
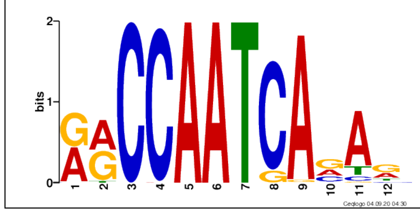	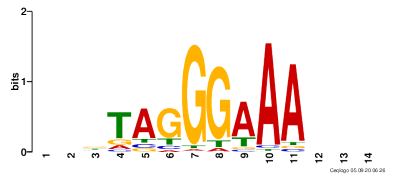		

Back to Table of Contents

32. ATF4, MOTIF .1.1.2.1

Fractions of oligos explained (independently of CIS-BP motifs): 7.0936%
Distance: 0.242087336581
p-value: 0.000019

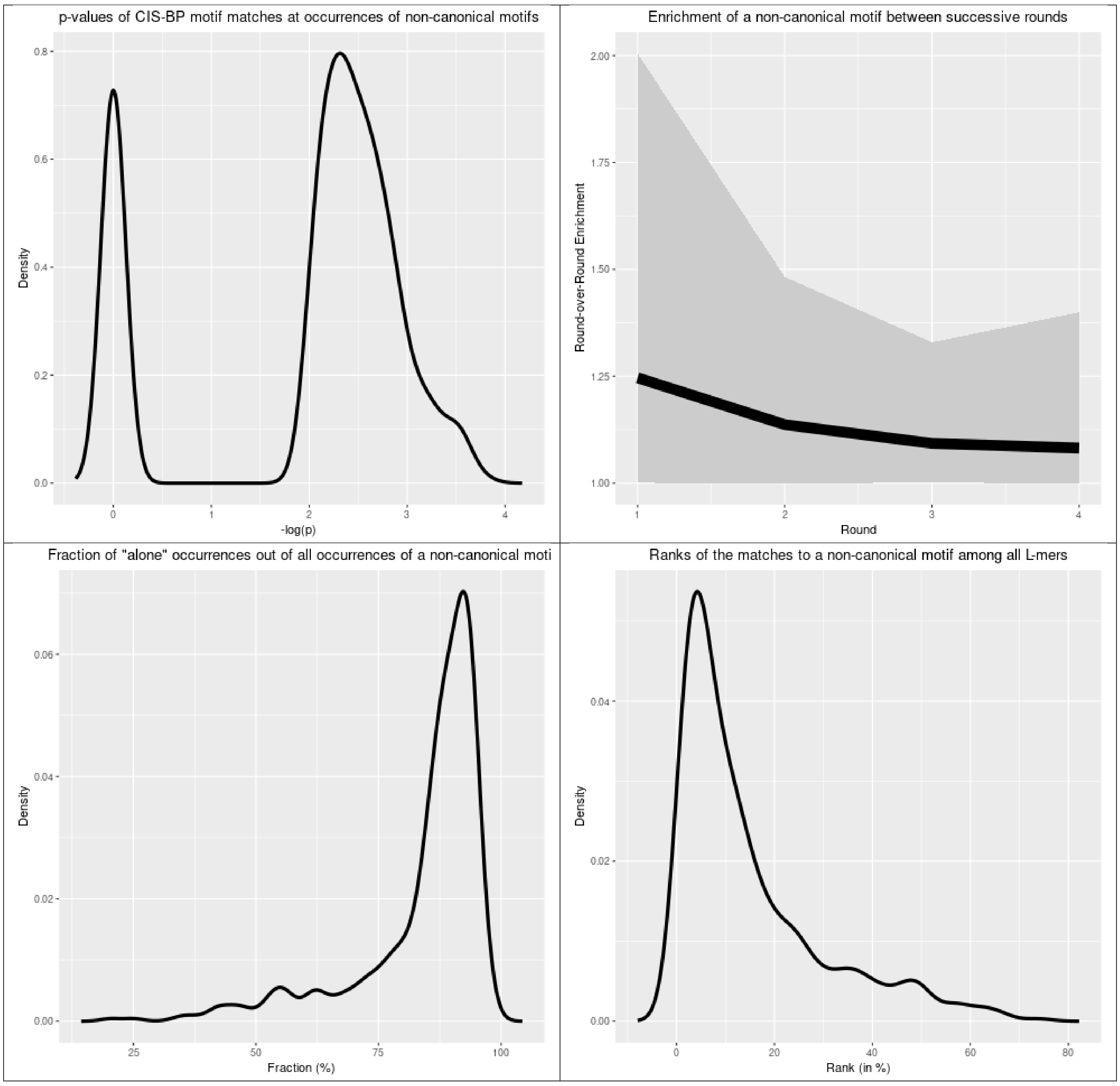

CIS-BP	Canonical	Non-canonical	Yin et al.
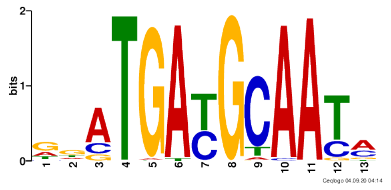	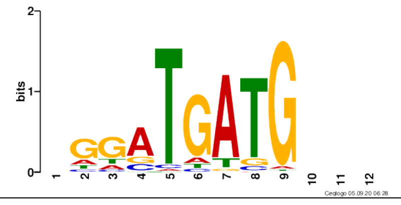	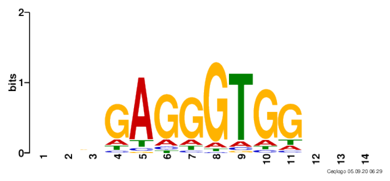	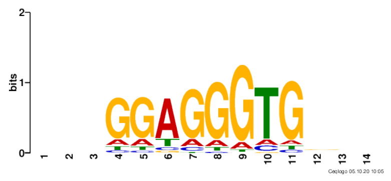
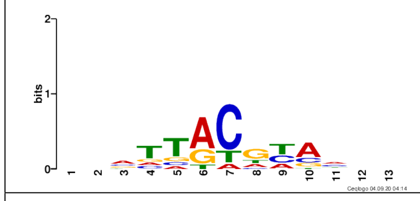	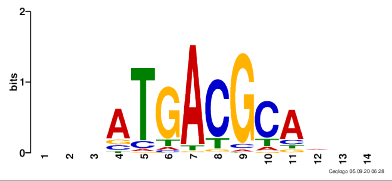		
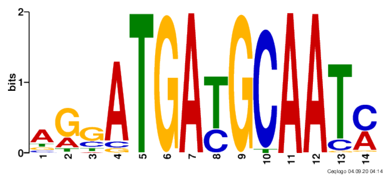	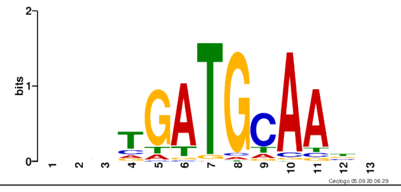		
	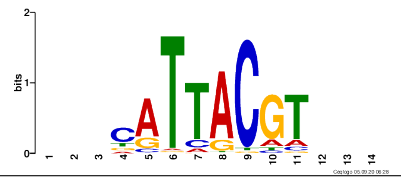		
	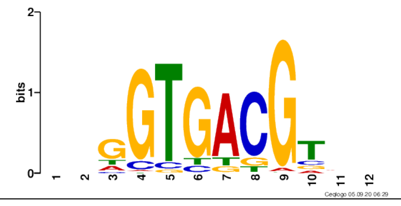		

Back to Table of Contents

33. ATF4, motif .1.1.2.1.3.1

Fractions of oligos explained (independently of CIS-BP motifs): 5.9609%
Distance: 0.231935466934
p-value: 0.000068

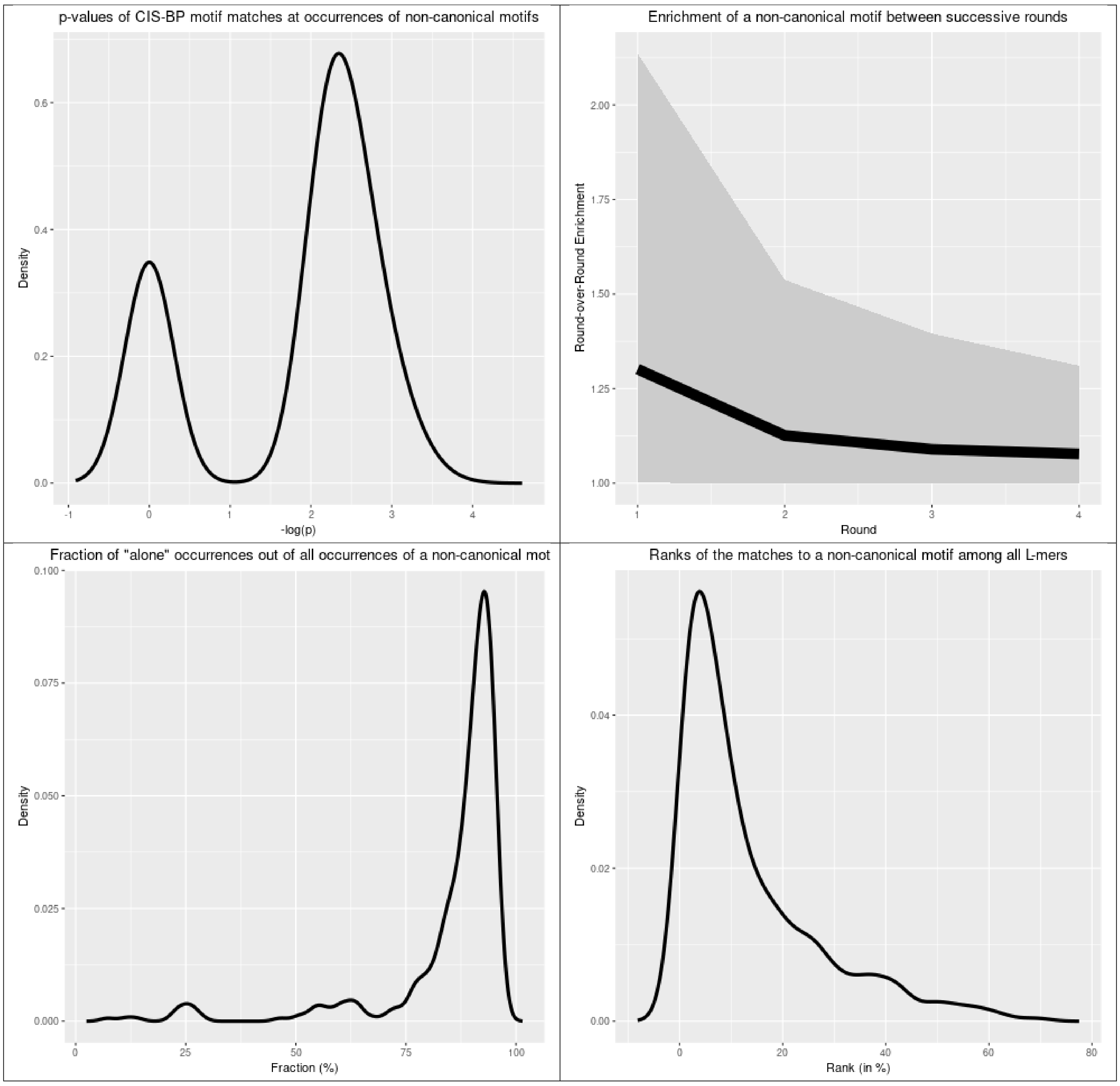

CIS-BP	Canonical	Non-canonical	Yin et al.
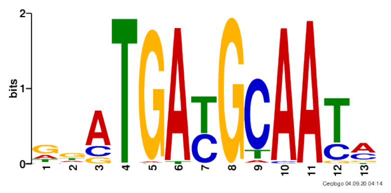	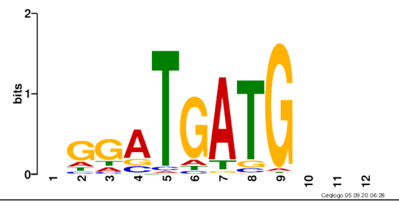	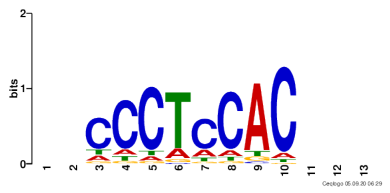	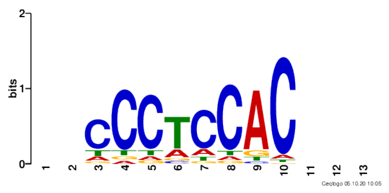
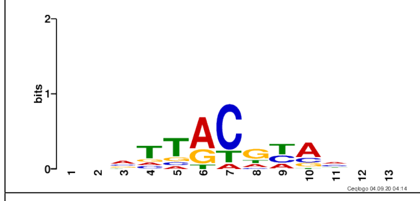	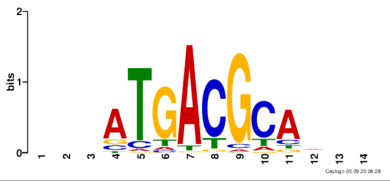		
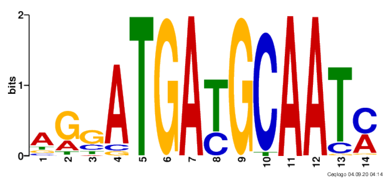	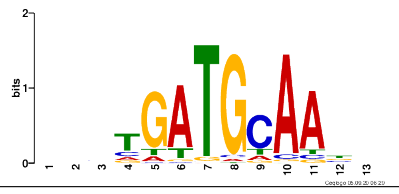		
	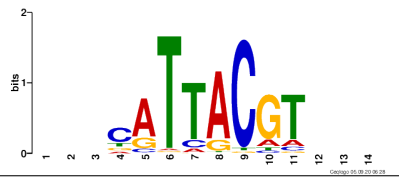		
	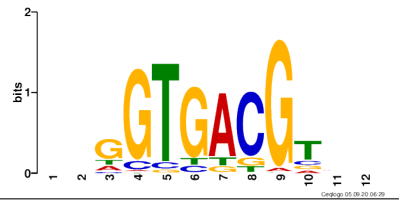		

Back to Table of Contents

34. ATF4, motif .1.1

Fractions of oligos explained (independently of CIS-BP motifs): 6.0716%
Distance: 0.192736432482
p-value: 0.004310

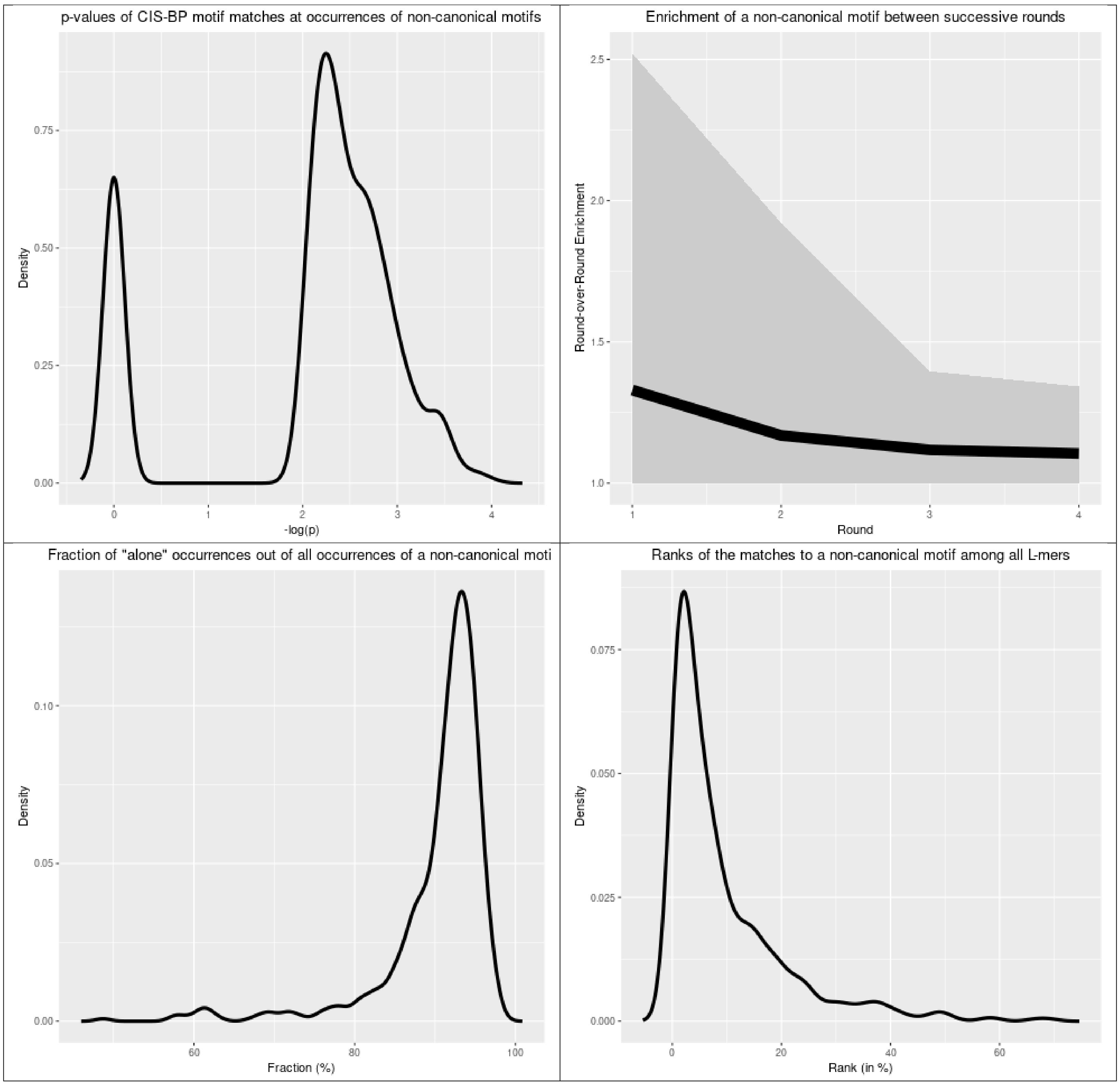

CIS-BP	Canonical	Non-canonical	Yin et al.
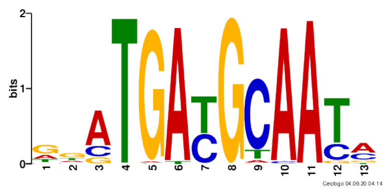	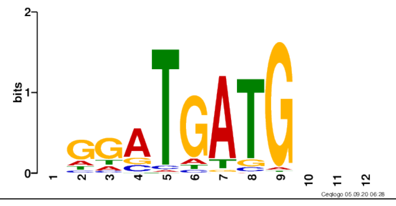	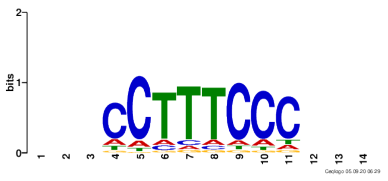	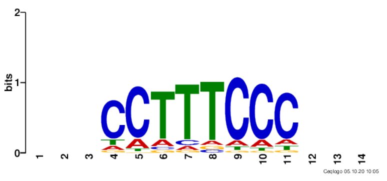
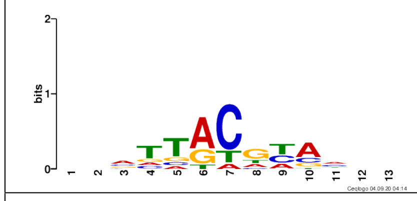	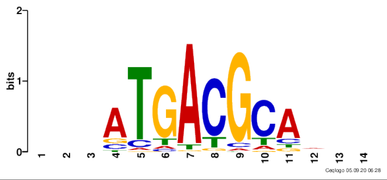		
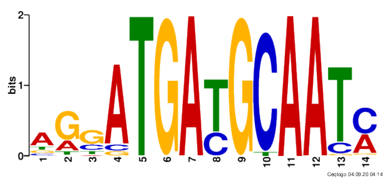	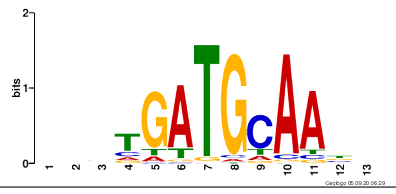		
	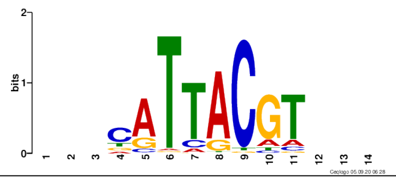		
	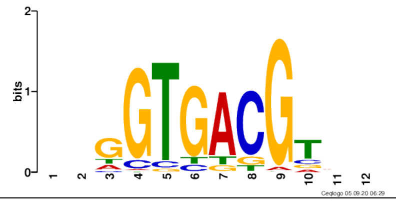		

Back to Table of Contents

35. NOTO, motif .1.1

Fractions of oligos explained (independently of CIS-BP motifs): 16.0274%
Distance: 0.270328800059
p-value: 0.000000

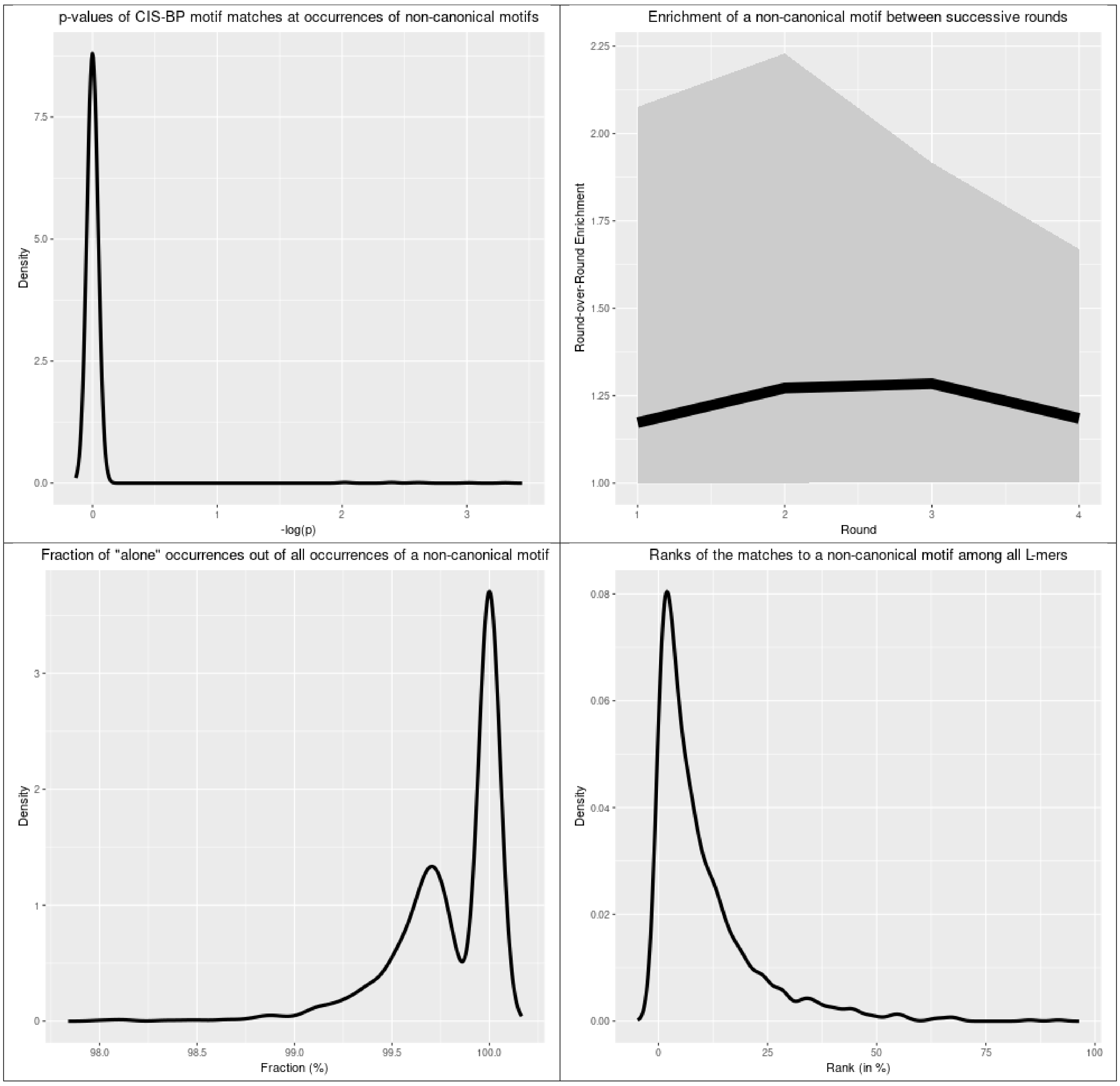

CIS-BP	Canonical	Non-canonical	Yin et al.
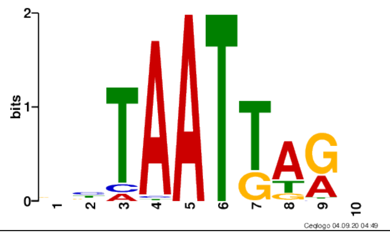	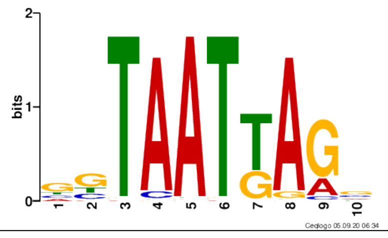	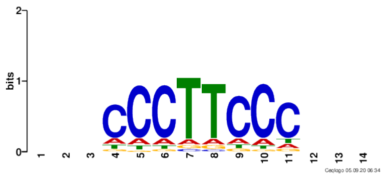	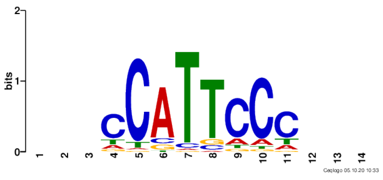
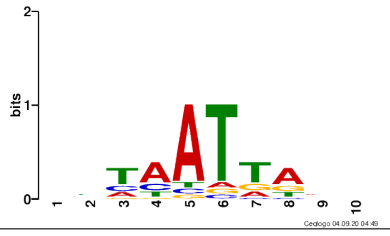			

Back to Table of Contents

36. SPDEF, motif .1.1

Fractions of oligos explained (independently of CIS-BP motifs): 11.3317%
Distance: 0.199927911906
p-value: 0.000267

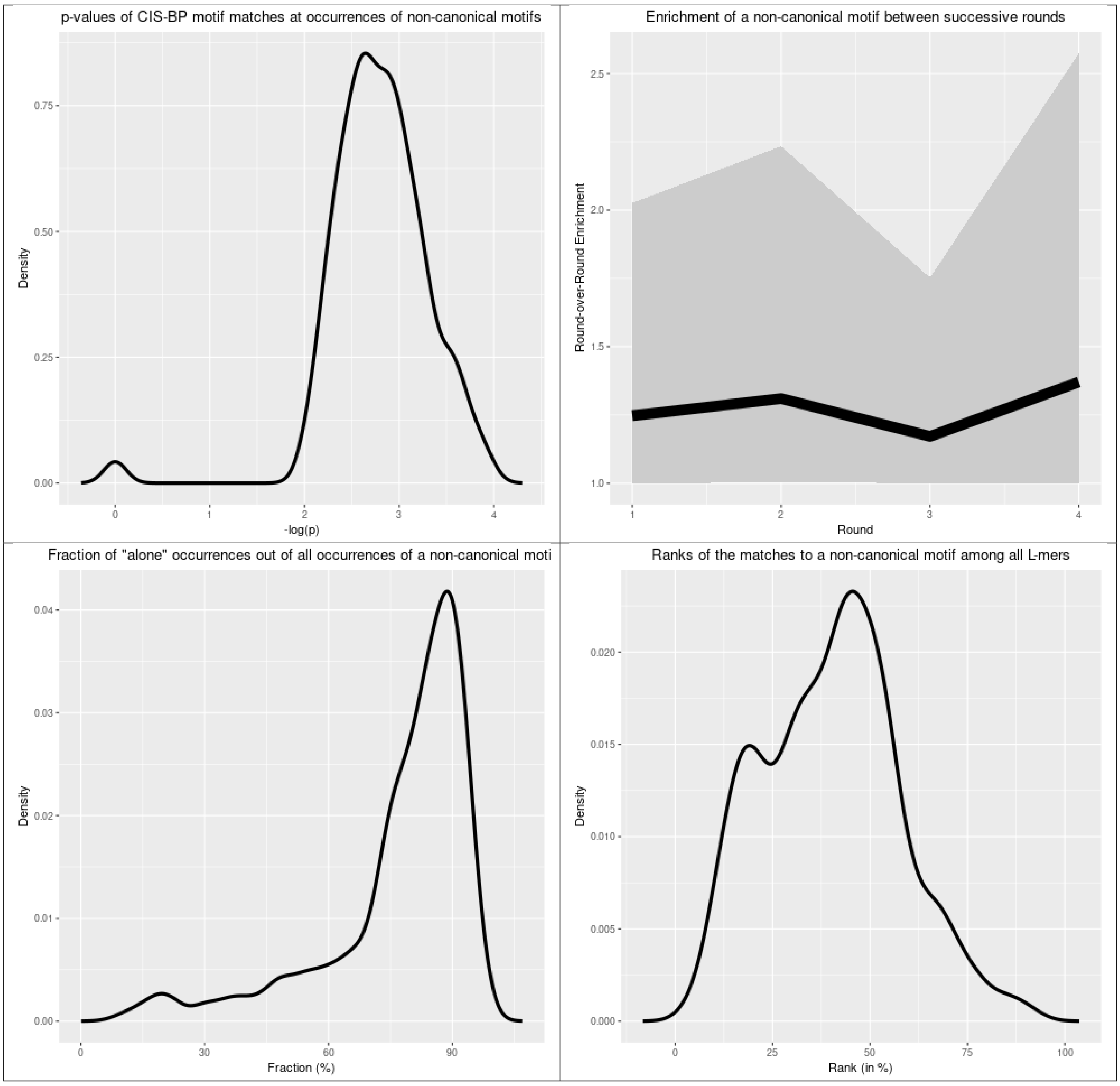

CIS-BP	Canonical	Non-canonical	Yin et al.
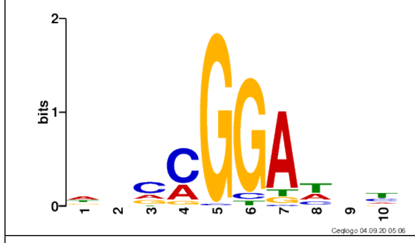	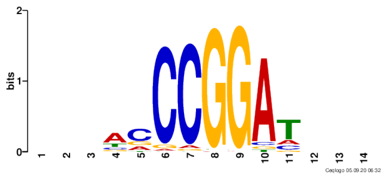	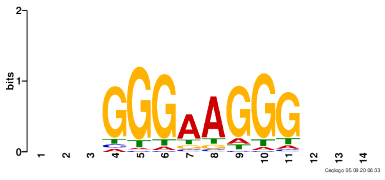	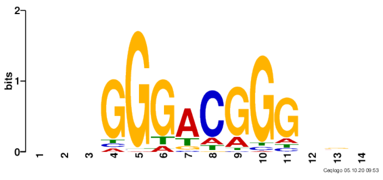
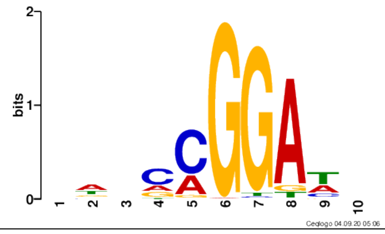	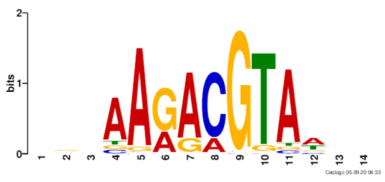		
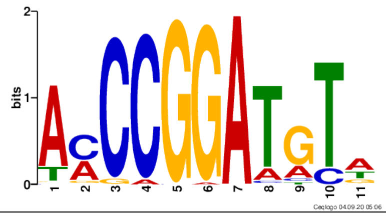	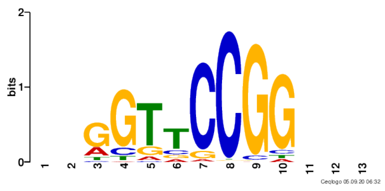		
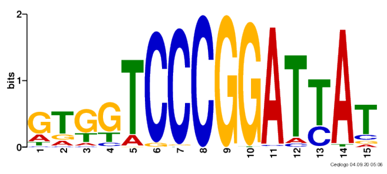	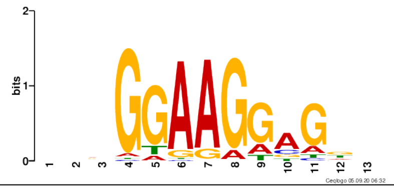		
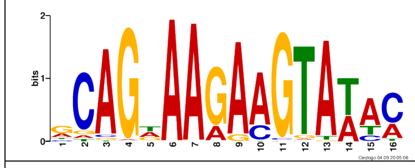			
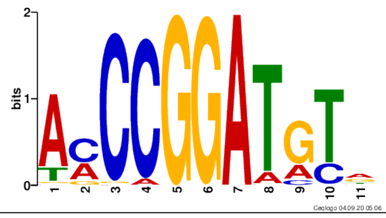			
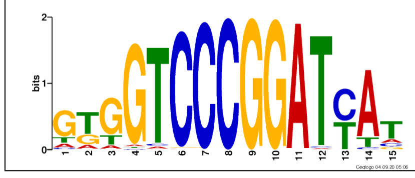			

CIS-BP	Canonical	Non-canonical	Yin et al.
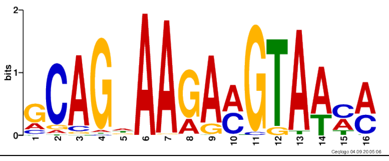			

Back to Table of Contents

37. KLF16, MOTIF .1.1.2.1

Fractions of oligos explained (independently of CIS-BP motifs): 8.7472%
Distance: 0.232856075664
p-value: 0.003652

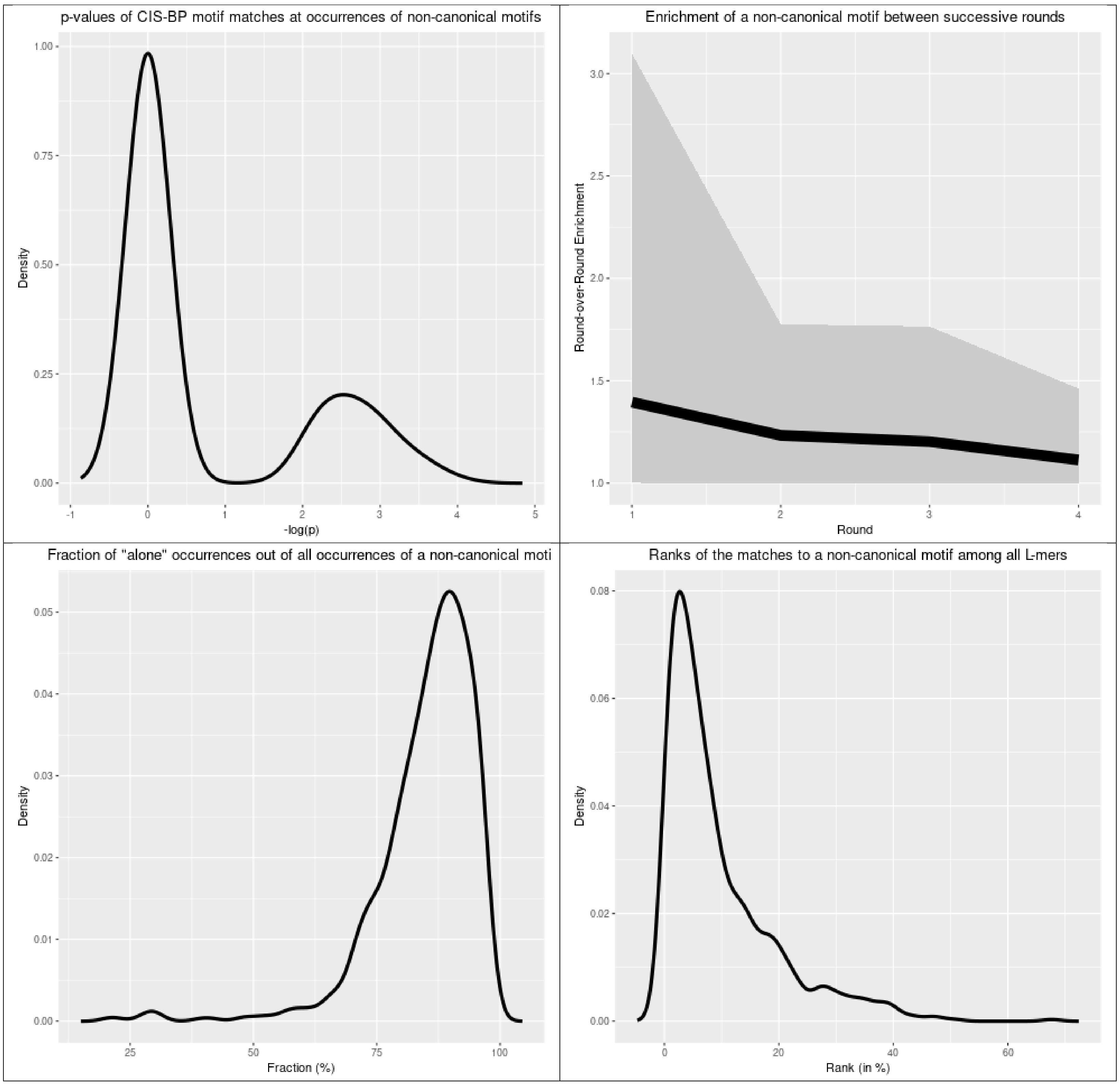

CIS-BP	Canonical	Non-canonical	Yin et al.
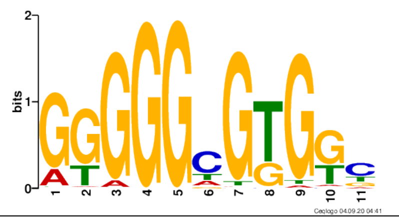	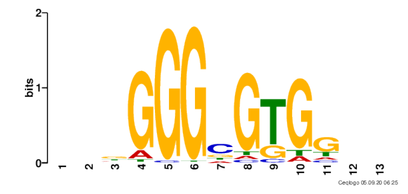	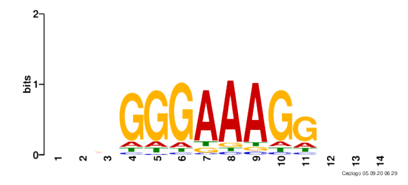	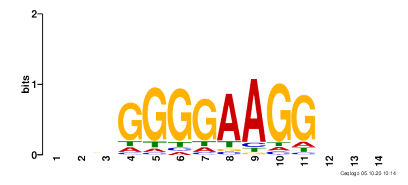
	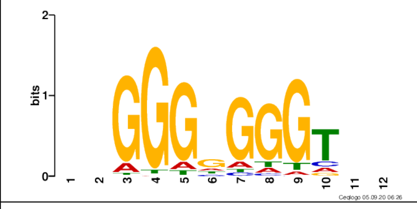		
	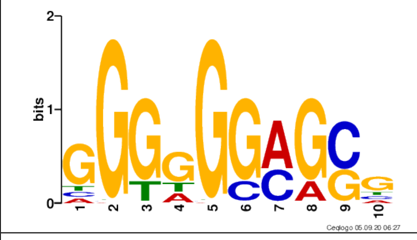		
	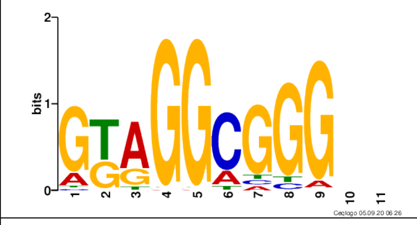		
	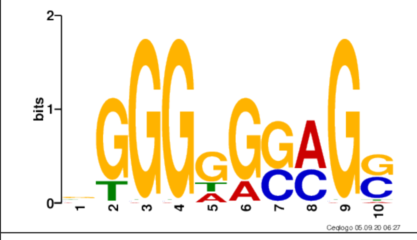		
	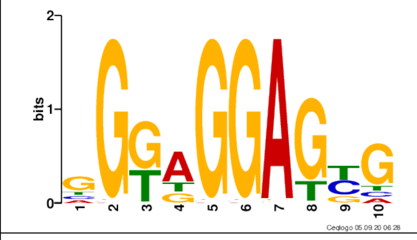		
	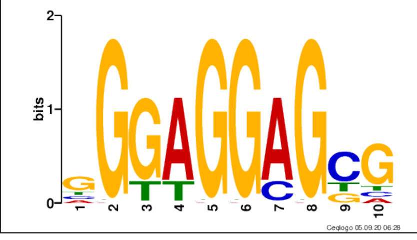		

Back to Table of Contents

38. FOXI1, MOTIF .1.1

Fractions of oligos explained (independently of CIS-BP motifs): 9.4099%
Distance: 0.249907251277
p-value: 0.001731

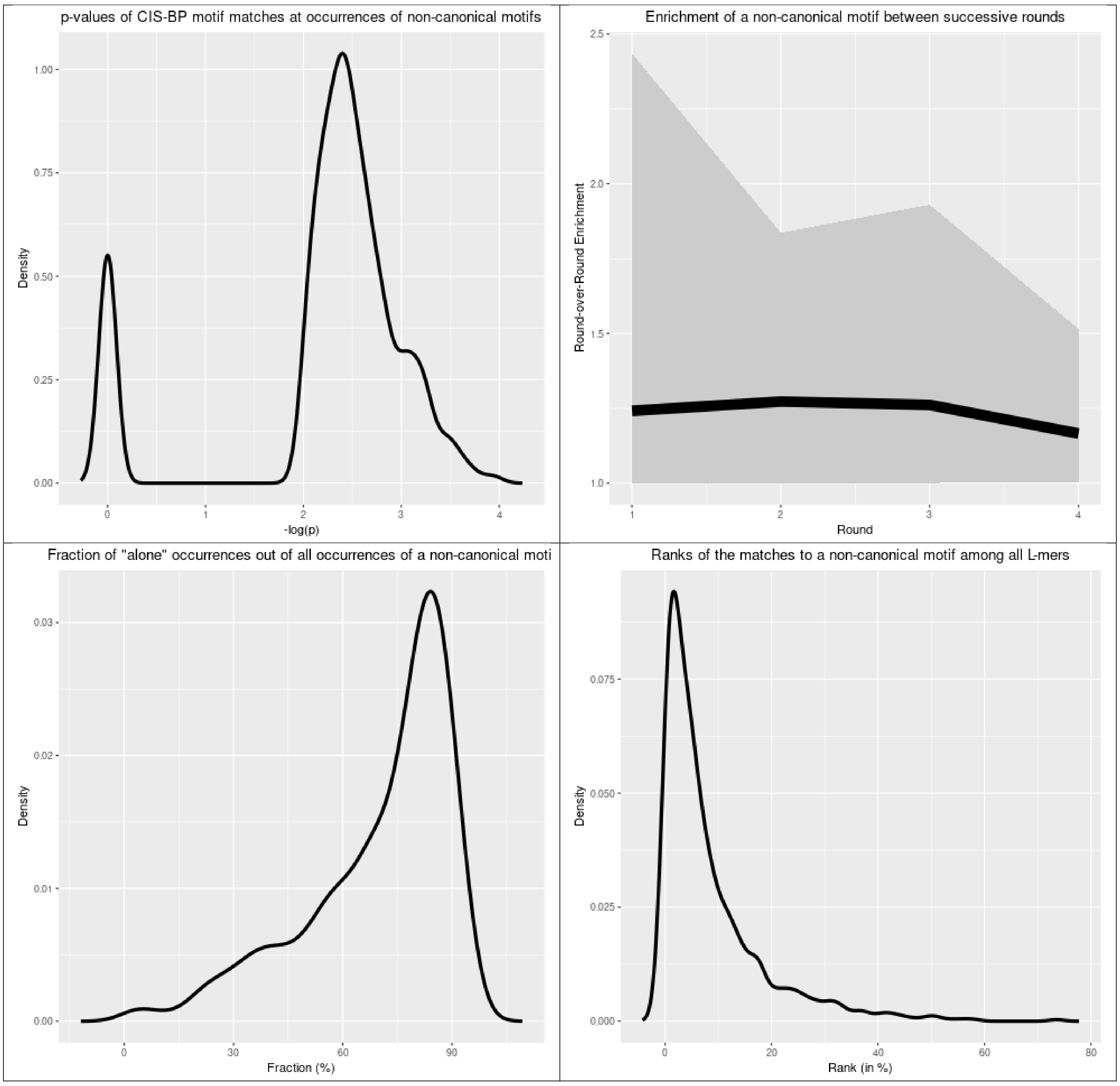

CIS-BP	Canonical	Non-canonical	Yin et al.
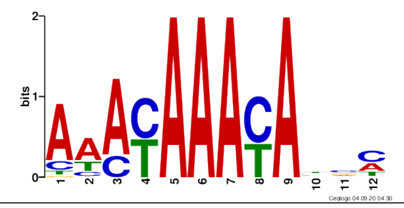	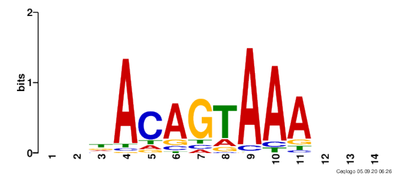	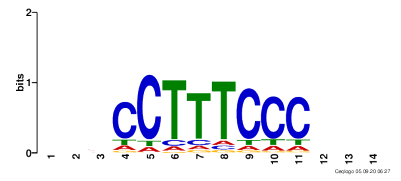	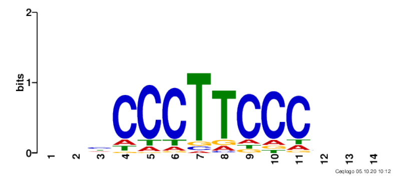
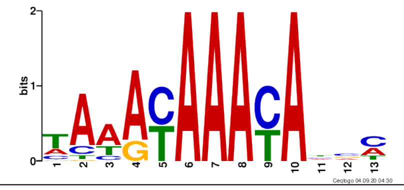	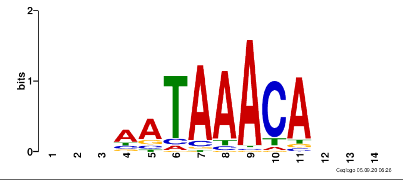		
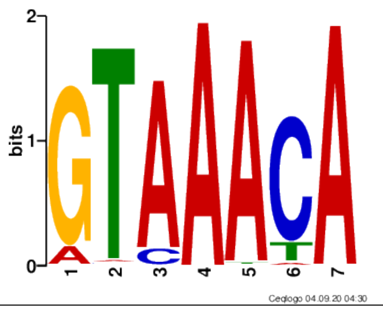	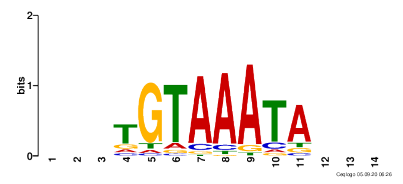		
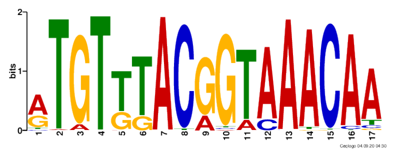	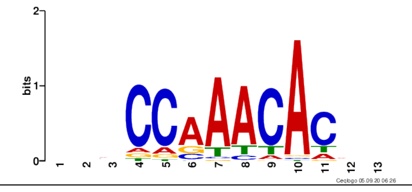		
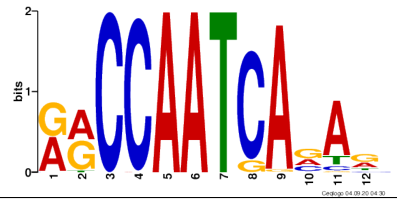	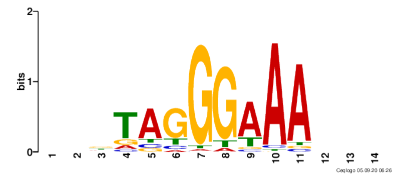		

Back to Table of Contents

39. NOTO, motif .1.1.2.1.3.1.4.1

Fractions of oligos explained (independently of CIS-BP motifs): 10.3657%
Distance: 0.255866283851
p-value: 0.000000

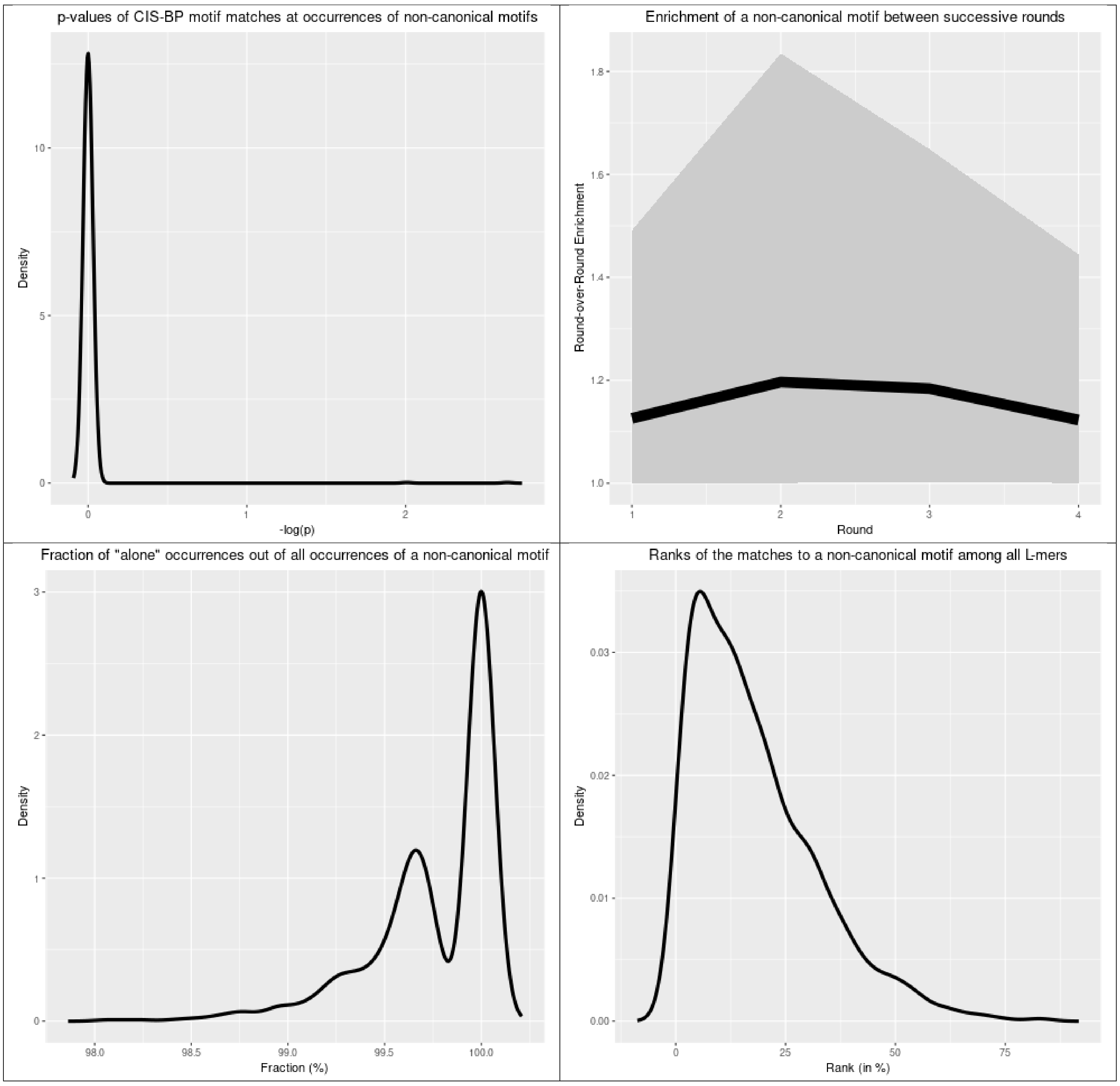

CIS-BP	Canonical	Non-canonical	Yin et al.
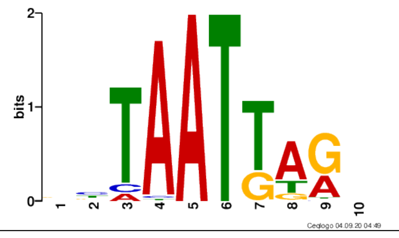	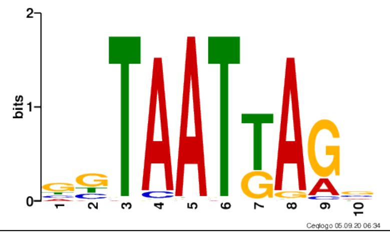	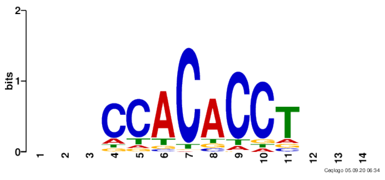	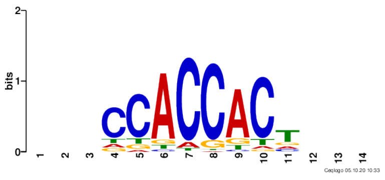
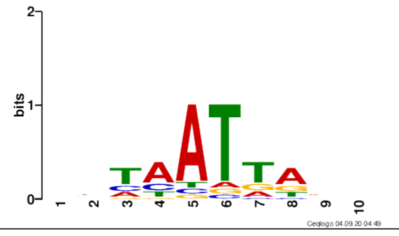			

Back to Table of Contents

40. POU3F2, motif .1.1

Fractions of oligos explained (independently of CIS-BP motifs): 5.9888%
Distance: 0.243654500991
p-value: 0.000001

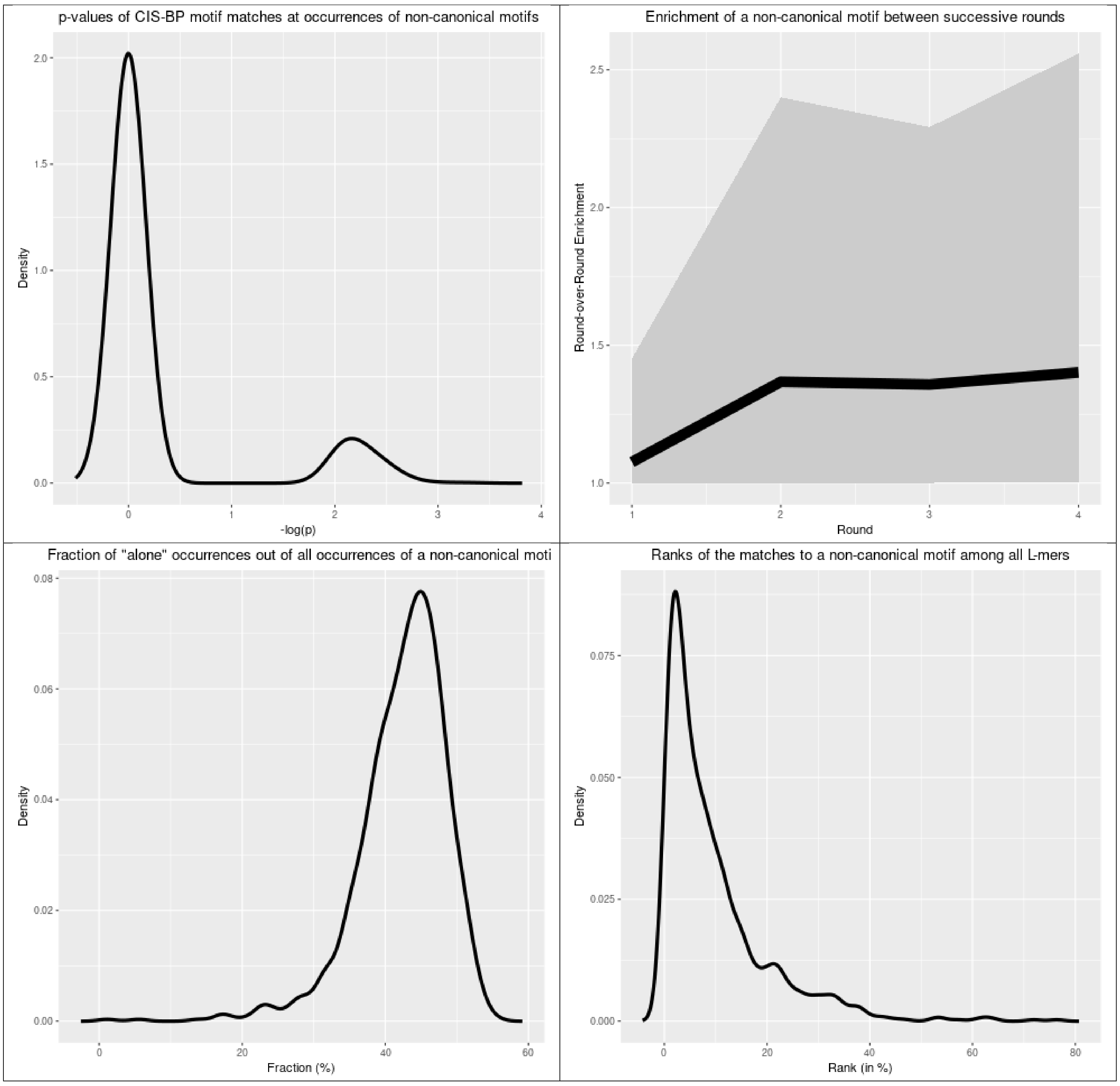

CIS-BP	Canonical	Non-canonical	Yin et al.
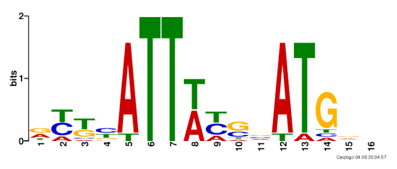	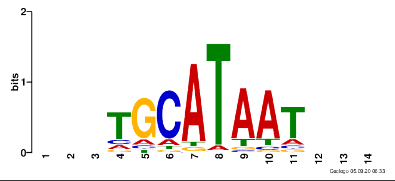	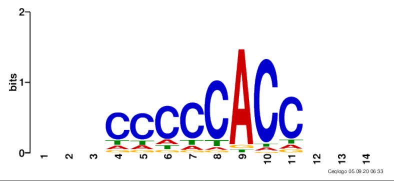	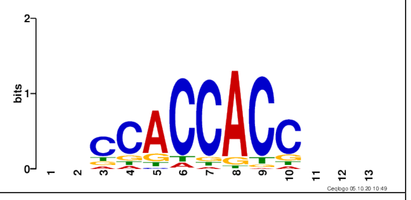
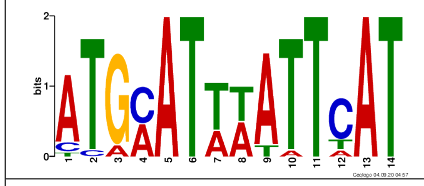	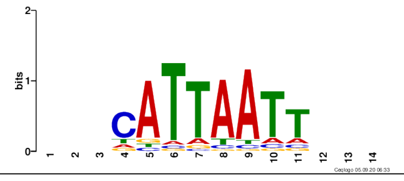		
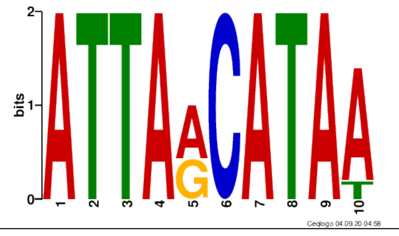	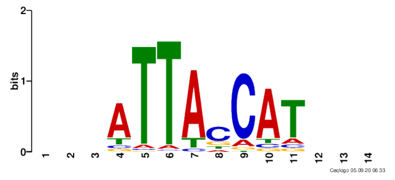		
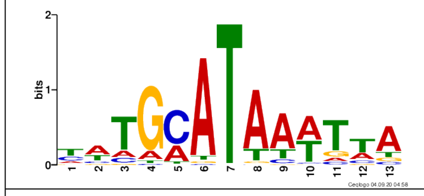	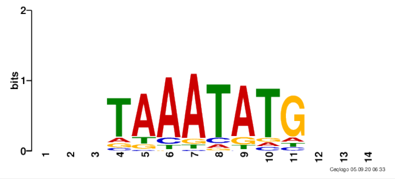		
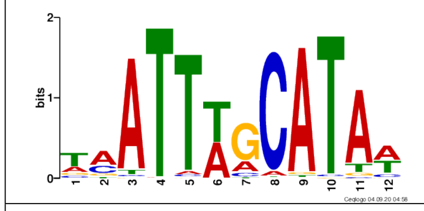			
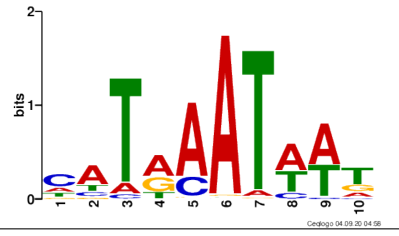			
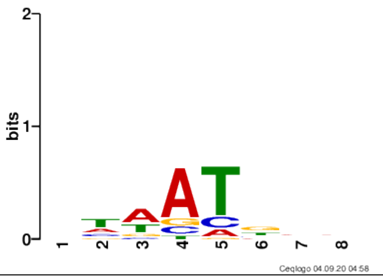			

Back to Table of Contents

41. FOXB1, motif .1.1

Fractions of oligos explained (independently of CIS-BP motifs): 6.9160%
Distance: 0.242933892645
p-value: 0.000752

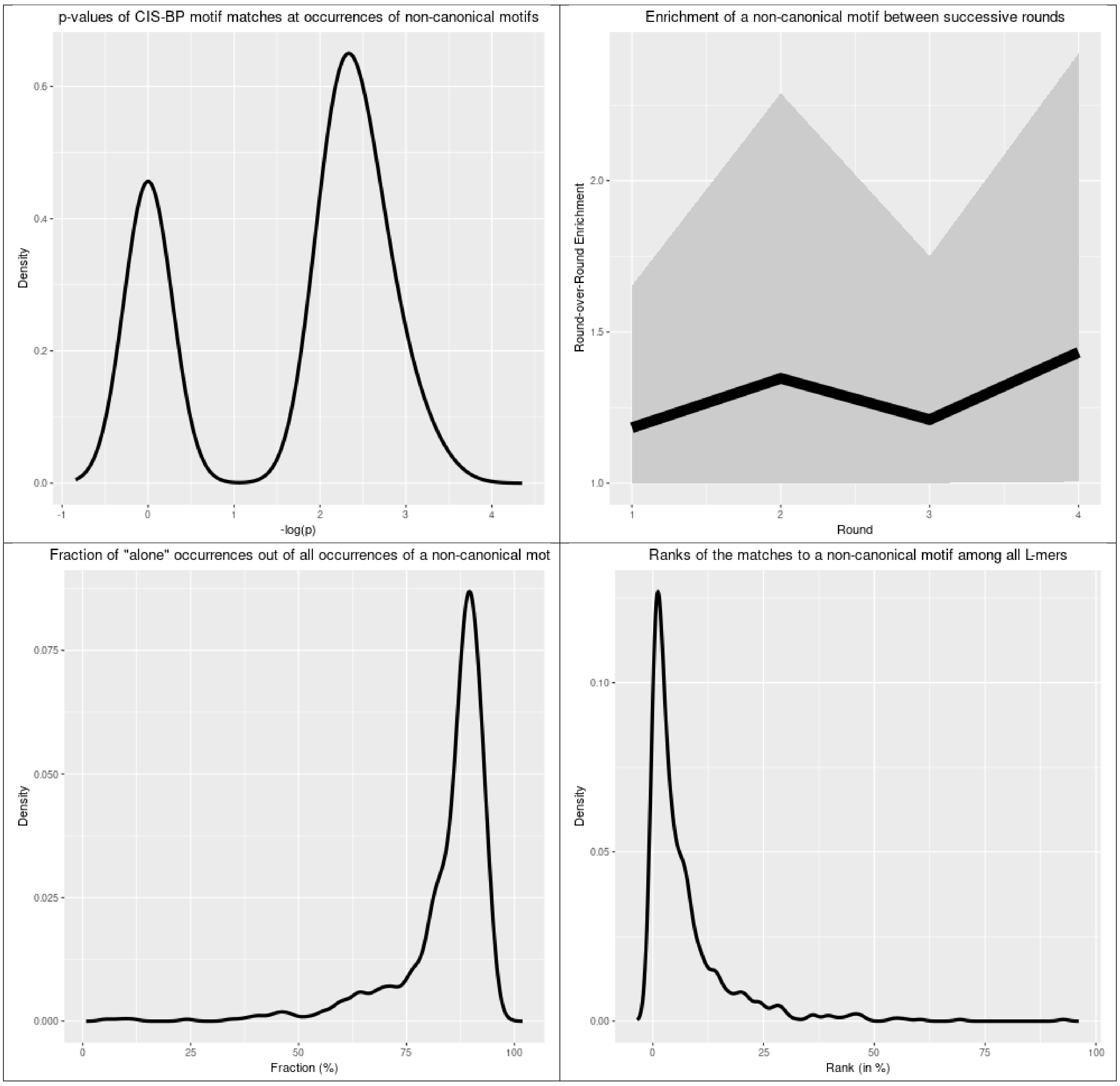

CIS-BP	Canonical	Non-canonical	Yin et al.
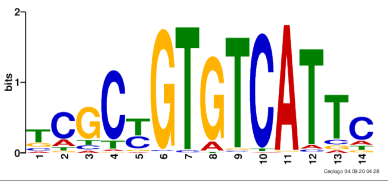	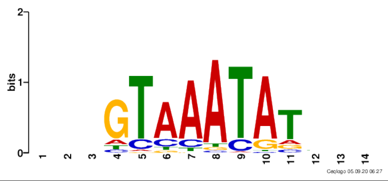	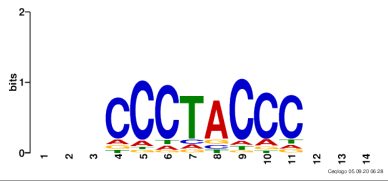	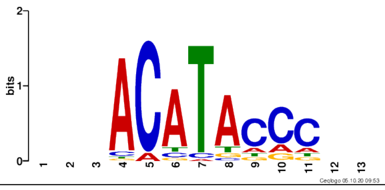
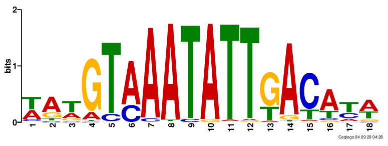	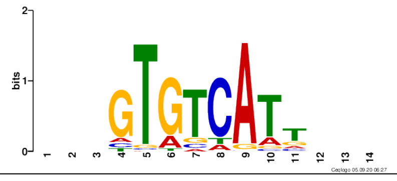		
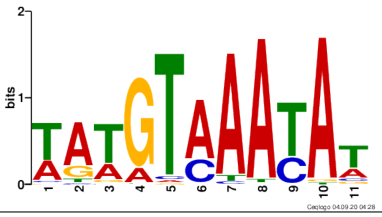	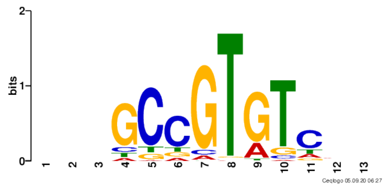		
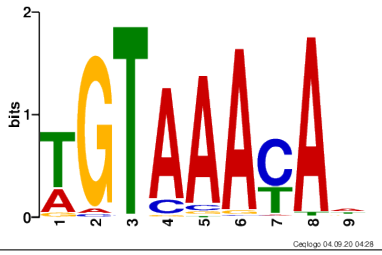	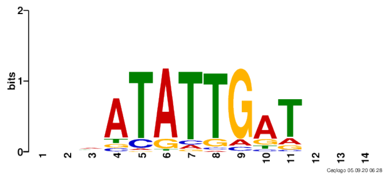		
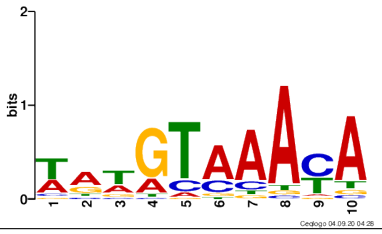	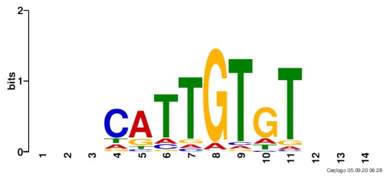		

Back to Table of Contents
