# Supplementary material for: Systematic identification of non-canonical transcription factor motifs
Source: BMC Mol Cell Biol. 2021 Aug 31;22:44. doi: 10.1186/s12860-021-00382-6 (PMC8408965; doi:10.1186/s12860-021-00382-6)
Supplement: Supplementary file 3 — Additional file 3: Supplementary Text 1. [file 12860_2021_382_MOESM3_ESM.docx]

# Supplementary Text 1

Our validation against Yin et al.’s data discovered the same (or very similar) non-canonical motifs for 42 of the 47 non-canonical motifs that we found in Yang et al.’s data [(Yang et al. 2017)](https://paperpile.com/c/KGdELA/itRZ3). We show the results and comparisons in Supplementary Table 1. Of the 42 motifs, we found four cases with a single base change in the consensus sequence of the non-canonical motif, one case of two base changes in the consensus sequence, and three cases of a long overlap (5 of 8 bases) in the consensus sequence. The remaining 34 cases were the same.
