# Supplementary material for: Systematic identification of non-canonical transcription factor motifs
Source: BMC Mol Cell Biol. 2021 Aug 31;22:44. doi: 10.1186/s12860-021-00382-6 (PMC8408965; doi:10.1186/s12860-021-00382-6)
Supplement: Supplementary file 4 — Additional file 4: Supplementary Text 2. [file 12860_2021_382_MOESM4_ESM.docx]

# Supplementary Text 2

We used the following three settings for control analysis: (i) dinucleotide shuffled versions of ChIP-peaks, (ii) randomly selected genomic sequences matched for length, GC-content, and repeat content (using gkmSVM [(Ghandi et al. 2016)](https://paperpile.com/c/KGdELA/B2dDe)), and (iii) shuffled versions of L-mers constituting the motifs.

We found that: (i) non-canonical motifs are enriched in ChIP-peaks compared to control sequences and (ii) non-canonical motifs’ L-mers are enriched than their shuffled sequences in ChIP-peaks (enrichment statistic $e(m)>1$, Supplementary Figure 1). Of note, for HOXB2 and ETV4, both canonical and non-canonical motifs showed mixed trends of $e(m)$ values. First, the non-canonical HOXB2 motif showed $e(m)<1$ in control setting (ii), but showed $e(m)>1$ in the other two settings. The canonical HOXB2 motif, on the other hand, showed $e(m)<1$ in settings (i) and (ii). Secondly, the non-canonical ETV4 motif showed $e(m)<1$ in setting (iii), but showed $e(m)>1$ in the other two settings. Interestingly, the canonical ETV4 motif showed $e(m)<1$ in setting (i).
