# Supplementary material for: Systematic identification of non-canonical transcription factor motifs
Source: BMC Mol Cell Biol. 2021 Aug 31;22:44. doi: 10.1186/s12860-021-00382-6 (PMC8408965; doi:10.1186/s12860-021-00382-6)
Supplement: Supplementary file 5 — Additional file 5: Supplementary Figure 1. Scatterplots showing the enrichments of non-canonical motifs against enrichments of canonical motifs in ChIP-seq data using three different control sequences: (a) dinucleotide shuffled versions of ChIP-peaks, (b) randomly selected genomic sequences matched for length, GC-content, and repeat-content, and (c) shuffled sequences of L-mers constituting the non-canonical and the canonical motifs. [file 12860_2021_382_MOESM5_ESM.docx]

| (a) | 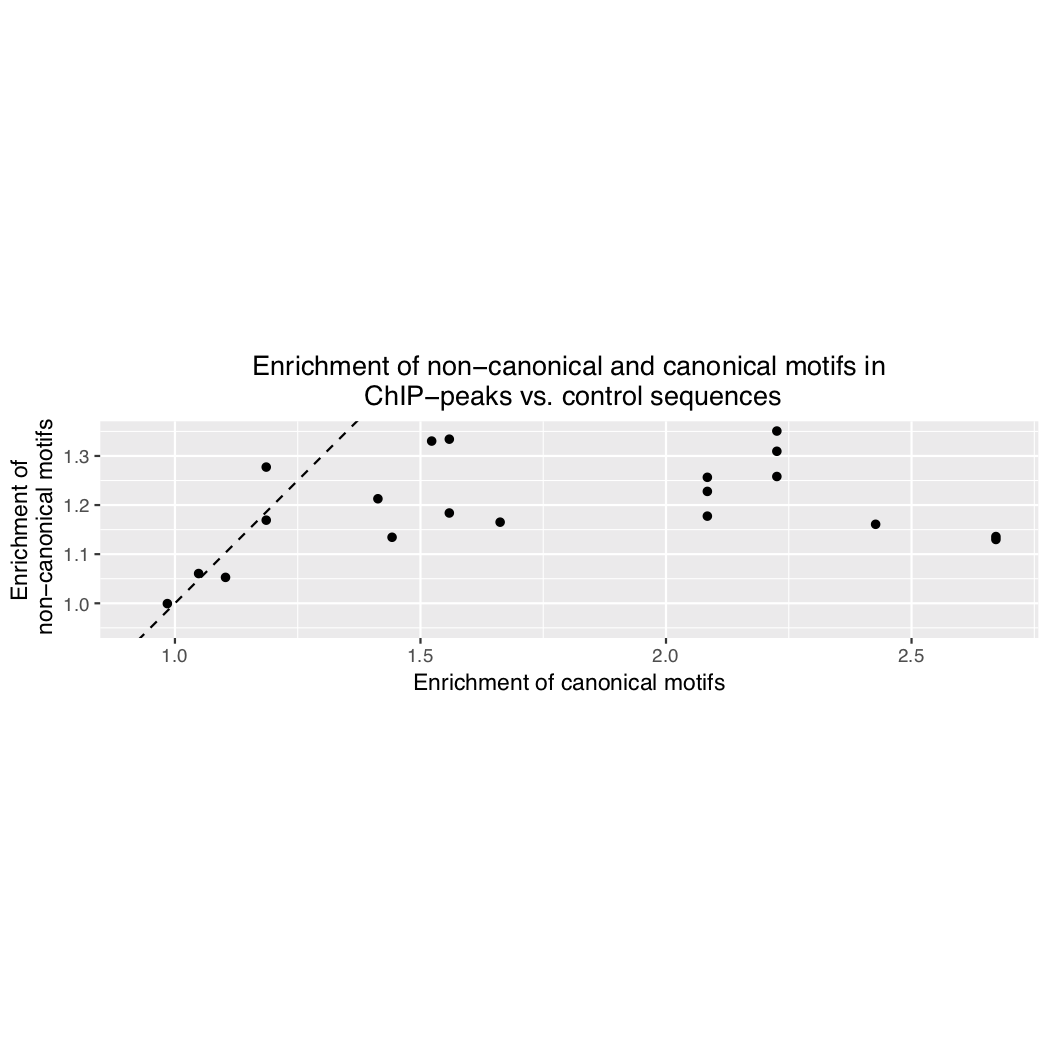 |
| --- | --- |
| (b) | 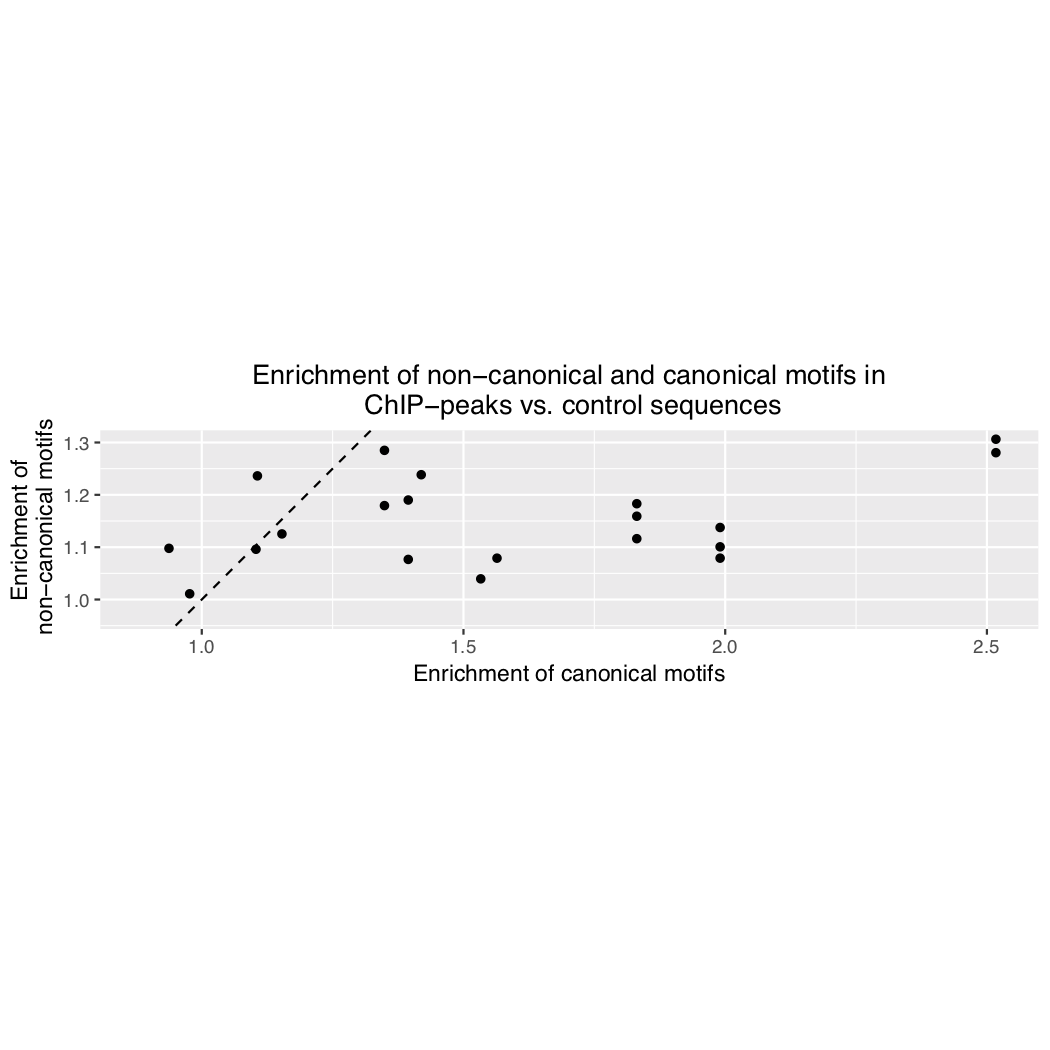 |
| (c)  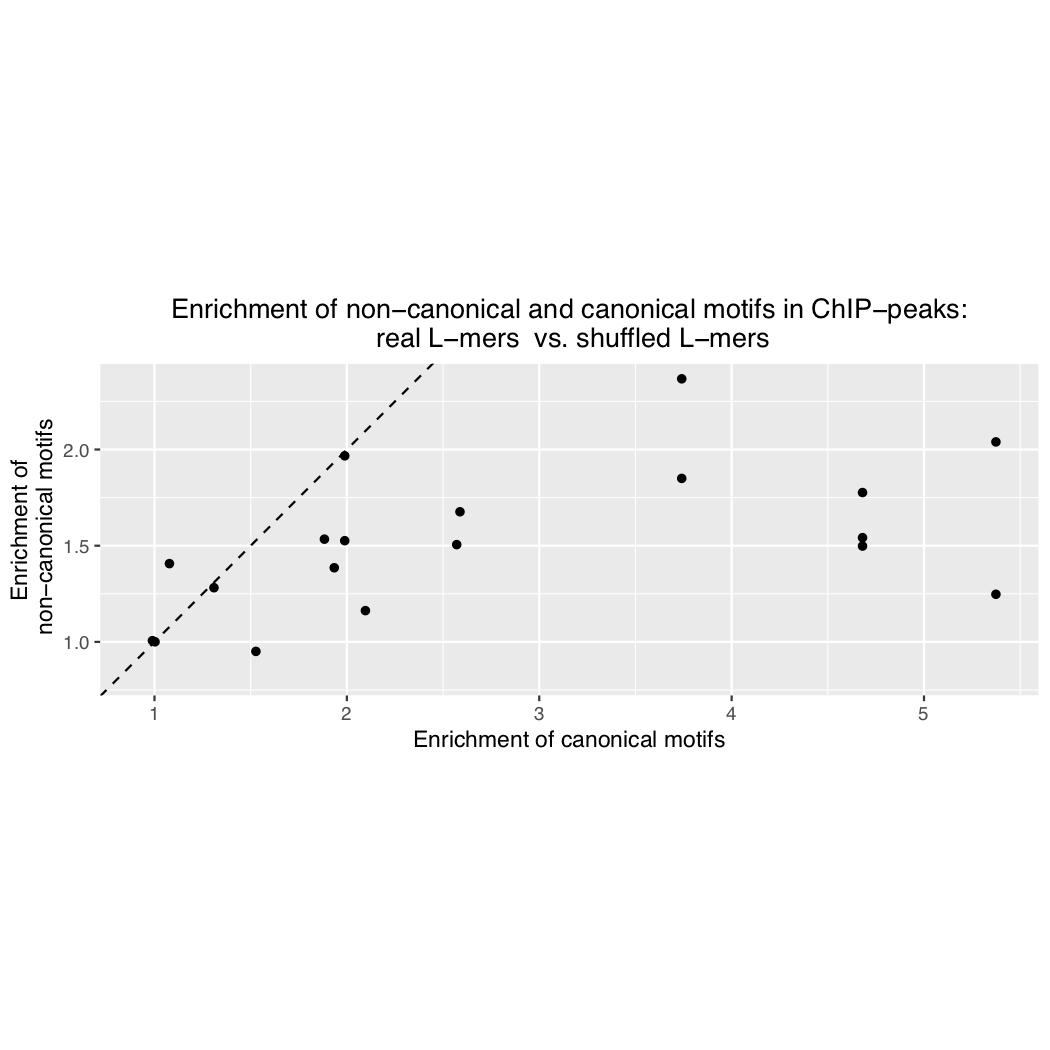 | |
| Supplementary Figure 1. Scatterplots showing the enrichments of non-canonical motifs against enrichments of canonical motifs in ChIP-seq data using three different control sequences: (a) dinucleotide shuffled versions of ChIP-peaks, (b) randomly selected genomic sequences matched for length, GC-content, and repeat-content, and (c) shuffled sequences of L-mers constituting the non-canonical and the canonical motifs. | |
