# Supplementary material for: Systematic identification of non-canonical transcription factor motifs
Source: BMC Mol Cell Biol. 2021 Aug 31;22:44. doi: 10.1186/s12860-021-00382-6 (PMC8408965; doi:10.1186/s12860-021-00382-6)
Supplement: Supplementary file 6 — Additional file 6: Supplementary Text 3. [file 12860_2021_382_MOESM6_ESM.docx]

# Supplementary Text 3

We found similar non-canonical motifs (consensus sequence CCCTTCCC or similar) for nine TFs that belong to unrelated families (Table 1 and Supplementary Table 1). These motifs validated in the Yin et al. data [(Yin et al. 2017)](https://paperpile.com/c/KGdELA/7McH0) and were not reported before as potential artifacts of HT-SELEX data. For example, Alipanahi et al. reported CCCNCCCNCCC motifs from HT-SELEX data of unrelated TF families [(Alipanahi et al. 2015)](https://paperpile.com/c/KGdELA/U9hQ5). They noted that appropriately selecting an early HT-SELEX round could avoid such motifs. Our analyzed rounds, i.e., the ones Yang et al. [(Yang et al. 2017)](https://paperpile.com/c/KGdELA/itRZ3) selected, are consistently the same (and earlier for NOTO and ZNF740) than Alipanahi et al.’s selected rounds. As such, we do not expect these motifs to be HT-SELEX artifacts. Still, we mark these motifs separately in Table 1 Fig. 3.
